# Phenotypic screening reveals a highly selective phthalimide-based compound with antileishmanial activity

**DOI:** 10.1371/journal.pntd.0012050

**Published:** 2024-03-25

**Authors:** Farnaz Zahedifard, Meenakshi Bansal, Neha Sharma, Sumit Kumar, Siqi Shen, Priyamvada Singh, Brijesh Rathi, Martin Zoltner

**Affiliations:** 1 Drug Discovery and Evaluation Unit, Department of Parasitology, Faculty of Science, Charles University in Prague, Biocev, Vestec, Czech Republic; 2 H. G. Khorana Centre for Chemical Biology, Department of Chemistry, Hansraj College, University of Delhi, Delhi, India; 3 Department of Chemistry, Deenbandhu Chhotu Ram, University of Science & Technology, Murthal, Sonepat Haryana, India; 4 Department of Chemistry, Miranda House, University of Delhi, Delhi, India; 5 Delhi School of Public Health, Institution of Eminence, University of Delhi, Delhi, India; Oswaldo Cruz Foundation: Fundacao Oswaldo Cruz, BRAZIL

## Abstract

Pharmacophores such as hydroxyethylamine (HEA) and phthalimide (PHT) have been identified as potential synthons for the development of compounds against various parasitic infections. In order to further advance our progress, we conducted an experiment utilising a collection of PHT and HEA derivatives through phenotypic screening against a diverse set of protist parasites. This approach led to the identification of a number of compounds that exhibited significant effects on the survival of *Entamoeba histolytica*, *Trypanosoma brucei*, and multiple life-cycle stages of *Leishmania spp*. The *Leishmania* hits were pursued due to the pressing necessity to expand our repertoire of reliable, cost-effective, and efficient medications for the treatment of leishmaniases. Antileishmanials must possess the essential capability to efficiently penetrate the host cells and their compartments in the disease context, to effectively eliminate the intracellular parasite. Hence, we performed a study to assess the effectiveness of eradicating *L*. *infantum* intracellular amastigotes in a model of macrophage infection. Among eleven *L*. *infantum* growth inhibitors with low-micromolar potency, PHT-39, which carries a trifluoromethyl substitution, demonstrated the highest efficacy in the intramacrophage assay, with an EC50 of 1.2 +/- 3.2 μM. Cytotoxicity testing of PHT-39 in HepG2 cells indicated a promising selectivity of over 90-fold. A chemogenomic profiling approach was conducted using an orthology-based method to elucidate the mode of action of PHT-39. This genome-wide RNA interference library of *T*. *brucei* identified sensitivity determinants for PHT-39, which included a P-type ATPase that is crucial for the uptake of miltefosine and amphotericin, strongly indicating a shared route for cellular entry. Notwithstanding the favourable properties and demonstrated efficacy in the *Plasmodium berghei* infection model, PHT-39 was unable to eradicate *L*. *major* infection in a murine infection model of cutaneous leishmaniasis. Currently, PHT-39 is undergoing derivatization to optimize its pharmacological characteristics.

## Author summary

Two novel compound series, based on hydroxyethylamine (HEA) and phthalimide (PHT) pharmacophores, previously led to the discovery of antiplasmodial lead compounds, both with therapeutic efficacy in a murine *Plasmodium* infection model. Here we employed these compound libraries, supplemented with newly designed HEA derivatives, for phenotypic screening against a wide range of protist parasites associated with unmet medical needs. We report potent hit compounds for *Entamoeba histolytica*, *Trypanosoma brucei and Leishmania* spp., with limited overlap in their structure–activity relation. PHT-39 efficiently penetrated host macrophages to effectively eliminate *Leishmania* intracellular amastigotes, while displaying high selectivity. Leveraging an orthology-based genome-wide RNAi library screen, we propose an uptake route for the PHT-39 that relies on a transmembrane flippase, known as crucial uptake transporter for the two frontline antileishmanials miltefosine and amphotericin. Despite favourable drug properties of PHT-39, experimental PHT-39 treatment failed to eliminate the *Leishmania* infection in a cutaneous leishmaniasis animal model. Considering PHT-39 high selectivity and the ability of the drug to effectively reach the intracellular stage of the parasite, we suggest PHT-39 for further optimization.

## Introduction

Neglected tropical diseases remain a threat to public health and responsible for high mortality, morbidity, and economic losses among the world’s poorest populations. With gradual global warming, insect vectors and, consequently, their infectious parasites are spreading from tropical and sub-tropical areas to new territories around the globe [1]. Importantly, available treatment options are extremely limited, ineffective, or riddled with toxicity and resistance issues. Generally, there are few new drugs in the development pipeline, which is reflecting the missing financial incentive for pharmaceutical companies to invest in this area of research [2]. Hence, we set out to subject a library of novel compound classes to phenotypic screening in a range of unicellular parasites causative of diseases associated with unmet medical needs.

We chose to target the Kinetoplastida pathogens *Trypanosoma brucei*, *Trypanosoma cruzi* and *Leishmania* spp. causing African trypanosomiasis (HAT), Chagas disease and the leishmaniases, respectively. All are classified by the WHO as neglected tropical diseases (WHO, 2021) [3] and remain a persistent global problem, particularly, in rural areas with high poverty rates. The leishmaniases constitute a spectrum of diseases ranging from the usually self-healing but potentially disfiguring cutaneous leishmaniasis, over the highly disfiguring mucocutaneous, to the visceral leishmaniasis (VL), which is invariably fatal if left untreated. An estimated 600 million people are at risk of VL according to the WHO and there are 50,000–90,000 new cases per year, giving rise to 26,000 to 65,000 deaths annually. Currently, available treatments have serious limitations ranging from adverse toxicity over complex administration to emerging resistance [4]. *Leishmania* parasites have a complex life cycle consisting of two major stages. The promastigote stage describes the insect form which is injected into the mammalian host through the bite initiating the sandfly blood meal. Upon entering the bite-wound, promastigotes are readily phagocytised by mononuclear phagocytic cells, primarily macrophages. Evading lysis, the parasite cells then transform into the morphologically distinct amastigote stage that is proliferating within the parasitophorous vacuole (PV), a phagolysosome-like structure that amastigotes exploit for sheltering. This acidic compartment shares late endosomal/lysosomal characteristics, and there is multiple evidence that the PV is modulated by the intracellular parasite [5].

Chagas disease affects about 8–10 million people and is responsible for 14,000 annual deaths [6] the costs of treatment, and lost productivity are estimated to amount to 7 billion US$ per year [7]. HAT treatment, until recently, relied on only a few old-fashioned and highly toxic drugs. The situation has improved with the recent development of new treatment-regimens (eflornithine/nifurtimox combination treatment) and new drugs fed into the development pipeline, mainly driven by the Drugs for Neglected Disease Initiative (DNDi, https://dndi.org) and partners. One is the recently approved fexinidazole, a highly promising oral drug effective against both stages of sleeping sickness caused by *T*. *brucei gambiense* [8]. Still, the reductive prodrug-activation dependency of fexinidazole, shared with other nitroimidazole drugs [9] makes it prone to future resistance formation. Moreover, animal African trypanosomiasis (AAT) remains a considerable economic burden in sub-Saharan Africa.

As a further target organism, we selected *Entamoeba histolytica*, an amoebozoa parasite colonizing the human gastrointestinal tract. Whilst in the majority of cases, human infection is asymptomatic, approximately 10% of patients develop amoebiasis and, in some cases severe extraintestinal disease. Current treatment options are reliant on a single drug class, the nitroimidazoles.

Previous studies have elucidated the potential of hydroxyethylamine (HEA) and phthalimide (PHT) compounds as anti-parasitic agents. These compounds demonstrate a wide range of mechanisms of action that are capable of targeting various stages of parasite development and interfering with crucial biological processes [10–13]. It is noteworthy that the progress made towards the development of PHT and HEA based compounds as potential anti-parasitic agents is currently in its emerging phase. Further, investigation is essential to ascertain their effectiveness, safety, and viability for preclinical and clinical development. In this paper, we curiously decided to perform phenotypic screening of our in-house library of 102 compounds based on HEA and PHT pharmacophores on various protist parasites (*i*.*e*., *Trypanosoma brucei*, *Trypanosoma cruzi*, *Leishmania* spp. and *Entamoeba histolytica*).

## Methods

### Ethics statement

All regulated procedures on living animals were carried out under the authority by the ethical committee of Charles University and authorities in the Ministry of Agriculture and the State Veterinary Administration of the Czech Republic (53659/2019-MZE-18134).

### General, chemicals and reagents

RPMI, glycerol, dimethyl sulfoxide (DMSO), 2-mercaptoethanol, Concanavalin A (ConA) and Penicillin-Streptomycin (Pen-Strep) were purchased from Sigma. Resazurin powder was bought from Chem Cruz. M199, Grace insect medium, MEM, fetal calf serum (FCS) and BME vitamins were provided from Gibco and Amikin from Medopharm. HMI-9 medium and PMA (Phorbol 12-myristate 13-acetate) were from Thermo Fischer Scientific.

Organic syntheses employed chemicals and solvents that were procured from reliable manufacturers, namely GLR, TCI, and Sigma Aldrich, without prior purification. In order to monitor the progress of the reaction, thin-layer chromatography (TLC) analysis was conducted utilizing 60 F254 silica gel plates (Sigma-Merck). Syntheses involved microwave reactions under controlled temperature programming conditions. The temperature was gradually increased over a period of 5 minutes and then maintained at 70°C for 10 minutes. The "Start Synth Microwave Synthesis Labstation" microwave equipment with a 300 W power supply was utilized for this purpose. NMR spectroscopy analysis (S1 Fig) employed tetramethylsilane as an internal standard and utilized CDCl_3_ and DMSO-d_6_ as solvents. The reported values for shifts (δ) and coupling constants (J) were expressed in units of parts per million (ppm) and Hz, respectively. High-resolution Biosystems Q-Star Elite time-of-flight electrospray mass spectrometry confirmed the chemical composition of the compounds. The purity of the synthesized compounds was tested by HPLC (Gilson, USA) with a C18 analytical column and a Thermo Separation Spectra SERIES UV100 detector (S2 Fig). The mobile phase was prepared from acetonitrile and water. All compound preparations showed a purity >96%. Compounds were dissolved at 100 mM dilution in DMSO and stored at- 20°C. For biological assays, a no-drug control at the highest DMSO concentration used in the respective experiment was carried out to rule out effects of the solvent.

### General procedure

#### Synthesis of novel HEA compounds *(*LTC-1026, 1027, 1028, 1029, 1031, 1032, 1034, 1041, 1042, and 1043*)*

Tert-butyl ((S)-1-((R)-oxiran-2-yl)-2-phenylethyl)carbamate (epoxide) (1.9 mmol), anilines (1.9 mmol), nitromethane (5 mL) were taken in a round-bottomed flask and microwave heated to 80°C (300 W) for 20 minutes. Afterwards, nitromethane was removed on a rotary evaporator, and the obtained material was recrystallized from ethyl acetate and hexane in 1:9 ratio to isolate BOC-protected intermediates. In the next step, deprotection was carried out by treating the BOC-protected intermediates with 15% trifluoroacetic acid (TFA) solution in dichloromethane (1.5 mL of TFA, dissolved in 10 mL of dichloromethane) at room temperature for 3–4 hrs. Excess TFA present in the reaction flask was washed with dichloromethane and removed under reduced pressure. The obtained crude product was extracted from ethyl acetate (15 mL x 3) and alkaline water (1N KOH) at a pH range of 8–9. The obtained ethyl acetate layer was removed on a rotary evaporator that afforded the listed compounds (*i*.*e*., LTC 1026, 1027, 1028, 1029, 1031, 1032, 1034, 1041, 1042, and 1043). The chemical composition of all newly synthesized compounds was confirmed by NMR (^1^H & ^13^C) and mass spectrometry.

#### Synthesis of PHT and LTC compounds

Synthesis of known compounds was accomplished by following our published synthesis procedures for PHT [14–16] and LTC compounds [12,17,18].

### Spectroscopic data for novel compounds

**(2S,3S)-3-Amino-1-((4-fluorophenyl)amino)-4-phenylbutan-2-ol (LTC-1026):** White solid; Yield, 74%; mp: 88–90°C; ^13^C NMR (101 MHz, CDCl_3_) *δ* 156.1 (d, J = 235.1 Hz, 1C), 145.9, 144.9, 138.7 (d, J = 31.9 Hz, 1C), 129.3, 128.8, 126.6, 115.7 (d, J = 22.3 Hz, 1C), 114.2, 71.7, 70.7, 57.5, 55.1, 48.7, 41.4; ^1^H NMR (400 MHz, CDCl_3_) *δ* 7.38–7.22 (m, 5H), 6.93–6.82 (m, 2H), 6.75–6.52 (m, 2H), 3.70–3.59 (m, 1H), 3.49–3.36 (m, 1H), 3.26 (dd, J = 12.0, 3.5 Hz, 1H), 3.13–3.05 (m, 1H), 2.97 (dd, J = 9.5, 4.4 Hz, 1H), 2.90 (dd, J = 15.8, 4.3 Hz, 1H), 2.57 (m, 2H).

**(2S,3S)-3-Amino-4-phenyl-1-((4-(trifluoromethyl)phenyl)amino)butan-2-ol (LTC-1027):** White solid; Yield, 71%; mp: 105–107°C; ^13^C NMR (101 MHz, CDCl_3_) *δ* 151.0, 138.3, 129.3, 128.9, 126.8, 119.2, 118.9, 112.3, 77.4, 77.1, 76.8, 71.6, 54.9, 47.3, 41.5, 29.8; ^1^H NMR (400 MHz, CDCl_3_) *δ* 7.40–7.16 (m, 7H), 6.62 (d, J = 8.3 Hz, 2H), 4.64 (s, 1H), 3.64 (d, J = 3.3 Hz, 1H), 3.33 (d, J = 12.0 Hz, 1H), 3.20–3.10 (m, 1H), 3.08–2.99 (m, 1H), 2.95 (d, J = 13.5 Hz, 1H), 2.58–2.52 (m, 1H).

**(2S,3S)-3-Amino-4-phenyl-1-((4-(trifluoromethoxy)phenyl)amino)butan-2-ol (LTC-1028):** White solid; Yield, 68%; mp: 112–114°C; ^13^C NMR (101 MHz, CDCl_3_) *δ* 148.6, 128.7, 128.1, 127.0, 126.7, 75.8, 24.0, 21.2; ^1^H NMR (400 MHz, CDCl_3_) *δ* 7.36–7.12 (m, 5H), 7.02 (d, J = 8.4 Hz, 2H), 6.58 (d, J = 9.0 Hz, 2H), 3.70–3.57 (m, 1H), 3.28 (dd, J = 12.2, 3.3 Hz, 1H), 3.18–2.99 (m, 2H), 2.94 (dd, J = 13.4, 4.6 Hz, 1H), 2.56 (dd, J = 13.4, 9.4 Hz, 1H).

**(2S,3S)-3-Amino-4-phenyl-1-((3-(trifluoromethyl)phenyl)amino)butan-2-ol (LTC-1029):** White solid; Yield, 70%; mp:72–74°C; ^13^C NMR (101 MHz, CDCl_3_) *δ* 148.7, 138.2, 129.7, 129.3, 128.9, 126.8, 116.5, 114.1, 109.2, 77.4, 77.1, 76.8, 71.4, 55.0, 47.6, 41.2, 29.8; ^1^H NMR (400 MHz, CDCl_3_) *δ* 7.37–7.08 (m, 6H), 6.93 (d, J = 7.5 Hz, 1H), 6.86–6.66 (m, 2H), 3.66 (s, 1H), 3.31 (dd, J = 12.1, 1.7 Hz, 1H), 3.21–3.00 (m, 2H), 2.95 (dd, J = 13.6, 4.5 Hz, 1H), 2.58 (dd, J = 13.3, 9.5 Hz, 1H), 2.21 (s, 2H).

**1-(4-(((2S,3S)-3-Amino-2-hydroxy-4-phenylbutyl)amino)phenyl)ethan-1-one (LTC-1031):** White solid; Yield, 69%; mp: 123–125°C; ^13^C NMR (101 MHz, CDCl_3_) *δ* 196.6, 152.5, 138.3, 130.9, 129.3, 128.9, 126.8, 111.8, 71.5, 54.9, 47.1, 41.4, 26.1; ^1^H NMR (400 MHz, CDCl_3_) *δ* 7.80 (d, J = 8.6 Hz, 2H), 7.24 (m, 5H), 6.57 (d, J = 8.7 Hz, 2H), 4.88 (s, 1H), 3.64 (dd, J = 7.1, 3.7 Hz, 1H), 3.37 (dd, J = 12.4, 3.1 Hz, 1H), 3.20 (dd, J = 12.4, 7.5 Hz, 1H), 3.08–2.99 (m, 1H), 2.94 (dd, J = 13.5, 4.6 Hz, 1H), 2.56 (m, 1H), 2.50 (s, 3H).

**(2S,3S)-3-Amino-1-((2,4-difluorophenyl)amino)-4-phenylbutan-2-ol (LTC-1032):** White solid; Yield, 66%; mp:89–91°C; ^13^C NMR (101 MHz, CDCl_3_) δ 157.2 (d, J = 7.6 Hz, 1C), 155.0 (dd, J = 35.3, 6.7 Hz, 1C), 131.1 (dd, J = 27.2, 4.2 Hz, 1C), 119.5, 114.3 (dd, J = 26.7, 3.7 Hz, 1C), 105.2, 72.0, 54.8, 48.3, 39.3; ^1^H NMR (400 MHz, CDCl_3_) *δ* 7.38–7.02 (m, 7H), 6.86–6.67 (m, 1H), 3.57 (dt, J = 8.1, 3.9 Hz, 1H), 3.31 (dd, J = 13.7, 4.0 Hz, 1H), 3.26–3.12 (m, 1H), 2.98–2.88 (m, 1H), 2.83 (dd, J = 13.5, 4.6 Hz, 1H), 2.54 (dd, J = 13.4, 9.5 Hz, 1H), 2.03 (s, 2H).

**(2S,3S)-3-Amino-1-((4-(*tert*-butyl)phenyl)amino)-4-phenylbutan-2-ol (LTC-1034):** White solid; Yield, 74%; mp:74–76°C; ^13^C NMR (101 MHz, CDCl_3_) *δ* 152.8, 134.4, 132.8, 129.2, 127.1, 122.0, 55.3, 34.8, 31.1; ^1^H NMR (400 MHz, CDCl_3_) *δ* 7.35–7.03 (m, 9H), 4.26 (s, 1H), 3.70–3.18 (m, 2H), 3.17–2.65 (m, 3H), 1.24 (s, 9H).

**(2S,3S)-3-Amino-1-((2,6-dimethylphenyl)amino)-4-phenylbutan-2-ol (LTC-1041):** White solid; Yield, 68%; mp: 63–65°C; ^13^C NMR (101 MHz, CDCl_3_) *δ* 146.0, 138.7, 130.1, 129.4, 128.9, 126.7, 122.3, 72.5, 55.5, 51.9, 41.4, 18.6; ^1^H NMR (400 MHz, CDCl_3_) *δ* 7.32 (t, J = 7.3 Hz, 2H), 7.27–7.21 (m, 1H), 7.17 (d, J = 6.9 Hz, 2H), 7.01 (d, J = 7.4 Hz, 2H), 6.89–6.82 (m, 1H), 3.61 (ddd, J = 6.9, 4.7, 3.7 Hz, 1H), 3.19 (dd, J = 12.3, 3.5 Hz, 1H), 3.04 (dt, J = 12.1, 6.6 Hz, 2H), 2.96 (dd, J = 13.4, 4.5 Hz, 1H), 2.55 (dd, J = 13.4, 9.5 Hz, 2H), 2.33 (s, 6H).

**(2S,3S)-3-Amino-1-((4-methoxyphenyl)amino)-4-phenylbutan-2-ol (LTC-1042):** White solid; Yield, 75%; mp:78–80°C; ^13^C NMR (101 MHz, CDCl_3_) *δ* 158.5, 152.8, 141.2, 135.7, 129.1, 127.5, 114.7, 80.1, 56.7, 55.8, 47.9, 41.6, 40.9, 29.8; ^1^H NMR (400 MHz, CDCl_3_) *δ* 7.36–7.29 (m, 3H), 7.16 (d, J = 8.1 Hz, 2H), 6.73 (d, J = 9.0 Hz, 2H), 6.41 (d, J = 9.0 Hz, 2H), 4.51–4.42 (m, 1H), 3.85 (dd, J = 13.1, 6.6 Hz, 1H), 3.73 (s, 3H), 3.16–3.09 (m, 2H), 2.95 (dd, J = 13.0, 6.5 Hz, 1H), 2.81 (dd, J = 13.4, 7.2 Hz, 1H), 2.61 (s, 2H).

**(2S,3S)-3-Amino-4-phenyl-1-(p-tolylamino)butan-2-ol (LTC-1043):** White solid; Yield, 76%; mp: 57–59°C; ^13^C NMR (101 MHz, CDCl_3_) *δ* 145.5, 136.1, 133.0, 129.9, 129.6, 129.3, 128.5, 128.0, 127.4, 114.1, 69.7, 55.7, 52.2, 48.1, 37.7, 20.5; ^1^H NMR (400 MHz, CDCl_3_) *δ* 7.25–7.16 (m, 3H), 7.10 (d, J = 7.0 Hz, 2H), 6.93 (d, J = 8.1 Hz, 2H), 6.48 (d, J = 8.3 Hz, 2H), 3.79 (d, J = 4.2 Hz, 1H), 3.34–3.20 (m, 1H), 3.17–3.01 (m, 2H), 2.97–2.86 (m, 1H), 2.79–2.67 (m, 1H), 2.21 (s, 3H).

### Parasites, mammalian cells and animals

*L*. *major* LV561(MHOM/IL/67/LRC-L137 JERICHO II) was passaged in M199 medium supplemented with 10% FCS, 1% BME vitamins, 2% urine and 0.1% amikin. The infectivity of the parasites was boosted by passaging in *Balb/c* mice and culturing the infected lymph nodes after six weeks. *L*. *infantum* (MHOM / BR / 76 / M4192) parasite was passaged in the same media formulation with 0.5% urine.

*L*. *major* LV561(MHOM/IL/67/LRC-L137 JERICHO II) amastigotes were passaged in Grace insect medium supplemented with NaHCO_3_, 0.1% amikin and 20% of FCS (pH: 5.4) and kept at 33°C.

*L*. *major* promastigote antigen was prepared by freeze-thawed (ten cycles of freezing in liquid nitrogen and thawing in a 37°C water bath of a 10^8^ cells per ml parasite culture). Total protein concentration was determined by the BCA assay kit (Sigma) according to the manufacturer’s instructions.

*T*. *brucei brucei* (strain 427 Lister) blood stream form (BSF) parasites were passaged in complete HMI-9 media supplemented with 10% FCS, Pen-Strep (0.1%) and 2-mercaptoethanol (0.014 v/v) and kept in a 37° C CO_2_ incubator in filter-cap flasks.

*T*. *cruzi* (Y strain) trypomastigotes were passaged in liver infusion broth media supplemented with tryptose, NaCl, Na_2_HPO_4_, KCl, glucose, hemin, Pen-Strep (1%), 10% FCS (pH: 7.2) and kept at 28°C.

*Entamoeba histolytica* (strain B2-5) was passaged in TY-1 medium (Trypticase peptone BBL, Yeast extract BBL, glucose, NaCl, K_2_HPO_4_, KH_2_PO_4_, cysteine, ascorbic acid, ammonium iron citrate, adult bovine serum, Diamond tween 80, penicillin G, amikacin (pH:6.8)) and kept in 37°C incubator in completely filled tubes with closed lid [19].

Mammalian cell lines (J774 and HepG2) were passaged in RPMI media supplemented with MEM, Pen-Strep (1%) and 10% FCS. Cultures were kept at 37°C incubator with 5% CO2 in filter-cap flasks.

Female *Balb/c* mice, aged 6–8 weeks, were purchased from the breeding stock in Czech Republic (Animalab, Czech Republic). Animals were kept under standard conditions according to European ethical guidelines for experimental animals.

### Phenotypic screens

All phenotypic screens and dose-response analyses were carried out at least in biological triplicate.

### Phenotypic screen for *Leishmania* promastigotes and axenic amastigotes

Exponential growth phase promastigotes of *L*. *major* or *L*. *infantum* and axenic amastigotes (*L*. *major*) were harvested and seeded in 384 well plates (white, non-transparent, tissue culture treated, flat bottom; Corning) (0.5x10^5^ cells per well). Compounds were pre-dispensed into the plate with an Echo 650 acoustic dispenser (Beckman Coulter). After 72h incubation, resazurin was added (1:10 v/v) from a 500 mM stock in PBS, followed by incubation for six hours. Then, fluorescence intensity was measured using a microplate reader (Synergy H1, BioTek) with excitation filter: 550 nm and emission filter: 590 nm. Efficient compounds were selected for the next round of viability assay with 2.5 μM concentrations of the compounds. Ultimately, compounds showing promising effects at lower concentration were selected for dose-response analyses, exposing promastigotes to 18 different concentrations of the compounds of interest. All experiments were performed with AmB as a positive control drug in parallel.

### Phenotypic screen for *T*. *brucei* and *T*. *cruzi*

Viability of *T*. *brucei brucei* BSF and *T*. *cruzi* trypomastigotes was assayed with fluorescence resazurin reduction assay, essentially as described above. Briefly, 1.25x10^4^ cell/ml of parasites were exposed to 25 μM of the compounds in 384 well plates (white, non-transparent, tissue culture treated, flat bottom; Corning) for 72h, in triplicate, before resazurin addition. Next, selected compounds were tested at 2.5 μM concentration, and promising compounds subjected to 18 concentrations dose response analyses. AN3057 and AmB were used as a control drug in *T*. *brucei* and *T*. *cruzi*, respectively.

### Phenotypic screen for *E*. *histolytica*

Viability of *E*. *histolytica* was assayed via a luminescence assay based on ATP quantification in the live cells. Briefly, 2.5x10^4^ cell/ml were exposed to 25 μM of compounds in 96 well plates (non-transparent, tissue culture treated, flat bottom; SPL Life Sciences) in triplicate for 48h. Plates were incubated in sealed anaerobic bags (Oxoid AGS) together with an Anaerogen compact bag (Thermo Scientific) and anaerobic indicator (Thermo Scientific) at 37°C. Then, Cell titer glo reagent (Promega) was added to the reaction (1:5) according to manufacturers’ instruction. After 15 min of orbital shaking, followed by 10 min incubation at RT, ATP-bioluminescence was quantified at RT using a Synergy H1 plate reader (Synergy H1, BioTek). Metronidazole was included as control drug in all the assays for *E*. *histolytica*. Test compounds indicating significant growth reduction in the primary screen were subjected to potency determination. These secondary screens were essentially carried out as detailed above, but with a 9 to 16-point concentration range of the test compounds spanning 0.01–100 μM.

### Cytotoxicity assay

Murine macrophage cells (J774) and human hepatocyte carcinoma HepG2 cells were seeded at 0.5x10^5^ cell/well in 384 well plates in triplicate, exposed to serial dilution of compounds, dispensed using an Echo 650 acoustic dispenser (Labcyte). After incubation for 72 h at 37°C, 5% CO_2_, cell viability was determined using fluorescence resazurin reduction assay (λ_Ex_: 550 nm; λ_Em_: 590 nm).

### Parasite rescue assay

J774 Murine macrophage cells (J774) were seeded in RPMI supplemented with 10% FCS and PMA (50 μg/ml) at a density of 2.5 x 10^5^ cell/ml (in triplicate) and left to differentiate for 24h at 37°C, 5% CO_2_. Then, after washing the cells once with warm (37°C) serum free RPMI, stationary phase *L*. *infantum* promastigotes were added at a ratio of 10:1 (2.5x10^6^ cell/ml). After 24 h incubation, cells were washed five times with serum free RPMI and exposed to a serial dilution of test compounds, prepared using an acoustic dispenser (Echo 650, Labcyte), in RPMI (2% FCS). After two days, cells were washed thrice, and 20 μl of 0.05% SDS in RPMI was added upon removal of the supernatant. Upon incubation under orbital shaking for 30 seconds, cell lysis was stopped by addition of 180 μl M199 medium supplemented with 10% FCS. After incubation for 3 days at 26°C, parasite numbers were determined using a resazurin reduction assay [20]. Briefly, after addition of resazurin reagent (500 mM in PBS) at a ratio of 1:10 (v/v), plates were incubated for 14 h at 26°C. Then, fluorescence intensity of the samples was measured with a microplate reader (Synergy H1, BioTek) with λ_Ex_: 550 nm and λ_Em_: 590 nm. Percent of viability was calculated according to cell only and media controls.

### *T*. *brucei* RNAi library preparation, screening, data analysis and validation by individual RNAi

RNAi library screening was carried out as previously described [21] with plasmids and primers detailed in S1 Table. pRPaSce* was linearized by AscI digestion, then, precipitated and a total amount of 10 μg was transfected into *T*. *brucei* 427 BSF 2T1/T7 cells (Lonza nucleofector, X-001 setting). Positive clones were selected with hygromycin (2.5 μg/ml) and blasticidine (1 μg/ml). The RNAi plasmid library (pZJM-RNAi) was propagated in Electromax DH10B cells (Invitrogen) and purified using the Qiagen plasmid purification Maxi kit. After linearization with NotI, the plasmid library was transfected into 2T1/T7 Sce* clones (Lonza nucleofector, X-001 setting). Transfectants were selected with phleomycin (1 μg/ml) and blasticidin (1 μg/ml).

RITseq library cells were expanded to 2x10^7^ cells in 150 ml HMI-9 and induced with 1 μg/ml of tetracycline for 24 h prior to PHT-39 addition. PHT-39 was added to the cells at 1 x EC50 concentrations, and the number of viable cells was monitored by daily hemocytometer counting. Once a resistant population had emerged, cells were expanded to >1×10^8^ cells, harvested and genomic DNA isolated. The RNAi cassettes remaining in the PHT39-selected clones were amplified from genomic DNA using the LIB2 primer pair (S1 Table). PCR products were purified (PCR purification kit, Qiagen) and subjected to PCR-free library preparation and next generation sequencing (NGS) carried out by BGI Tech Solutions (Hong Kong, China) on a DNBSEQ-T7 sequencing platform (PE150). Raw reads were filtered, removing adaptor sequences, contamination and low-quality reads. Index files were prepared with BWA (Burrows-Wheeler Aligner) Linux based tools from the *T*. *brucei* TREU927 reference genome (release 62) from TriTrypDB [22]. In the next step data were aligned to the indexed genome using BWA. Samtools and IGV tools [23] were used for sorting and indexing of the aligned file. Finally, only RNAi-barcoded sequences were filtered and final counts generated using featurecounts [24]. The Artemis package was applied to visualize the read coverage [25]. Functional annotation from TriTrypDB [22] was added using Perseus [26]. Transcriptomics data have been deposited to the ArrayExpress repository [27] with the accession E-MTAB-13844.

Selected genes with the high reads were validated by individual RNAi targeting. Briefly, gene-specific RNAi fragments of 400–600 bp were amplified with PCR primers designed with RNAit tool [28] (S1 Table) and cloned into pRPa^iSL^ to generate stem-loop, ‘hairpin’ dsRNA to induce RNAi knockdown [29]. The AscI linearized plasmid was transfected into 2T1 cells and positive colonies were selected with hygromycin (2.5 μg/ml). Positive, puromycin sensitive clones were analysed by PHT-39 dose response analysis after 24 h tetracycline induction and compared to uninduced controls and parental lines.

### Proteomics analysis

*T*. *brucei* BSF Cells treated with 3.9 μM PHT-39 (1× EC50) were grown in parallel with nontreated cells in triplicate. 4 x 10^7^ cells were harvested by centrifugation (1000*g) after 48 h from cultures in logarithmic growth phase and washed thrice with PBS. Samples were subjected to lysis, tryptic digest and reductive alkylation using standard procedures. LC-MS/MS was performed by the OMICS Proteomics Facility at Charles University Biocev on an UltiMate 3000 RSLCnano System (Thermo Fisher Scientific) coupled to a Orbitrap Fusion mass spectrometer (Thermo Fisher Scientific). Mass spectra were analyzed by label-free quantification using MaxQuant version 2.1.0.0 [30] searching the *T*. *brucei brucei* 927 annotated protein database (release 57) from TriTrypDB [31]. Minimum peptide length was set at seven amino acids and false discovery rates of 0.01 were calculated at the levels of peptides, proteins, and modification sites based on the number of hits against the reversed sequence database. When the identified peptide sequence set of one protein contained the peptide set of another protein, these two proteins were assigned to the same protein group. *P*-values were calculated, applying *t*-test–based statistics using Perseus [26]. Proteomics data have been deposited to the ProteomeXchange Consortium via the PRIDE partner repository [32] with the data set identifier PXD043204.

### Analysis of *T*. *brucei* cell cycle anomalies upon PHT-39 treatment

*T*. *brucei* BSF cells treated with 3.9 μM PHT-39 (1× EC50) were grown in parallel with nontreated cells in triplicate. 10^6^ cells were harvested by centrifugation (1000*g) after 24 h from cultures in logarithmic growth phase and washed once with serum-free HMI-9. After fixation in 2% formaldehyde cell were applied to a glass slide and mounted with Vectashield Antifade mounting medium with 4′,6-diamidino2-phenylindole (DAPI) for fluorescence microscopy imaging on a Leica TCS SP8 WLL SMD-FLIM inverted confocal microscope. The number of nuclei (N) and kinetoplasts (K) per cell were then counted for 100 cells in each respective replicate (n = 3), allowing determination of cell cycle stage [33].

### Experimental challenge and treatment protocol

*L*. *major* metacyclic promastigotes were prepared from a five day stationary phase culture through Ficoll gradient centrifugation according to previous studies [34] with minor changes: Briefly, a 20% and 10% Ficoll gradient were prepared in PBS and M199 media, respectively and placed in a 14 ml falcon tube. Then, 1x10^9^ *L*. *major* promastigotes were loaded on top of gradient and centrifuged. Metacyclic parasites were collected between two layers. Four groups of mice (n = 6) were challenged by subcutaneous injection of 2x10^6^ stationary phase metacyclic promastigotes (50 μl in PBS) into the left hind footpad. Two weeks after infectious challenge, animals were treated by i.p or intralesional (s.c) PHT-39 injection (13.75 mg/kg body weight), daily or every other day, respectively, for ten days. AmB (8 mg/kg body weight) was applied as positive control drug (i.p injection for ten days) and 5% DMSO in distilled water was applied as negative control (s.c injection for ten days) (Table 1).

**Table 1:** Balb/c mice cutaneous leishmaniasis model treatment regimen.

Groups	Amounts/route of injection	Number of injections	Duration of treatment
**G1 (PHT-39)**	(13.75 mg/kg) / i.p	10 times	10 days
**G2 (PHT-39)**	(13.75 mg/kg) / s.c	5 times	10 days
**G3 (AmB)**	(8 mg/kg) / i.p	10 times	10 days
**G4 (DMSO)**	(5%) s.c	5 times	10 days

### Footpad swelling and body weight measurement

Every four days after the challenge, body weight was monitored and footpad swelling changes were measured (Footpad swelling = (height + width)/2) with a metric caliper (Kroeplin, IP65, D-series, external measuring gauge).

### Parasite burden measurement in lymph node

After the 10 days course of treatment all animals were sacrificed, and popliteal lymph nodes of each infected footpad were collected for parasite load determination. Genomic DNA was extracted from the lymph nodes (DNeasy Blood and Tissue kit, Qiagen). Equal concentration of samples (125 ng) was applied to quantify leishmania kDNA with primers RV1(forward: 5’-CTTTTCTGGTCCCGCGGGTAGG-3’) and RV2 (reverse: 5’-CCACCTG GCCTATTTTACACCA-3’). qPCR data was normalized using the murine house-keeping gene GAPDH amplified with primers (forward:5’CGTCCCGTAGACAAAATGGT3’; reverse: 5’TTGATGGCAACAATCTTCAC3’) in parallel using Maxima SYBR green qPCR master (Thermo). qPCR was performed using Biorad CFX96 device with 95°C/ 10 min (initial denaturation), then 95°C/15s (denaturation), 60°C /30s (annealing) and 72°C/ 40s (extension) over 40 cycles. For ΔCT calculation of samples, every CT were subtracted from the same on GAPDH. Then, ΔΔCT of each group was normalized according to negative control group (DMSO).

### Cytokine and nitric oxide measurement

After 10 days of experimental treatment, all animals were sacrificed, and spleens were excised and ground with a plastic homogenizer under sterile conditions. Red blood cells were lysed with RBC lysis buffer (Invitrogen) followed by several washing steps in PBS. Cells were cultured (5 x 10^6^ cell/ml) in RPMI medium supplemented with 10% heat inactivated FCS, Pen-Strep (1/100) and MEM (1/100), either with freeze-thawed *L*. *major* cells (20 μg/ml) or Con A (5 μg/ml) as positive control and media only as a negative control in a 37°C, 5% CO_2_ incubator. The cell supernatants were collected on days three and five post experiment for measurement of IL-4 and IFN-γ, respectively. Cytokine measurements were performed with sandwich Elisa kits (R and D systems). Briefly, high binding ELISA plates (R and D systems) were coated with capture antibody overnight. The day after, the plates were blocked with blocking buffer (1% BSA in PBS or TBS) for 1 hour after washing out the capture antibody, then cell supernatant or serial dilutions of standard were added to the plate for two hours. After several washing steps, the detection antibody was applied for another two hours. Finally, after addition of streptavidin, and washing steps, substrate (R&D systems) was added to generate a color forming reaction. The absorbance was measured in a microplate reader (Synergy H1, Biotech) at λ = 450 nm and at a correction wavelength of 540 nm. Cytokine concentrations were calculated using a standard curve with known concentrations of related cytokines.

The amount of nitric oxide production in the supernatant of spleen cells was measured by Greiss assay. In this regard equal volumes of spleen cell supernatant were mixed with Griess reagent [0.1 N (1-naphthyl) ethylenediamine dihydrochloride (Sigma) and 1% sulphonyl amide (Sigma) in 5% H_3_PO_4_ (Sigma)] in a 96 well plate. The absorbance at λ = 550 nm was measured in microplate reader (Synergy H1, BioTek). The concentration of nitrite was calculated according to a standard curve generated from sodium nitrite serial dilution.

### Statistical analysis

All graphs were drawn, and statistical analysis performed with Graph pad prism 6. A student *t*-test was applied for parametric analysis and a Mann-Whitney test for non-parametric data with *p*-value < 0.05.

## Results

### Chemical synthesis, library generation

**Fig 1:**
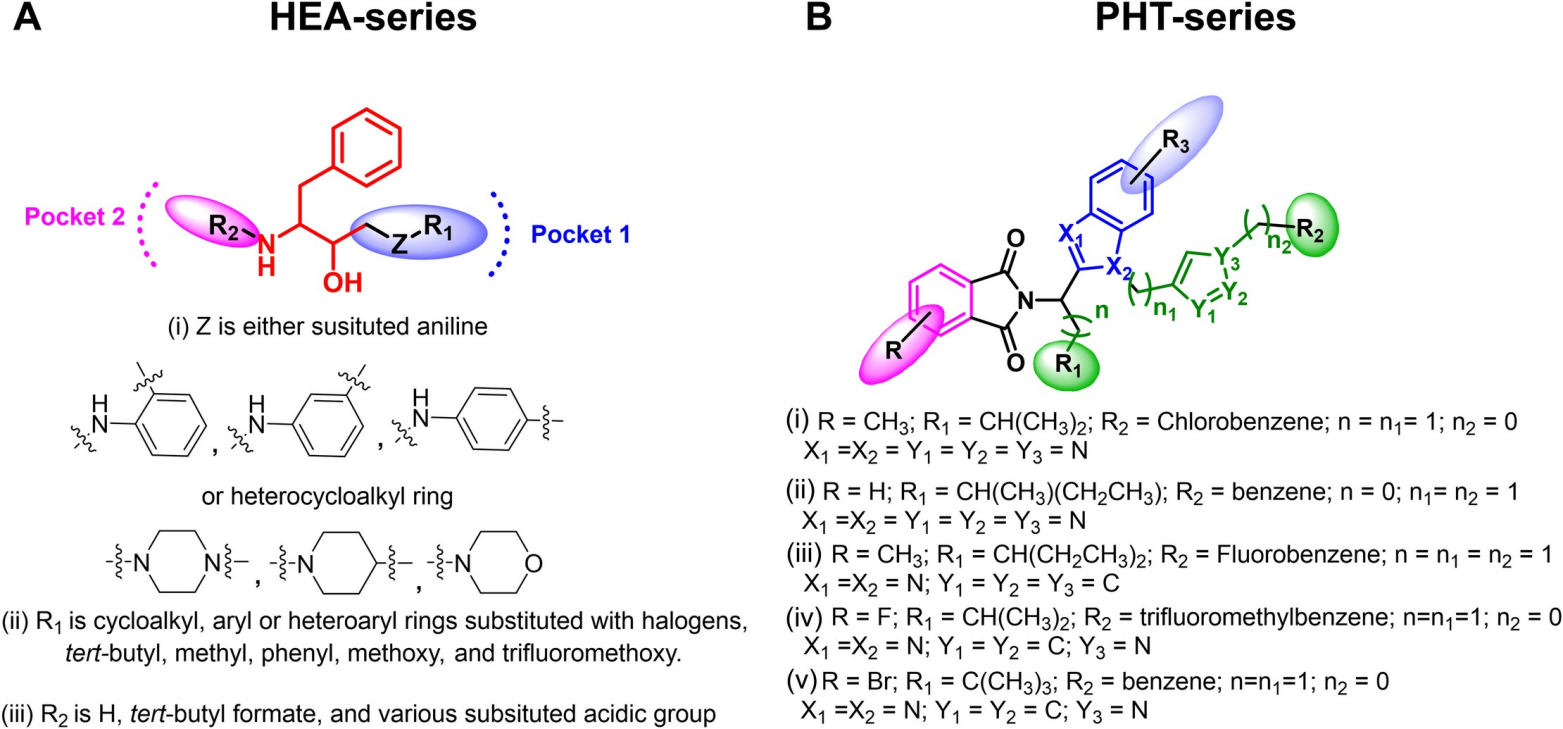
Chemical structures of HEA- and PHT-library compounds.

Previously, we reported the synergistic combination of PHT, benzimidazole, and triazole scaffolds leading to the discovery of novel antimalarial agents with 50% inhibitory concentrations (EC_50_) in the sub-micromolar range [15]. Subsequent hit-to-lead optimization resulted in a novel PHT derivative that showed efficacy in *Plasmodium berghei* infection model [16]. Likewise, we recognized HEA as a potential scaffold for the construction of novel antimalarial agents [13,35,36]. Various HEA derivatives were synthesized by introducing variations of Z-R_1_ and R_2_ groups, as depicted in Fig 1. The Z-R_1_ substitutions were amine groups that possess significant medicinal value, including piperazine [37], which serves as the primary core of several FDA-approved drugs [38,39], substituted aniline [40] and cyclohexyl ethyl [41], whereas the R_2_ position was substituted with different alkyl and aryl groups.

**Fig 2:**
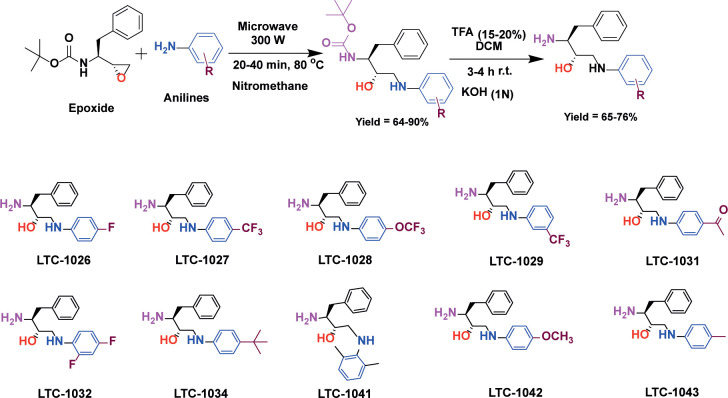
Synthetic pathway for HEA based novel compounds (LTC-1026, 1027, 1028, 1029, 1031, 1032, 1034, 1041, 1042, and 1043).

We set out to investigate the broad-spectrum antiparasitic efficacy of the reported and novel analogues based on HEA and PHT pharmacophores. In this manuscript, we report 10 novel and 56 known HEA compounds and 36 known analogs of PHT [15]. The novel HEA compounds *i*.*e*., LTC-1026, 1027, 1028, 1029, 1031, 1032, 1034, 1041, 1042, and 1043 were synthesized as depicted in Fig 2. Eleven published HEA compounds *i*.*e*., LTC-1001, 1002, 1004, 1016, 1017, 1023, 1024, 1025, 1035, 1036, and 1037 were resynthesized and optimization of the synthesis procedure led to significantly improved yields compared to those previously reported.

### Phenotypic screening

All the listed HEA and PHT compounds were employed for phenotypic screening against a diverse set of protist parasites, including *T*. *brucei*, *T*. *cruzi*, *Leishmania* spp., and *Entamoeba histolytica*. We carried out primary screens at two concentrations (25 and 2.5 micromolar) in triplicate cell viability assays. Hits for each respective target organism were then validated by dose response analysis.

### Compound screens against *Leishmania* promastigotes and axenic amastigotes *in vitro*

Screening the HEA library (at 25 μM) against *L*. *major* promastigotes showed that from 66 library compounds, 28 compounds reduced viability to less than 10%. A screen at lower concentration (2.5 μM) identified four compounds with potent activity against *L*. *major* promastigotes (LTC-37: 2.2% ± 1.6, LTC-541: 3.64% ± 1.87, LTC-555: 2.8% ± 1.1, LTC-572: 5% ± 0.54) (S2 Table and S3A and S3B Fig). The respective screen in *L*. *infantum* promastigotes, identified 35 compounds with lower than 10% viability at 25 μM concentration, while at 2.5 μM only LTC-37 showed potent activity (S3C and S3D Fig). Six hits were validated by dose response analysis in *L*. *major* promastigotes (S3 Table and S4A Fig). Screening the 36 compound PHT library (25 μM) against *L*. *major* promastigotes identified two compounds reducing viability to less than 10% (PHT-35: 6.3% ± 2.4, PHT-38: 5.4% ± 1.4) (S2 Table and S3A Fig). As the remaining 34 compounds had considerably less activity on promastigote growth, we skipped the screen at a lower concentration and subjected four hit compounds to dose response analysis giving EC50 values of 7.1 ± 1.7 μM (PHT-35), 6.3 ± 1.7 μM (PHT-38), 5.9 ± 1.8 μM (PHT-39) and 5.8 ± 1.7 μM (PHT-64) (Tables 2 and S3 and S4A Fig).

**Table 2:** Dose response analysis of selected hits. EC50 values (± standard deviation) in μM from triplicate experiments.

Compound	*T*. *brucei* BSF	*L*. *major* promastigote	*L*. *major* axenic amastigote	*L*. *infantum* rescue assay	HepG2	J774 macrophage
**PHT-39**	3.9 ± 1.7	5.9 ± 1.8	2.6 ± 1.8	1.2 ± 3.2	>100	>16
**LTC-1023**	1.1 ± 1.7	ND	ND	4.1 ± 1.8	31.6 ± 1.7	>25
**LTC-1025**	3.7 ± 1.7	ND	ND	2.8 ± 2.9	33.8 ± 1.7	>25
**LTC-1035**	2.9 ± 1.7	ND	ND	6.7 ± 1.7	16.6 ± 1.9	>25
**PHT-80**	0.5 ± 2.0	ND	2.5 ± 1.7	>10	ND	>25
**Control**	0.005 ± 0.002 (AN3057)	0.2 ± 1.7 (AmB)	0.3 ± 1.7 (AmB)	0.7 ± 1.9 (AmB)	ND	>10 (AmB)

**Fig 3:**
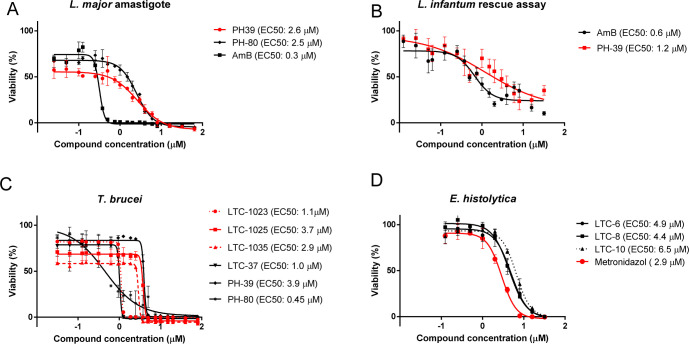
Dose response analyses of selected compounds for *L*. *major* axenic amastigotes (A), *L*. *infantum* intracellular amastigotes (B), *T*. *brucei* BSF (C) and *E*. *histolytica (*D). Derived EC50 values for each test compound are indicated in the legend (n = 3).

Axenic amastigotes, the relevant form that persists in the infected host, were generated in the lab through established procedures [42], employing harsh pH (pH 5) and high temperature (33°C) conditions. Applying the PHT-compounds at a concentration of 25 μM, 7 hits with residual viability of less than 6% were found for *L*. *major* axenic amastigotes: PHT-38 (1.0% ± 1.0), PHT-80 (1.7% ± 0.9), PHT-35 (1.9% ± 1.8), PHT-39 (2.6% ± 0.3), PHT-40 (3.4% ± 4.1), PHT-41 (4.6% ± 2.3), and PHT-36 (5.2% ± 1.6). However, when applying 2.5 μM concentration, all PHT compounds showed higher than 50% of residual viability (S2 Table and S3E Fig). For the HEA library the lowest observed viability at a concentration of 25 μM belonged to LTC-1034 with 15.2% ± 1.9 (S1 Table and S3F Fig). Significant hits were validated by dose response analysis (Tables 2 and S3), yielding the following EC50 values: PHT-35: 2.3 ± 1.9 μM, PHT-80: 2.5 ± 1.7 μM, PHT-39: 2.6 ± 1.8 μM and PHT-36: 3.0 ± 1.8 μM (Fig 3A).

### Compound screens against intracellular *L*. *infantum* amastigotes

In the disease context, compounds must exhibit the crucial ability to enter the host cells and their compartments efficiently in order to eliminate the intracellular parasite. Therefore, we next investigated the efficacy of eliminating intracellular amastigotes in a macrophage infection model. J774A.1 cells (J774) were infected with *L*. *infantum* metacyclic promastigotes. Infected cells were then treated with test compounds at a concentration of 25 μM and amphotericin B as a control. After controlled lysis of the infected macrophages, the released parasites were quantified using a resazurin based viability assay.

In this rescue assay, altogether 5 PHT-compounds and 7 HEA-compounds exhibited apparent low-micromolar potencies. PHT-39, with 16.3% ± 4.7 of residual viability was the most effective compound against the intracellular amastigote stage of *Leishmania* (PHT-36: 35.0% ± 2.5, PHT-55: 38.2% ± 2.2, PHT-60: 36.8% ± 7.4 and PHT-61: 35.7% ± 2.4) (S3H Fig). The respective HEA hit compounds (with less than 10% viability in rescue assay at 25 μM) were LTC-1115 (1.7% ± 0.1), LTC-551 (1.8% ± 0.3), LTC-1025 (2.8% ± 0.3), LTC-34 (2.8% ± 0.5), LTC-1023 (4.9% ± 2.5) and LTC-553 (6.9% ± 3.4) (S3I Fig).

In parallel, we assessed cytotoxicity towards J774 cells. While the great majority of PHT-compounds were found to be non-toxic up to ≥ 25 μM concentration (the highest cytotoxicity belonged to PHT-36 (46% ± 4) (S2 Table and S3J Fig), several HEA compounds had cytotoxicity issues (S2 Table and S3K Fig), and thus, were not selected for dose response analysis. The 3 remaining HEA-compounds (LTC-1023, LTC-1025 and-1035) and PHT-39, were selected for intramacrophage dose response analysis applying 18 concentration dilution series (Tables 2 and S3 and Fig 3). PHT-39 showed an EC50 of 1.2 ± 3.2 μM against *L*. *infantum*, while the HEA hits gave EC50 values within a range of 3–7 μM (Figs 3B and S4E).

### Cytotoxicity testing

While initial cytotoxicity testing in J774 indicated promising selectivity of the four lead compounds, we additionally determined dose response values for HepG2 cells. While the HEA compounds had CT50 values of 16.7 ± 1.9 μM (LTC-1023), 31.6 ± 1.7 μM (LTC-1025) and 33.8 ± 1.7 μM (LTC-1035) (Tables 2 and S3 and S4D Fig), our analyses showed no toxic effect for PHT-39 towards HepG2 cells for concentrations up to 100 μM. Given this promising selectivity, we earmarked PHT-39 for the mechanism of action studies and testing in a cutaneous leishmaniasis animal model.

### Compound screens against *T*. *brucei* BSF and *T*. *cruzi* epimastigotes

Blood stream form (BSF) *T*. *brucei* was overall the most sensitive target organism, with 70 compounds exhibiting apparent low-micromolar or nanomolar potencies (EC50 < 25 micromolar). The control compound AN3057, a highly potent trypanocidal phenoxy-benzoxaborole with a reported EC50 of 5.2 nM [43] inhibited *T*. *brucei* growth at 2.5 μM concentration with 0.3% of apparent residual viability. 27 PHT-compounds reduced *T*. *brucei* BSF viability to under 10% at 25 μM concentration, but only PHT-80 showed a dramatic effect on the cells with 2.5 μM concentration (3.4% of viability) (S2 Table and S3L Fig). Dose response analyses showed an EC50 of 0.4 ± 2.0 μM for PHT-80, and 3.9 ± 1.7 μM for PHT-39 (Figs 3C and S2B and Table 2). For 44 HEA compounds residual viability was lower than 10% at 25 μM and for 4 (LTC-37, LTC-1023, LTC-1029, LTC-1032) at 2.5 μM (S2 Table and S3M Fig).

Contrary, none of the library compounds showed promising effects against *T*. *cruzi* epimastigotes, even at high concentration (25 μM), while the respective control drug AmB lowered the viability to 7.4% (S2 Table and S3N and S3O Fig).

### Compound screens against *E*. *histolytica*

While the PHT library screen did not reveal compounds effective against *E*. *histolytica* (S3P Fig), 9 HEA series compounds were found active with apparent low micromolar potencies. The latter hits, when tested at 2.5 μM, showed only modest impact on viability (S2 Table and S3Q Fig). Nevertheless, dose response analyses were performed for 3 selected HEA-compounds, giving the following EC50-values (±SD): LTC-6: 4.9 ± 1.8 μM, LTC-8: 4.4 ± 1.7 μM, LTC-10: 6.5 ± 1.7 μM (Fig 3D and Tables 2 and S2).

### Analysis of PHT-39 sensitivity determinants by a forward genetics approach revealed potential uptake routes

**Fig 4:**
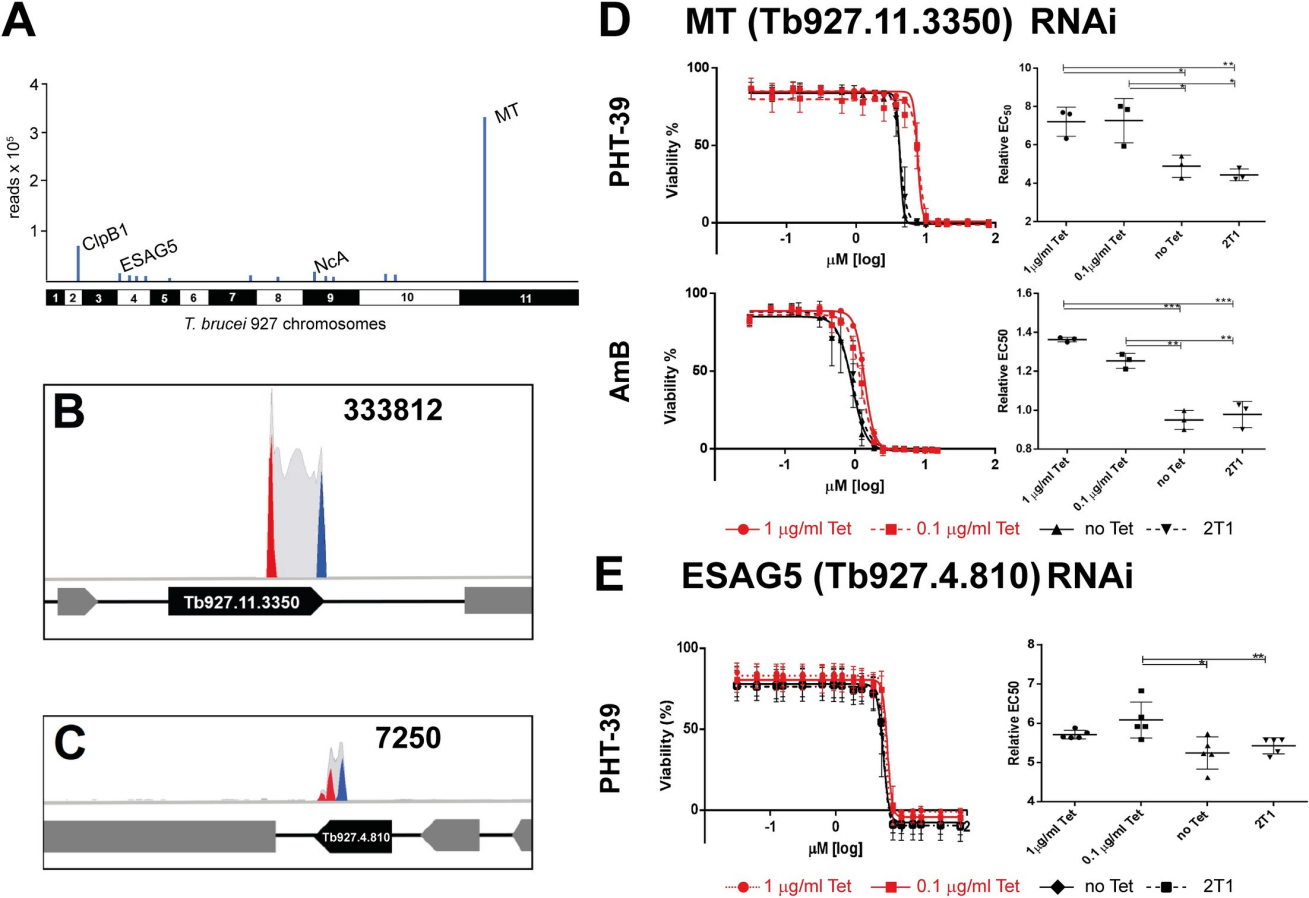
Identification of PHT-39 efficacy determinants in *T*. *brucei*. (A) Distribution of primary read-density signatures (>50 reads) across the *T*. *brucei* genome. Selected reads are labelled (MT = miltefosine transporter Tb927.11.3350; NcA nicotinamidase) (B) Reads mapping to the MT (Tb927.11.3350) locus and (C) ESAG5 (Tb927.4.810) locus. Red and blue peaks are RNAi construct forward and reverse barcodes, respectively. Gray peaks are all other reads. (D) EC50 analyses for PHT-39 (top) and amphotericin B (bottom) after individual knockdown of MT (Tb927.11.3350). EC50 analyses were carried out in 3 biological replicates. *P*-values were derived from unpaired Student’s *t*-test (*, *P* < 0.01; **, *P* < 0.001; ***, *P* < 0.0001). (E) EC50 analyses for PHT-39 (top) after individual knockdown of ESAG5. EC50 analyses were carried out in 3 biological replicates. *P*-values were derived from a non-parametric Mann Whitney test (*, *P* < 0.015; **, *P* < 0.0079).

To identify proteins which loss renders *T*. *brucei* less sensitive toward PHT-39 we applied a genome-wide RITseq screen [21]. The highest number of reads (approximately 11.5% of mapped reads (Fig 4A and 4B and S4 Table) was obtained for the RNAi construct-specific barcode corresponding to Tb927.11.3350, a predicted 127 kDa phospholipid transporting P-type ATPase. This 127 kDa integral membrane protein, with 10 predicted transmembrane segments, localizes to the endosomal compartment [44]. Intriguingly, this protein has been identified as a sensitivity determinant for the antileishmanial drugs amphotericin and miltefosine in *T*. *brucei* [45] and proposed as a respective uptake transporter in *Leishmania* [46,47]. Homologs are encoded syntenically in *Leishmania spp*. and the respective ortholog as for example, LmjF.13.1530 shares 70% sequence similarity (52% sequence identity).

The remaining candidate sensitivity determinants had considerably lower read coverage (Fig 4A and 4C and S4 Table). Among those, was the expression site associated glycoprotein 5 (ESAG5; Tb927.4.810), a glycosylphosphatidylinositol-anchored surface protein for which a function in lipid transfer/lipopolysaccharide-binding family was predicted based on domain conservation [48]. ESAG5 is one of the few ESAGs that are conserved in other kinetoplastids, including *Leishmania spp*. [49], which encode a syntenic ortholog of Tb927.4.810. Further RNAi targets associated with similar read counts were Tb927.2.5980, an HSP104 homolog belonging to the ClpB family of chaperones [50] and the two nicotinamidase paralogs Tb927.9.3970 and Tb927.9.4040. The latter enzymes play a role in prodrug activation of the antimycobacterial pyrazinamide that is converted into pyrazinoic acid [51], suggesting a potential role in PHT-39 activation.

In order to confirm PHT-39 sensitivity determinants revealed by the RITseq screen, we individually targeted four genes by stem-loop RNAi: Targeted RNAi of the miltefosine transporter Tb927.11.3350 recapitulated the sensitivity shift, eliciting a mild but significant EC50 increase, indeed to a similar degree as observed for amphotericin (Fig 4D). Individual knockdown of ESAG5 led to a very modest but significant sensitivity shift (Fig 4E), while RNAi targeting both nicotinamidase paralogs simultaneously, as well as the HSP104 homolog Tb927.2.5980, did not elicit a significant PHT-39 sensitivity shift (S5 Fig).

### Proteome changes upon PHT-39 exposure indicate mitochondrial activation and a potential impact on cytokinesis

**Fig 5:**
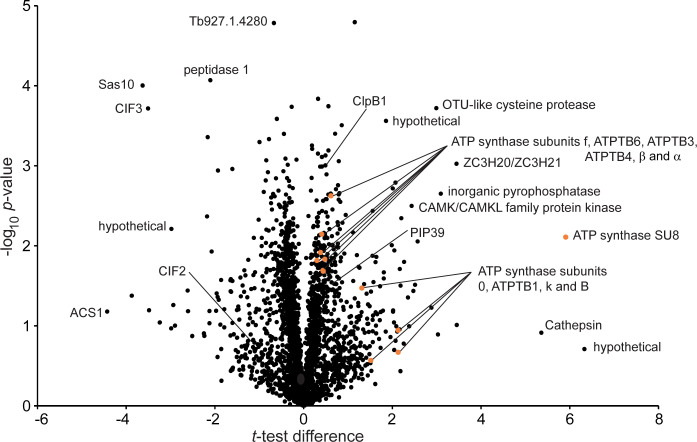
Global proteome changes upon 48 h of PHT-39 exposure. Shown is a volcano plot of the *t*-test difference plotted against the respective −log_10_-transformed P values. Selected data points are annotated, and mitochondrial F1Fo-ATPase components (GO: 0005753) highlighted in orange. For complete annotation and abundance ratios, see S4 (all data) and S5 Tables (protein groups associated with cytokinesis, SS-differentiation and mitochondrial metabolism).CIF, cytokinesis initiation factor; ACS1, Tb927.7.6180; Sas10, Sas10/Utp3/C1D family/Sas10 C-terminal domain containing protein Tb927.11.8050; ClpB1, Tb927.2.5980; PIP39, TbPTP1-interacting protein of 39 kDa, Tb927.9.6090.

We next used liquid chromatography coupled with tandem mass spectrometry (LC-MSMS) to quantify the effects of PHT-39 exposure on the global protein landscape in *T*. *brucei*. This label-free quantification experiment after 48 hours of treatment revealed a perturbed proteome with 2646 quantified protein groups (Fig 5 and S6 Table). Amongst the most significantly upregulated proteins is the zinc finger protein ZC3H20, which is indistinguishable from its paralog ZC3H21. ZC3H20/21 are mRNA binding proteins that have been reported to stabilise mRNAs associated with differentiation from the *T*. *brucei* long slender-bloodstream form (LS-BSF) to the procyclic form (PCF) colonizing the insect host, with maximal expression levels in the short stumpy BSF (SS-BSF) that is an intermediate stage in this differentiation process [52]. Consistently, TbPTP1-interacting protein PIP39 (Tb927.9.6090), a phosphatase that, upon phosphorylation, translocates into glycosomes and promotes the differentiation to SS-BSF [53], is 1.6-fold upregulated. Potentially linked to this differentiation response evoked by PHT-39 treatment is a GO-term enrichment for mitochondrial proton-transporting ATP synthase complex components (GO:0005753, enrichment factor = 6.7; *P* < 0.002) within the upregulated cohort. 15 out of 16 detected mitochondrial ATP synthase subunits showed increased abundance (S5 and S6 Tables). Likewise, the levels of multiple enzymes with prototypic functions in PCF mitochondrial metabolism showed elevated levels, as for example enzymes from the Krebs’ cycle and amino acid metabolism (S5 and S6 Tables). Further, PHT-39 treatment increased the abundance of the Hsp104 homolog Tb927.2.5980 (1.4-fold), which is also upregulated in SS-BSF [54], and was also detected in the RITseq screen (Fig 4A and S4 Table).

In the decreased cohort were several cytokinesis initiation factors (CIF 1–4, see S6 Table), which moderate cytokinesis, the final step of cell division, in *T*. *brucei* and localize to the flagellar attachment zone (FAZ) [55]. Further flagellar proteins were also decreased, as for example the FAZ localized protein Tb927.1.4280 (0.62-fold), the axonemal protein SAXO [56] (Tb927.8.6240; 0.65-fold) and ACS1 (Tb927.7.6180; exclusively detected in untreated cells), present at tips of old and new flagella [57]. The abundance changes of flagellar proteins with potential roles in cytokinesis prompted us to analyse cell cycle anomalies by microscopy. While there was no apparent increase in defective cytokinesis segregation, we observed a decreased proportion of cells possessing two kinetoplasts and, either one nucleus (2K1N decreased to approximately 57%) or two nuclei (2K2N; decreased to approximately 28%) in cells exposed to PHT-39 (at 1 x EC50 concentration) for 24 h (S6 Fig), indicating a block in cell cycle progression.

### Testing PHT-39 efficacy in a cutaneous leishmaniasis mouse model

The most significant requirement of an anti-*leishmanial* drug is its ability to reduce the parasite burden in the infected host. Hence, we tested the efficacy of PHT-39 in a cutaneous leishmaniasis mouse model. Briefly, four groups of 6–8 weeks Balb/c mice were challenged with 2 x 10^6^ *L*. *major* promastigotes subcutaneously injected into the hind footpad. Treatment started after two weeks when a wound started to form. Two groups were treated with PHT-39 compound at doses of 13.75 mg/kg either via i.p route, daily, or s.c route, every other day for 10 days. One group received a daily dosage of AmB as a control, and another infected non-treated group was included in the study. After 10 days, popliteal lymph nodes were collected to assess parasite burden and immune response stimulation was studied in spleen cell culture supernatants.

### Only Amphotericin B was able to reduce parasite load in lymph nodes of infected animals

**Fig 6:**
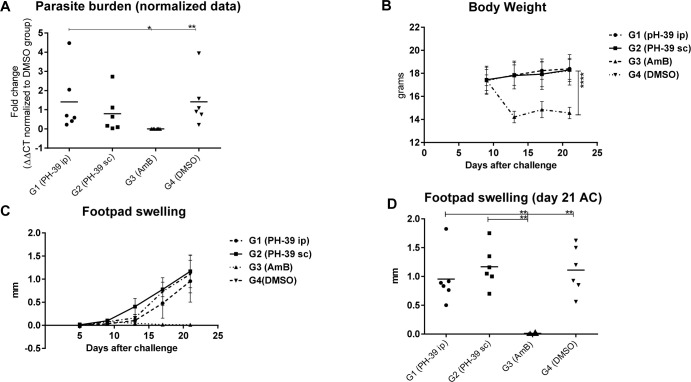
(A) Parasite burden determination in infected lymph nodes by qPCR. Data was normalized against GAPDH transcript levels quantified in the infected tissue in parallel with the parasite kDNA detection. (B) Body weight measurement during treatment course. (C) Footpad swelling measurement after challenge with *L*. *major* promastigotes. (D) Footpad swelling at the last day of treatment. Statistical analyses were performed by unpaired student *t*-test and Mann Whitney test *, **, **** = *P*-value < 0.05, 0.01 and 0.0001, respectively.

Parasite load was monitored by quantitative PCR (qPCR) of parasite DNA, extracted from lymph nodes of experimental animals. To this end, a specific conserved kDNA region was quantified using mouse GAPDH levels for normalization. The results showed that, AmB could reduce the parasite load significantly compared to the DMSO control group (group 4, *p*-value: 0.0079), while the test groups (s.c and i.p injection of PHT-39) did not show a significant difference. Further, parasite loads in AmB group were significantly lower than PHT-39 i.p (group 1, *p*-value: 0.0406) but it was not significantly different from the PHT-39 s.c (group 2) (Fig 6A).

### PHT-39 treatment failed to reduce footpad swelling

A further indicator of infection development in challenged murine footpads is the degree of swelling and extent of wound generation in the tissue. At day 21 post challenge, footpad swelling in AmB group was reduced significantly after treatment compared to the DMSO control (group 4), as well as to PHT-39 i.p (group 1), and PHT-39 s.c (group 2) (*p*-value: 0.0022). Neither of the test groups showed significant difference with the DMSO control (Fig 6C and 6D), which is in agreement with the parasite burden qPCR assay.

### Body weight in AmB control group reduced during treatment course

Our experiment indicated that application of PHT-39 in i.p or s.c routes did not affect the body weight in the test groups when compared to DMSO. Contrary, the AmB control group showed significant differences with other groups (*P*-value: 0.0001) due to toxicity of the drug (Fig 6B).

### Cytokine production profile in the experimental animals

**Fig 7:**
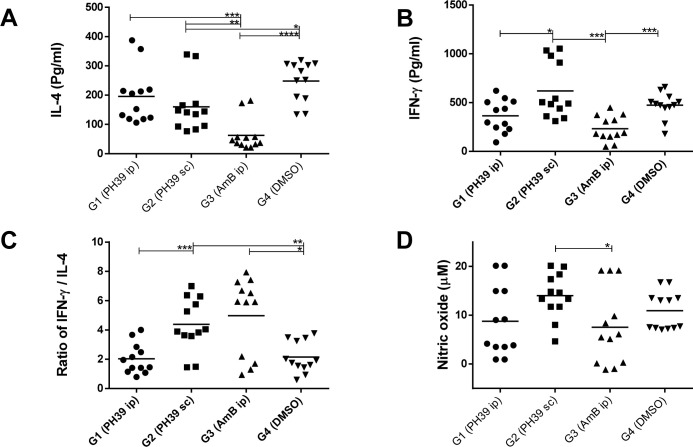
Cytokine and nitric oxide production in spleen cell supernatant of treated animals. (A) IL-4, (B) IFN-γ, (C) IFN-γ/ IL-4 ratio (D) Nitric oxide. Unpaired student *t*-test was used for nitric oxide, IFN- γ and IL-4. In ratio graph Mann Whitney test was applied where necessary. Asterisks indicate the significant difference between values in each group. The *, **, *** and **** = *P*-value < 0.05, 0.01, 0.001 and 0.0001 respectively. All tests were performed in duplicate and with 6 animals per group. Mean values are indicated by horizontal lines. For additional controls (no antigen and ConA) see S7 Fig.

To assess immunological effects of infectious challenge and treatment, production of IFN-γ and IL-4 were measured in cell supernatants of splenocytes. The healing process during the *Leishmania* infection is accompanied by higher levels of Th1 cytokines and lower Th2 response [58]. Hence, we assayed IFN-γ and IL-4 as major representatives of the Th1 and Th2 response, respectively. As *L*. *major* infection is systemic in *Balb/c* mice model [59], the spleen of every individual animal was collected and after several washing steps, cells were exposed to crude *L*. *major* antigen (freeze-thawed promastigotes) and the amount of cytokines was quantified in cell supernatants. Our results indicated that, IL-4 production in group 3 (AmB) was significantly lower compared to all other groups (group 3 vs group 4 *p*-value: 0.0001, group 3 vs group 2 *p*-value: 0.0034 and group 3 vs group1 *p*-value: 0.0003) (Fig 7A). In the case of IFN-γ, administration of AmB (group 3) suppressed the amount of IFN-γ significantly compared to DMSO control (*p*-value: 0.0002). IFN-γ production was higher in group 2 (PHT-39 s.c) compared to PHT-39 i.p group (*p*-value: 0.0134) and AmB (*p*-value: 0.0001) (Fig 7B).

Another important criterion to consider in infectious *Leishmania* studies, is the ratio of Th1/Th2 cytokines. This ratio in the AmB group was higher than in the DMSO control group (group 4, *p*-value: 0.0204) and PHT-39 i.p (group1, *p*-value: 0.0145), which is consistent with reduction of parasite burden and lowest foot pad swelling (Fig 7C). In our experiment PHT-39 i.p (group 1) did not show any significant difference with DMSO control. On the other hand, PHT-39 s.c (group 2) showed a significantly higher ratio than PHT-39 i.p (group1, *p*-value: 0.0007) and the DMSO control (group 4, *p*-value: 0.0013).

### Nitric oxide production in splenocytes

Next to Th1 cytokines, production of ROI and nitric oxide in infected cells reveals a healing process in *Leishmania* infected animals [58]. To test for nitric oxide levels, splenocytes of experimental animals were exposed to freeze-thawed whole antigen of *L*. *major* promastigotes for 5 days. Then, cell supernatant was subjected to a Griess reaction for quantification of nitrite production. According to the results, only group 2 (PHT-39, s.c) showed significantly higher amount of nitric oxide compared to the AmB control (group 3, *p*-value: 0.02). However, the experiment did not show any difference in other groups in favour of significant nitric oxide production (Fig 7D).

## Discussion

During the past few years, our studies have demonstrated HEA and PHT as important pharmacophores for the discovery of potent antiparasitic agents [13,36]. Here we assessed these compound libraries in a diverse set of protist parasites.

### Structure activity relationship

**Fig 8:**
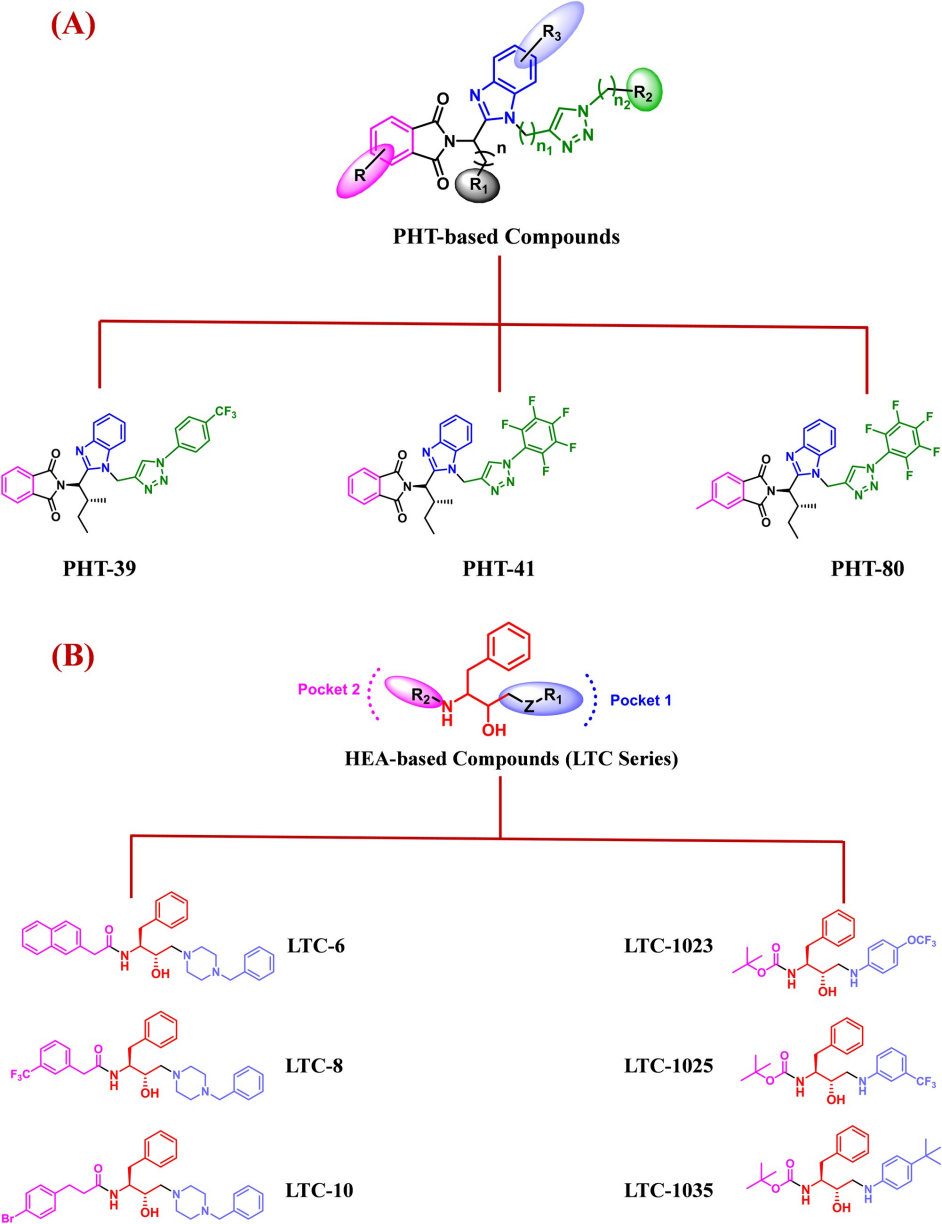
Structure-activity relationship (SAR) analysis of (A) PHT-based compounds (PHT-39, 41, 80); (B) HEA-based compounds (LTC-6, 8, 10, 1023, 1025, and 1035).

For PHT, a synergistic combination of phthalimide, benzimidazole and triazole scaffolds against malaria was previously reported, and several antiplasmodial hit compounds were obtained with inhibitory concentrations in the sub-micromolar range [15]. However, the therapeutic promise of those hits was compromised by toxicity towards the mammalian cells. Keeping these facts in view, respective derivatives were synthesized (S2 Table) in a hit to lead approach [16]. Replacement of a fluoro-group on the benzene ring fused to the triazole moiety (R2; compare Figs 1B and 8A) by trifluoromethyl (-CF_3_) substitution, a group known to improve activity by extending plasma half-life [60], generated the potent antiplasmodial lead compound PHT-39, that lacked any apparent toxicity towards human cells [16]. Likewise, PHT-39 excelled in our screen against intracellular *L*. *infantum* amastigotes. Similar to the antiplasmodial structure-activity relationship (SAR), structural modifications conducted on the PHT pharmacophore (Fig 1B) revealed that substituting R_2_ with either a benzene ring bearing trifluoromethyl substitutions or a fluorobenzene group, in conjunction with a branched alkyl substituent at the R_1_ position, resulted in enhanced antileishmanial activity. The compound denoted as PHT-39, distinguished by the presence of a branched alkyl substituent (R_1_) and a trifluoromethylbenzene moiety that is linked to the triazole (R_2_) was exhibiting the highest efficacy in the intramacrophage assay. Addition of a methyl group at the R position to the PHT moiety (Fig 1B) through derivatization enhances the potency towards *T*. *brucei*, as evidenced for compounds PHT-80 and PHT-41 (S2 Table and Fig 8A). However, we observed that this modification reduces anti-leishmanial activity (Fig 1B). Altogether, the antileishmanial SAR exhibited a remarkable resemblance to the antiplasmodial SAR with reference to the PHT library [13].

The HEA-library delivered three *Leishmania* intramacrophage hit compounds, namely LTC-1023, 1025, and 1035, which have substituted aniline at Z-R_1_ and *tert*-butyl formate at the R_2_ position (Figs 1A and 8B). LTC-1023, bearing an electron-withdrawing group ((EWG) -OCF_3_) located at the *para* position of aniline, exhibited an EC_50_ value of 4.1 ±1.8 μM. LTC-1035, which features an electron donating group ((EDG) -*tert*-butyl) at the aniline *para* position, exhibited an EC_50_ value of 6.7 ± 1.7 μM. The most effective HEA compound LTC-1025, with an EC_50_ value of 2.9 ± 2.9 μM, possesses a -CF_3_ group (EWG) at the aniline *meta* position. Overall, *meta*-substituted aniline exhibited greater efficacy in comparison to *para*-substituted aniline, and aniline scaffolds attached to EWGs demonstrated higher efficacy over those attached to EDGs.

The outcomes of the phenotypic screen for *E*. *histolytica* revealed that the identified hits did not exhibit any similarity with hits obtained from the kinetoplastida targets. PHT library compounds did not yield a significant effect, whereas the HEA-based compounds LTC-6, 8, and 10 exhibited robust activity. The latter compound trio share a benzyl piperazine moiety located at the -Z-R_1_ pocket (Figs 1A and 8B), while bearing distinct aromatic acids at the R_2_ position. LTC-6, which contains a 2-(naphthalen-2-yl) acetic acid moiety, exhibited an EC_50_ value of 4.9 ± 1.8 μM. On the other hand, LTC-8, which carries an additional–CF_3_ group on the R_2_ moiety (2-(7-(trifluoromethyl) naphthalen-2-yl) acetic acid moiety) of LTC-6, demonstrated slightly improved activity with an EC_50_ value of 4.4 ± 1.7 μM. The activity was reduced when the acidic moiety located at R_2_ was substituted with 3-(4-bromophenyl) propanoic acid, resulting in an EC_50_ value of 6.5 ± 1.7 μM. In general, the findings suggest that the substituted naphthalic acid exhibited greater efficacy in comparison to the substituted phenyl acidic group. Secondly, substitution with an EWG, such as -CF_3_, had a favourable effect on the activity.

### PHT-39 eliminates intracellular amastigotes with high selectivity

Antileishmanial drug discovery efforts are significantly hampered by the difficulty to reach the intracellular stage of the parasite which is engulfed by three membrane layers that need to be permeated by the drug molecule, which additionally is faced with extreme pH differences on its journey [61]. Hence, inhibitors identified from target-based approaches or phenotypic screening of promastigotes or axenic amastigotes often fail when tested in the intracellular context. The intramacrophage assay as a primary screen is cumbersome, and throughput is limited. However, notably, activity for a compound series detected in this assay frequently translates into activity in an animal infection model [62,63] apart from potential issues arising from its pharmacokinetics. Our intramacrophage screen identified altogether 4 compounds with low micromolar potencies (Table 2 and Figs 3B and S4E). Cytotoxicity testing of the 3 HEA compounds displayed a modest selectivity window, while PHT-39 exhibited >90-fold selectivity (S4D Fig and S3 Table) and was thus selected for mechanism of action studies and testing in a cutaneous leishmaniasis animal model.

### An orthology-based approach reveals a PHT-39 uptake route via the miltefosine transporter

Our genome-wide RITseq screen identified the endosomal P-type ATPase Tb927.11.3350 as a major contributor to PHT-39 efficacy in *T*. *brucei* (Fig 4). This phospholipid transporter with flippase activity has been previously described as miltefosine and amphotericin sensitivity determinant in *T*. *brucei*, [45] and the syntenic *Leishmania* ortholog is a well characterised miltefosine transporter. In *Leishmania donovani*, the transporter localizes to the plasma membrane and is functionally dependent on the presence of a potential additional subunit termed Ros3 [64], that was not detected as contributor to either PHT-39, or amphotericin/miltefosine efficacy in *T*. *brucei* [45]. Further, the endosomal localisation in *T*. *brucei* suggests that Tb927.11.3350 facilitates PHT-39 transport across the membrane of endosomal compartments, raising the question how the drug is internalised. A potential candidate receptor for endocytic uptake is ESAG5, detected in the RITseq screen, which has been proposed as lipid receptor [48]. Notably, ESAG5 has syntenic orthologs in *Leishmania spp*., for example LmjF.13.1530 sharing 39% sequence similarity (21% sequence identity) and has been found to be upregulated in the *Leishmania infantum* amastigote stage [65]. Targeted RNAi of ESAG5 in *T*. *brucei* elicited a significant, albeit extremely modest, PHT-39 sensitivity shift that may reflect a trade-off between sensitivity and fitness loss. Altogether, our data strongly suggest a shared uptake route for PHT-39 and the two frontline antileishmanials, miltefosine and amphotericin.

### Proteome changes induced by PHT-39 indicate mitochondrial activation and cell cycle arrest

PHT-39 treatment led to a SS-BSF like expression profile, associated with mitochondrial activation. Similar responses have been described for other trypanocidal drugs, including suramin [66] and melarsoprol [67], and have been coined to a drug induced stress response [68]. Moreover, the activation of metabolic pathways similar to the SS-BSF phenotype are in these cases likely arising from an attempt to generate ATP in the mitochondrion [66], for which in LS-BSF cells glycolysis remains the predominant source, although recent findings indicate greater bioenergetic flexibility [69]. Notably, we observed in our earlier work that PHT-39 exposure of *Plasmodium falciparum* trophozoites for 3 h (at the respective EC50 concentration of 0.64 μM) elicits a collapse of the mitochondrial membrane potential (MMP) [16]. Such rapid perturbation of the MMP was also observed for suramin treated *T*. *brucei* cells (after 2h), preceding a drop in cellular ATP levels (after 12h) and the onset of mitochondrial activation (between 24 and 48h) [66]. In conclusion, the PHT-39 induced mitochondrial activation likely constitutes a secondary response.

The decreased abundance of 2K1N and 2K2N cells upon PHT-39 treatment (S6 Fig) indicates a defect in cell cycle progression, which is consistent with the impact on cytokinesis initiation factors levels. It is tempting to speculate that these effects on the cell cycle are triggered by inhibition of tubulin polymerization, which has been proposed as a target for PHT-39 in *Plasmodium* [16]. However, the observed effect could be also attributed to the drug induced perturbation of energy homeostasis. Further studies are needed to identify the molecular target of PHT-39 for which we intend to employ a respective orthology-based genome-wide overexpression library that has recently become available [70].

### PHT-39 failed to eliminate *L*. *major* infection in a cutaneous leishmaniasis mouse model

Footpad swelling measurement showed that AmB control treatment effectively reduced the swelling in infected animals. However, PHT-39 treatment (i.p and s.c) did not show any significant changes compared to untreated control animals. Consistently, in qPCR assays, reduction of parasite load in the AmB group was significant compared to control, while it was not significantly changed in the PHT-39 groups. Nevertheless, PHT-39 administered s.c. lead to a lower but insignificant parasite burden and splenocytes isolated from this group induced more IFN-γ, as compared to i.p treated or AmB, indicating a moderate, albeit significant effect. In conclusion, PHT-39 treatment failed to eliminate the *Leishmania* infection in the cutaneous leishmaniasis animal model, while intraperitoneal or subcutaneous injection of PHT-39 for 10 and 5 days, did not expose any compound related toxicity. In contrast, PHT-39, as well as PHT-40, achieved a considerable decrease in parasitemia and significantly extended host survival when tested in a murine *Plasmodium berghei* ANKA infection model [16]. As these two compounds exhibit favorable ADME (absorption, distribution, metabolism, and excretion) properties and, significantly, have potencies against *Plasmodium falciparum* intraerythrocyte stages (PHT-39 EC50 = 0.6 μM; PHT-40 EC50 = 2.3 μM) [16] similar to PHT-39 in the intramacrophage assay (EC50 = 1.2 μM), it is tempting to speculate that therapeutic efficacy for cutaneous leishmaniasis is hampered by biodistribution, i.e., insufficient levels of the active agent accumulating within the infected lesion. Due to the absence of any effect in the cutaneous leishmaniasis model we did not subject PHT-39 to pharmacokinetic-pharmacodynamic analyses or further *in vivo* testing at this stage. However, we are currently trying to optimize PHT-39 by derivatization and will consider such analyses and a visceral leishmaniasis infection model in future.

## Supporting information

S1 Fig NMR data for LTC-1026,1027,1028,1029,1031,1032,1034,1041,1042 & 1043. (PDF)

S2 Fig HPLC data for PHT-39, PHT-40, PHT-51, PHT-77, PHT-79, LTC-1023 & LTC-1025. (PDF)

S3 Fig Primary HEA and PHT compound library screens for all target organisms, A) *L*. *major* promastigote response to PHT compounds (25 μM), B) *L*. *major* promastigote response to HEA compounds, C) *L*. *infantum* promastigote response to PHT compounds. D) *L*. *infantum* promastigote response to HEA compounds, E) *L*. *major* amastigote response to PHT compounds, F) *L*. *major* amastigote response to HEA compounds (25 μM), G) *L*. *major* amastigote response to HEA compounds (2.5 μM), H) *L*. *infantum* rescue assay for PHT compounds (25 μM), I) *L*. *infantum* rescue assay for HEA compounds (25 μM), J) Toxicity assay in J774 cells for PHT compounds, K) Toxicity assay in J774 for HEA compounds L) *T*. *brucei* BSF response in PHT compounds, M) *T*. *brucei* BSF response to HEA compounds, N) *T*. *cruzi* response to PHT compounds, O) *T*. *cruzi* response to HEA compounds, P) E. histolytica response to PHT compounds. Q) E. histolytica response to HEA compounds.
(PDF)

S4 Fig **Dose response analyses of hits from PHT and HEA compound screens** for *L*. *major* promastigotes (A), *T*. *brucei* BSF (B), *L*. *major* axenic amastigotes (C), cytotoxicity dose response analyses for HepG2 and J774 cells of selected HEA and PHT compounds (D) and the *L*. *infantum* rescue assay for selected HEA compounds (E).
(PDF)

S5 Fig **Dose response analyses for PHT-39 after targeted knockdown** of the two nicotinamidase paralogs Tb927.9.3970 and Tb927.9.4040 (A) and the ClpB1 chaperon Tb927.2.5980 (B), carried out in 3 biological replicates, respectively, did not determine a significant sensitivity shift.
(PDF)

S6 Fig Karyotype progression after 24h PHT-39 exposure compared to untreated cells (number of cells counted >100; in biological triplicate) employing DAPI-stained nuclear and mitochondrial DNA as cytological markers for cell cycle stages [71]: one nucleus and one kinetoplast (1N1K) indicate G1/S phase, one nucleus and two kinetoplasts (1N2K) indicate G2/M phase, and two nuclei and two kinetoplasts (2N2K) indicate postmitotic cells. One kinetoplast and two nuclei (2N1K) is an aberrant karyotype and not part of the canonical cell cycle.
(PDF)

S7 Fig Cytokine production in supernatant of spleen cells (as in Fig 6) with additional no-antigen (NoAg) control and ConA positive control for (A) IFN-γ, (B) IL-4. Graph shows Mean ± SD. Unpaired student *t-*test was used for IFN- γ and IL-4. Asterisk indicates the significant difference between values in each group. The *, **, *** and **** = P-value < 0.05, 0.01, 0.001 and 0.0001 respectively. All tests were performed in duplicate and 6 animals per group. F/T: Freeze-thawed whole antigen of *L*. *major* cells.
(PDF)

S1 Table Primers, plasmids and cell lines. (DOCX)

S2 Table Compound structures and growth inhibition observed in primary screens. Primary HEA and PHT compound library screens for all target organisms, the intramacrophage assay and J774 cytotoxicity. Residual viability (in %, comparing to non-treated cells, +/- standard deviation from triplicate experiments), is given for each test compound at concentration of 25 μM and 2.5μM, respectively. For *L*. *major* promastigotes and the intramacrophage assay only those compounds effective at 25 μM were tested at lower concentration. For corresponding bar graphs see S1 Fig.
(XLSX)

S3 Table Dose response analyses of hits from PHT and HEA compound screens and cytotoxicity dose response analyses for HepG2 and J774 cells. (XLSX)

S4 Table Genome-wide RITseq screen. (XLSX)

S5 Table Global proteome changes upon 48 h of PHT-39 exposure. (XLSX)

S6 Table Impact of PHT-39 exposure on the abundance on selected protein cohorts (stumpy- differentiation signalling pathway, F1Fo-ATPase, cytokinesis initiation factors, flagellar proteins with potential role in cytokinesis, mitochondrial metabolism, glycolysis). Abundance changes for each protein are compared with those observed upon 48h suramin treatment [66] and stage specific changes comparing BSF long slender with stumpy and PCF cells, respectively [54].
(DOCX)
