# Supplementary material for: Phenotypic screening reveals a highly selective phthalimide-based compound with antileishmanial activity
Source: PLoS Negl Trop Dis. 2024 Mar 25;18(3):e0012050. doi: 10.1371/journal.pntd.0012050 (PMC10994559; doi:10.1371/journal.pntd.0012050)
Supplement: S1 Fig — (PDF) [file pntd.0012050.s001.pdf]

S1 Fig. NMR data

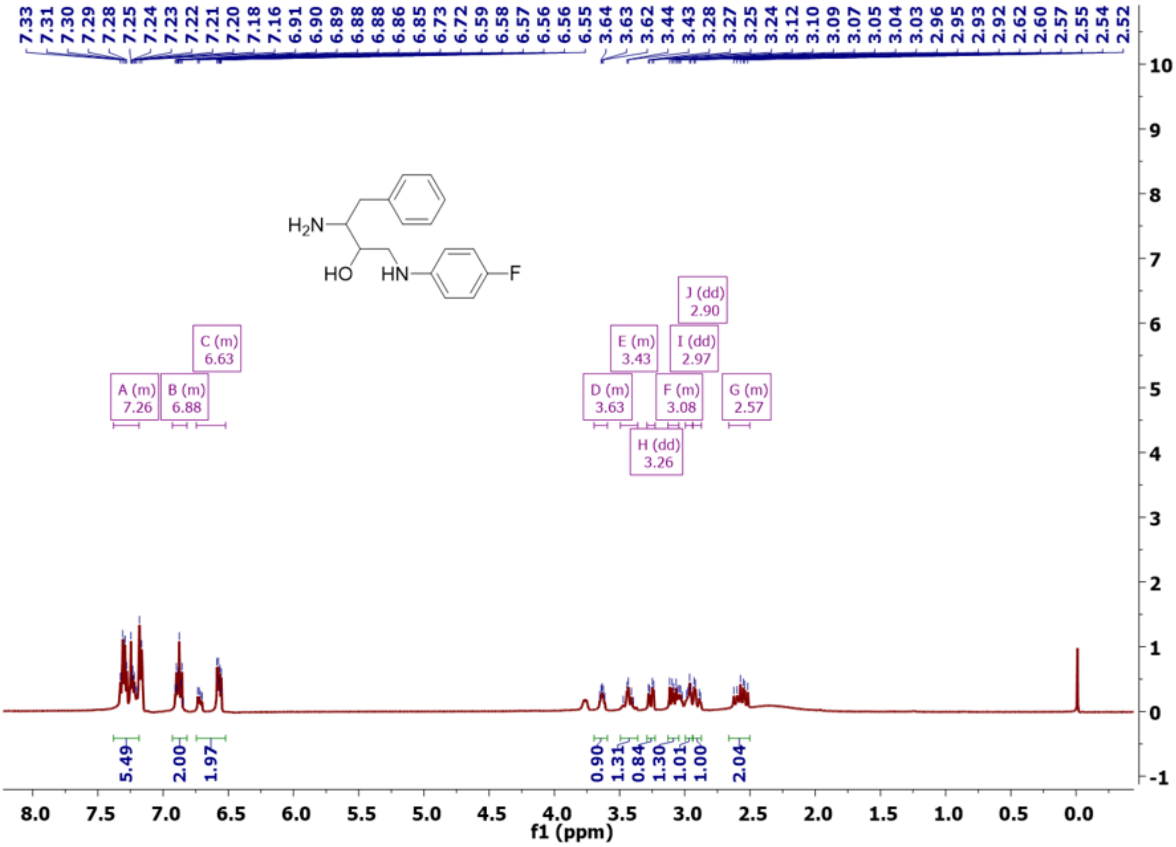

S1A Fig. ^{1}H NMR spectrum of **LTC-1026** in $CDCl_3$.

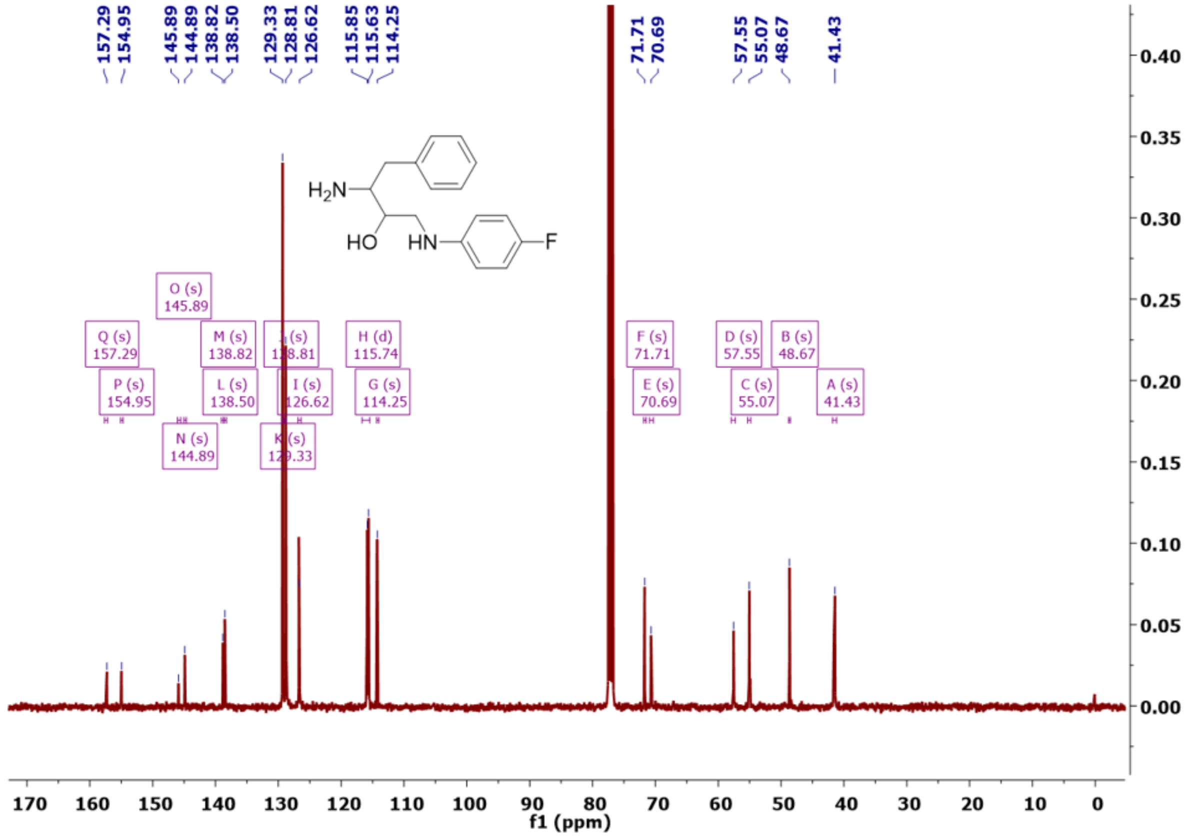

S1B Fig. ^{13}C NMR spectrum of **LTC-1026** in $CDCl_3$.

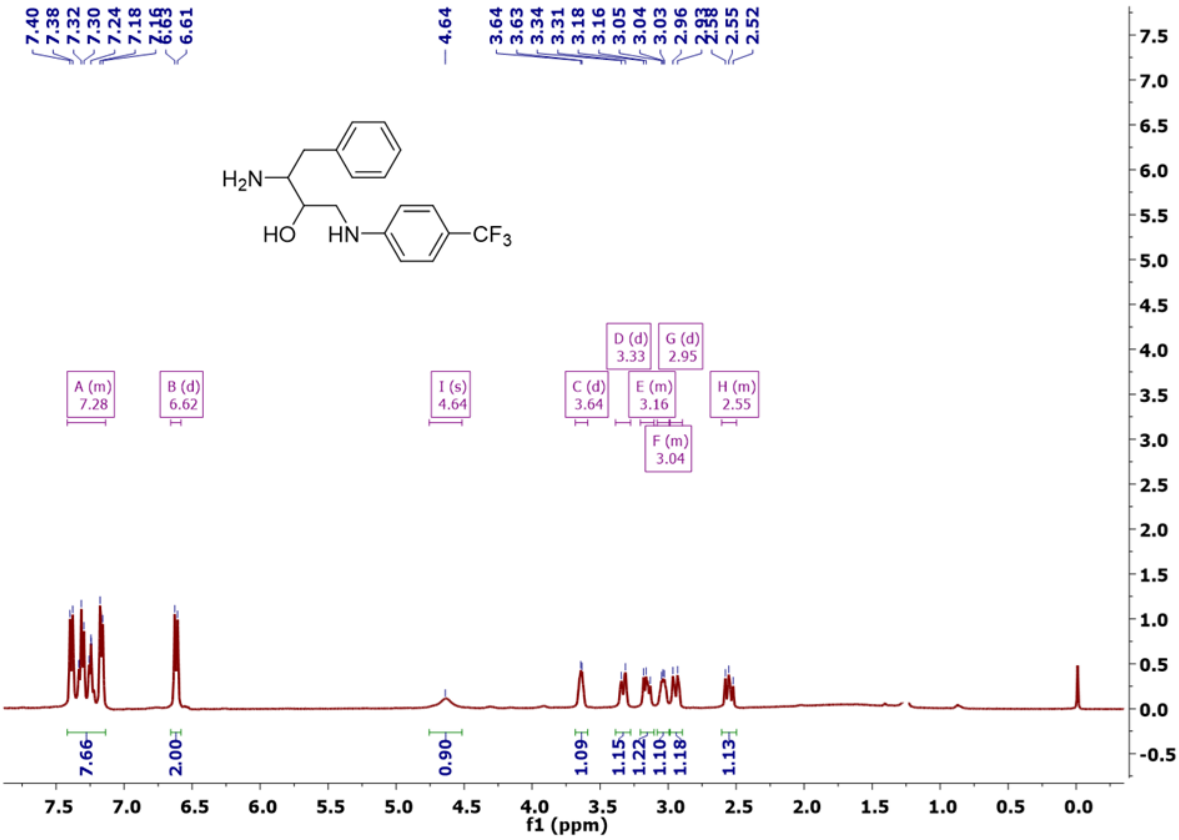

S1C Fig. ^{1}H NMR spectrum of **LTC-1027** in $CDCl_3$.

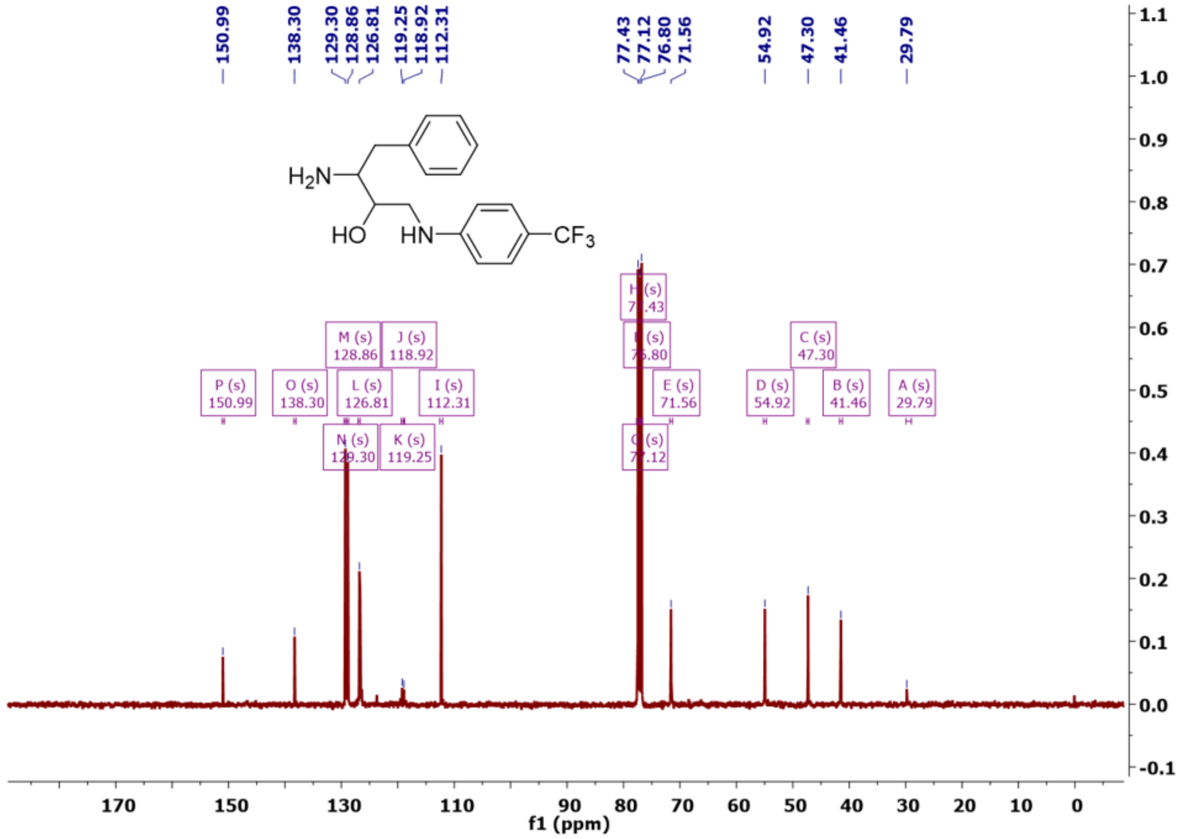

S1D Fig. ^{13}C NMR spectrum of **LTC-1027** in $CDCl_3$.

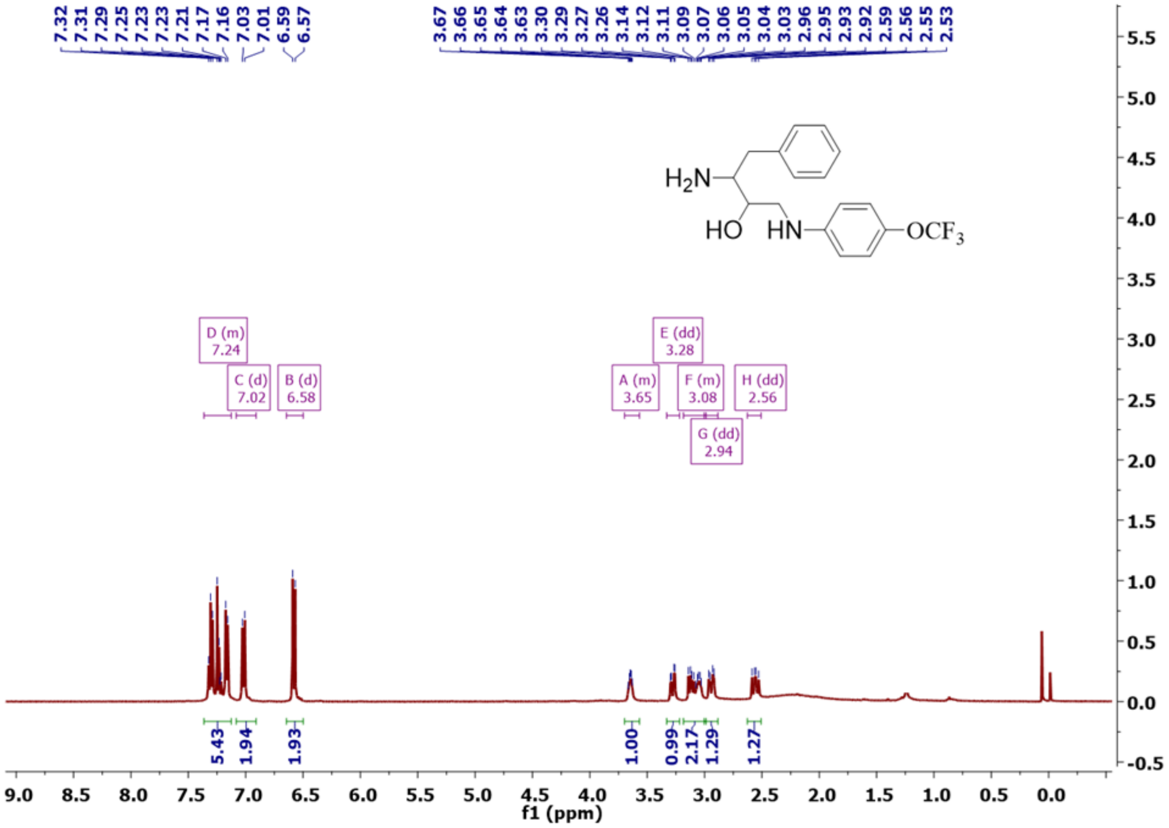

S 1 E Fig. ^{1}H NMR spectrum of **LTC-1028** in $CDCl_3$.

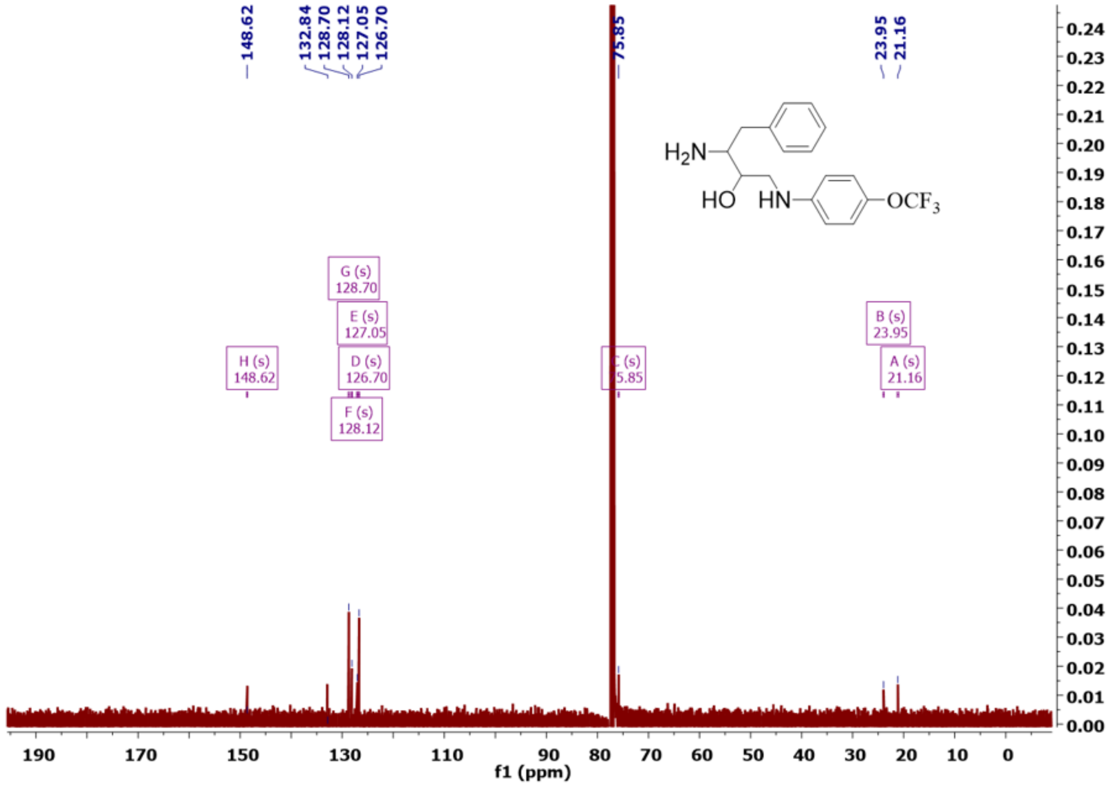

S1F Fig. ^{13}C NMR spectrum of **LTC-1028** in $CDCl_3$.

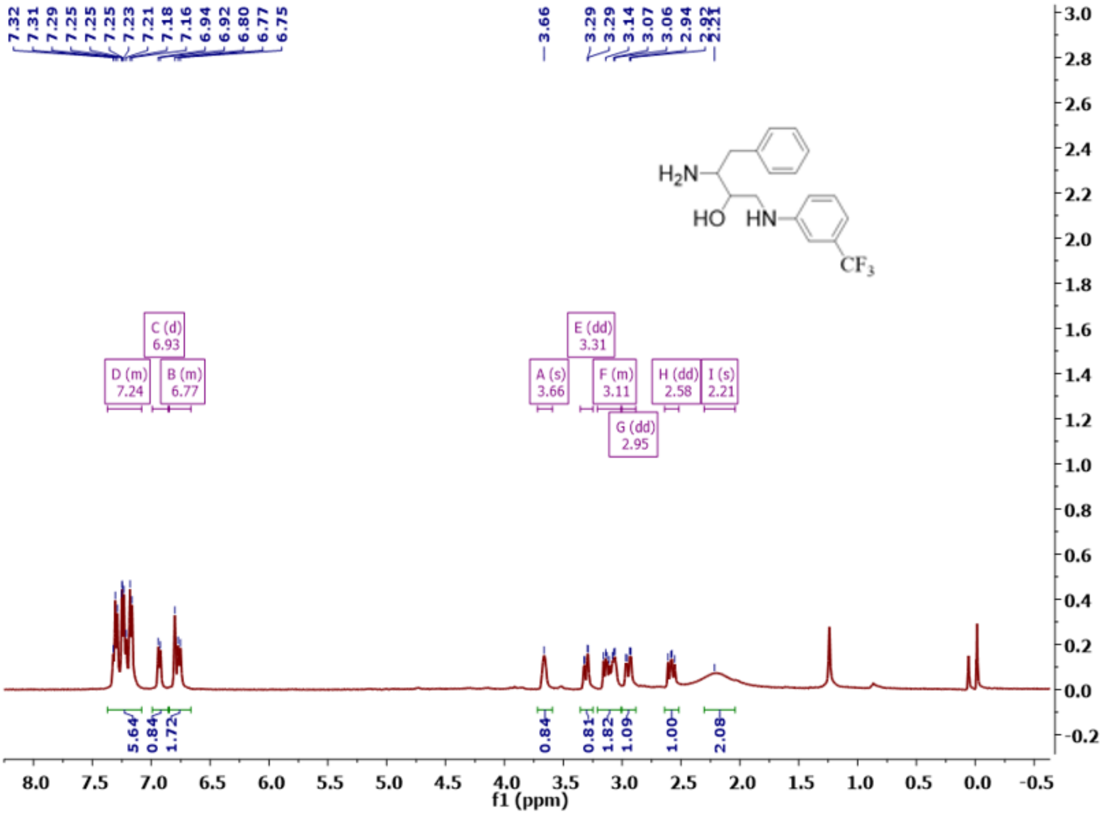

S1G Fig. ^{1}H NMR spectrum of **LTC-1029** in $CDCl_3$.

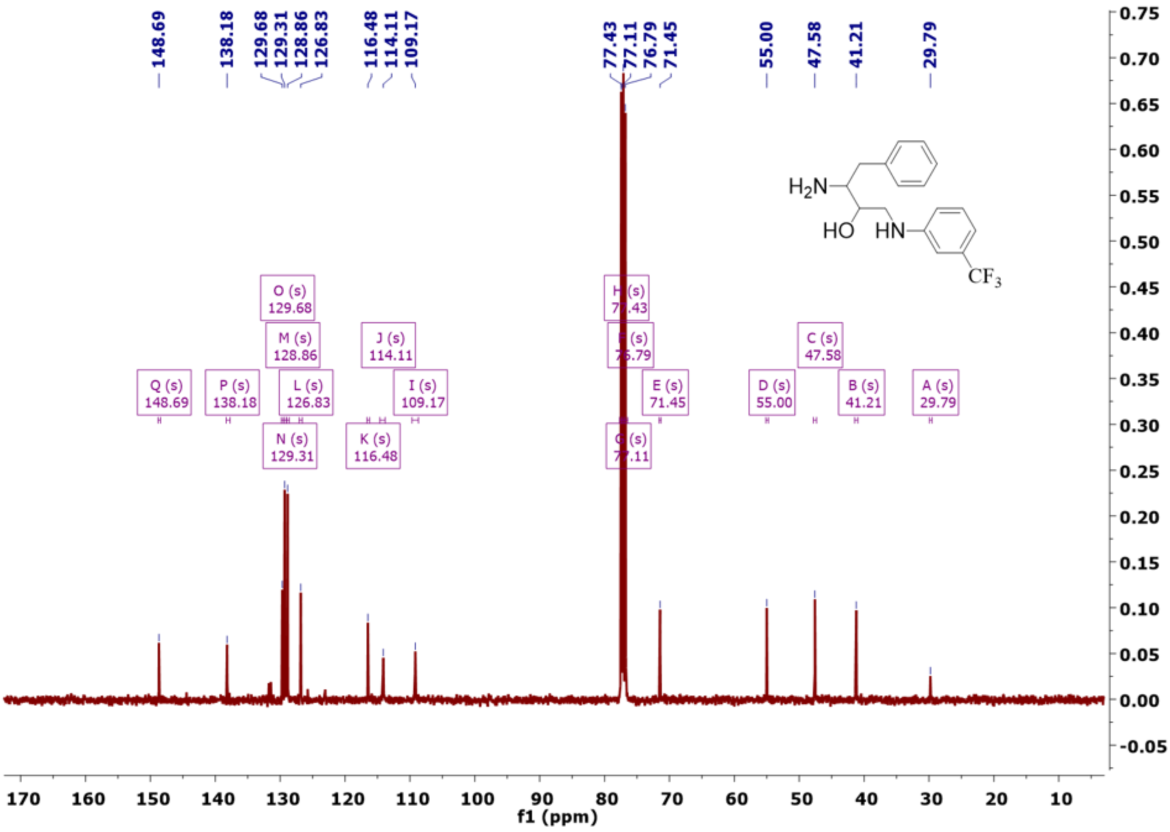

S1H Fig. ^{13}C NMR spectrum of **LTC-1029** in $CDCl_3$.

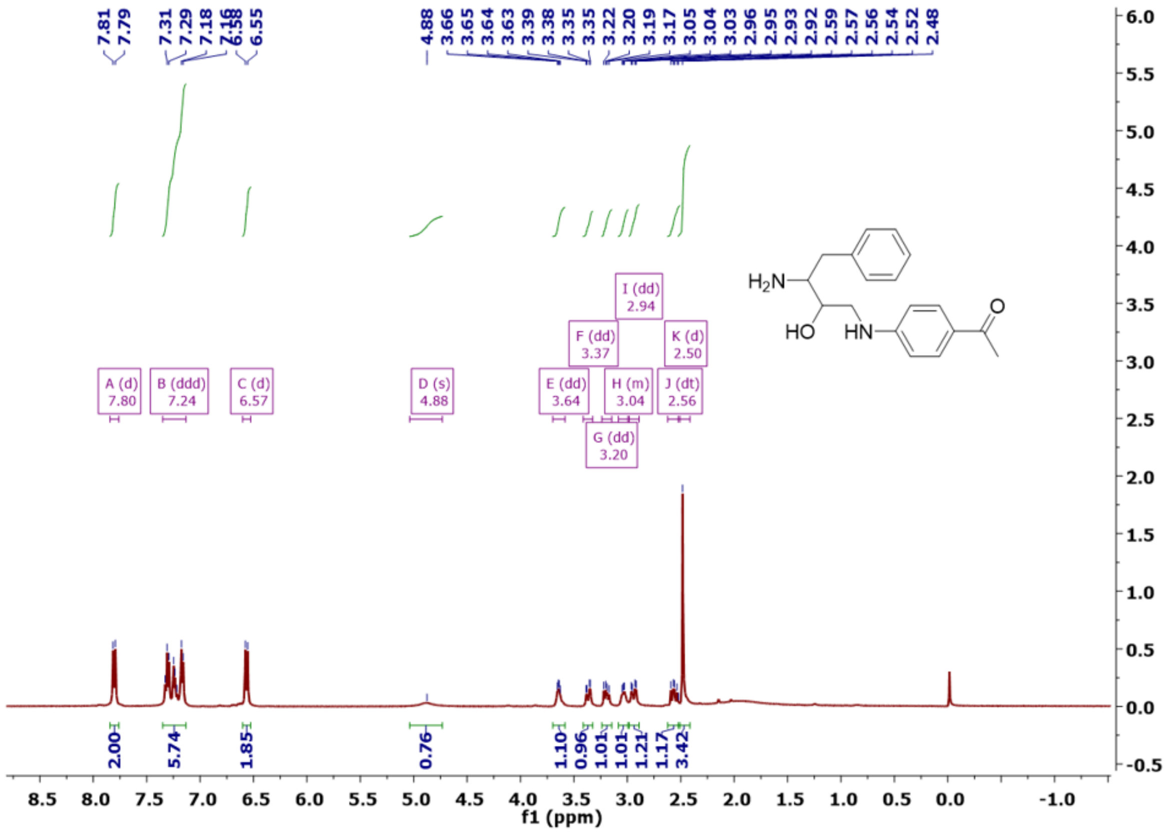

S1I Fig. ^{1}H NMR spectrum of **LTC-1031** in $CDCl_3$.

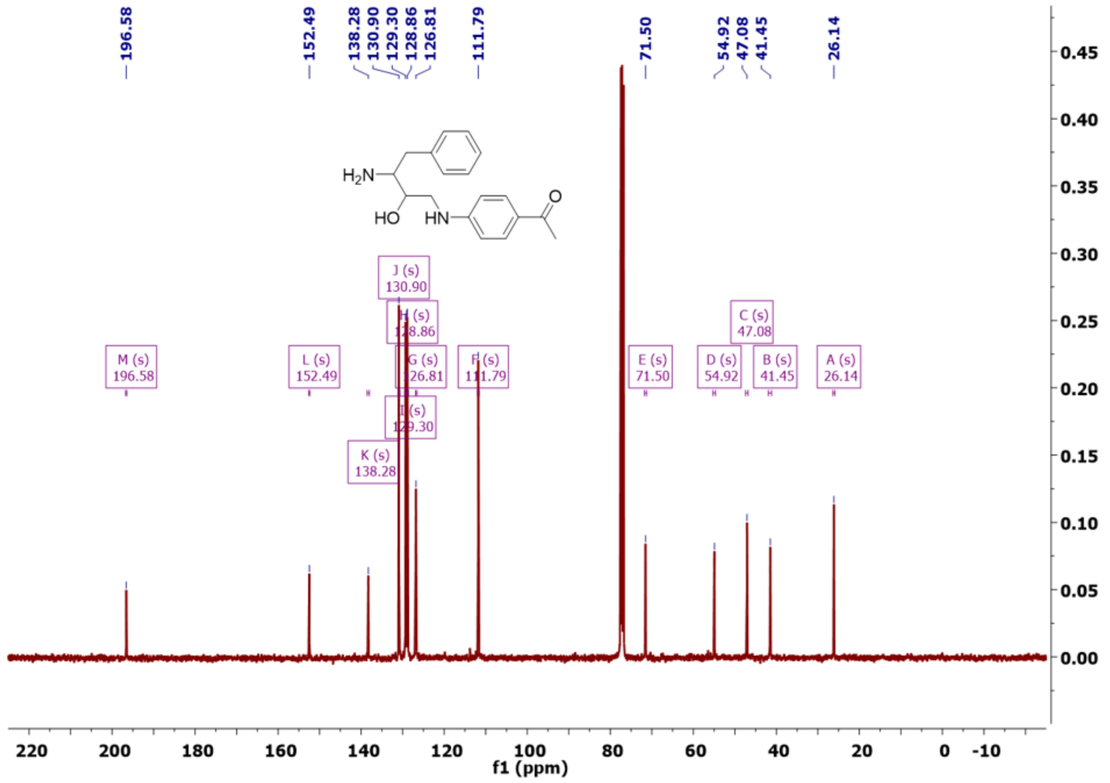

S1J Fig. ^{13}C NMR spectrum of **LTC-1031** in $CDCl_3$.

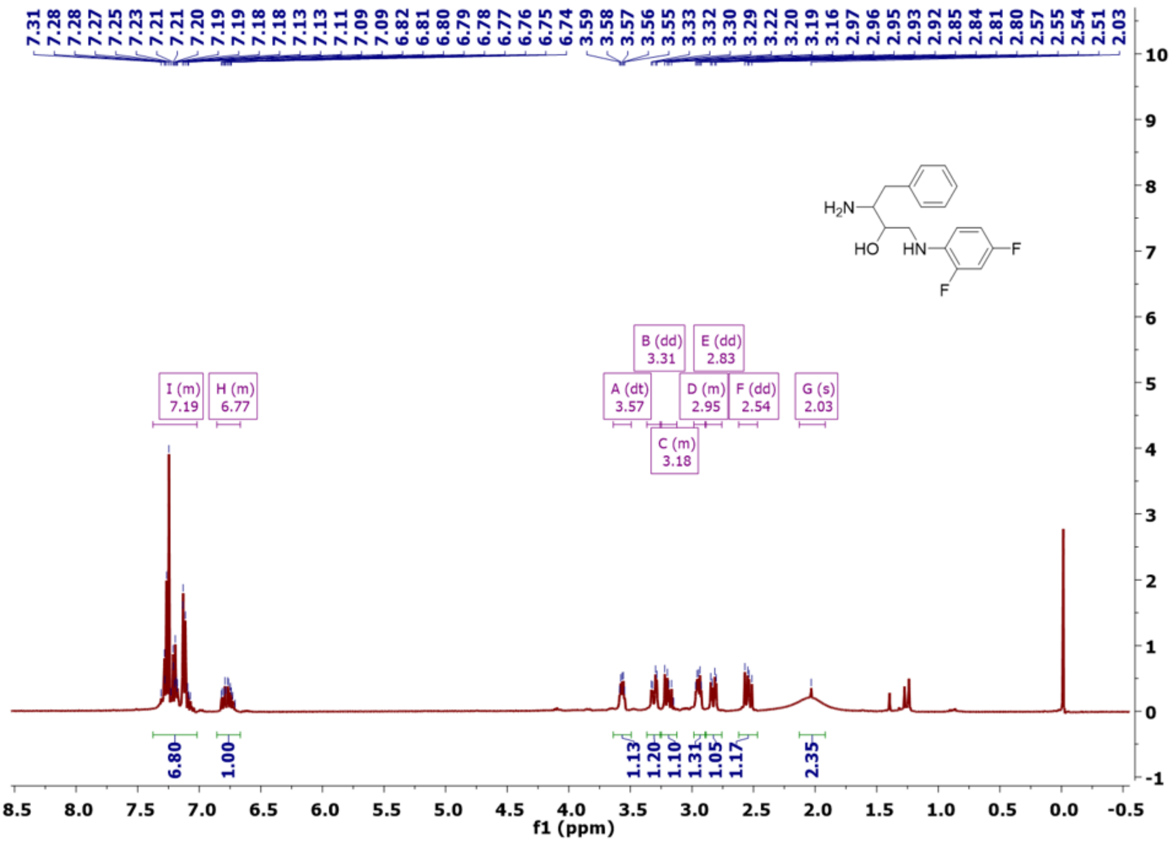

S1K Fig. ^{1}H NMR spectrum of **LTC-1032** in $CDCl_3$.

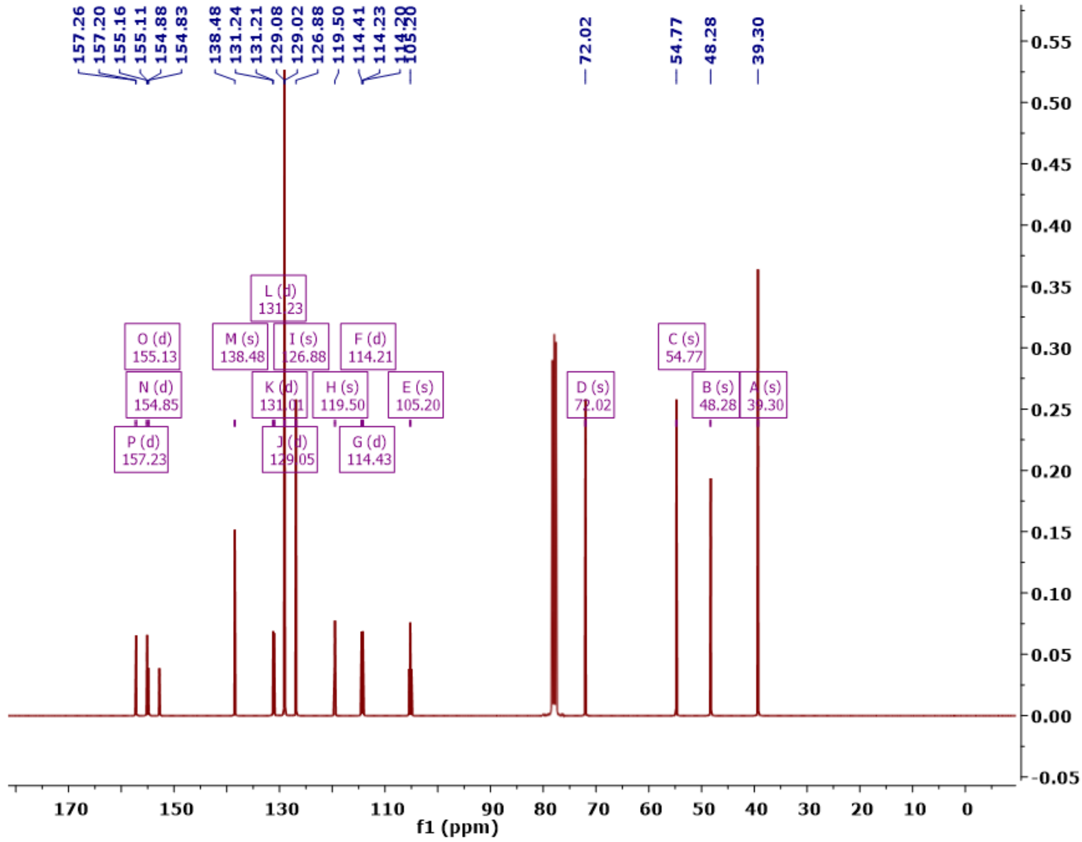

S1L Fig. ^{13}C NMR spectrum of **LTC-1032** in $CDCl_3$.

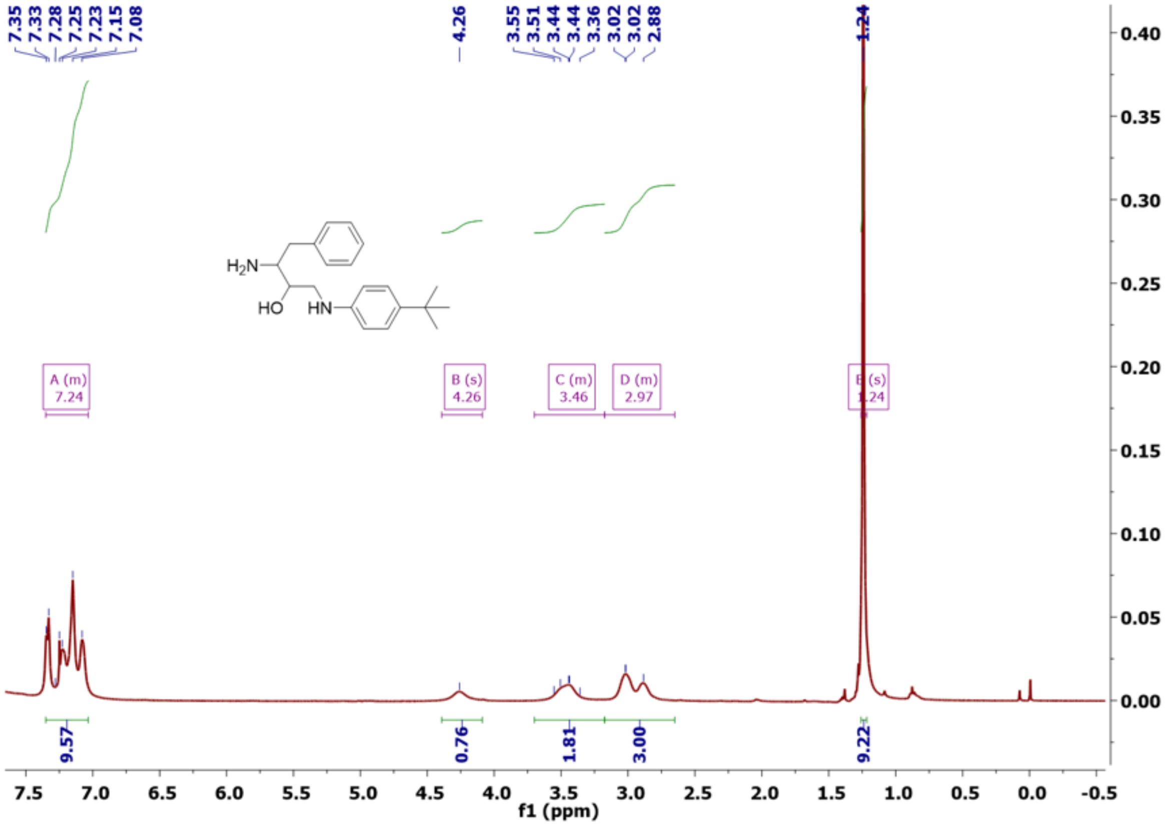

S1M Fig. ^{1}H NMR spectrum of **LTC-1034** in $CDCl_3$.

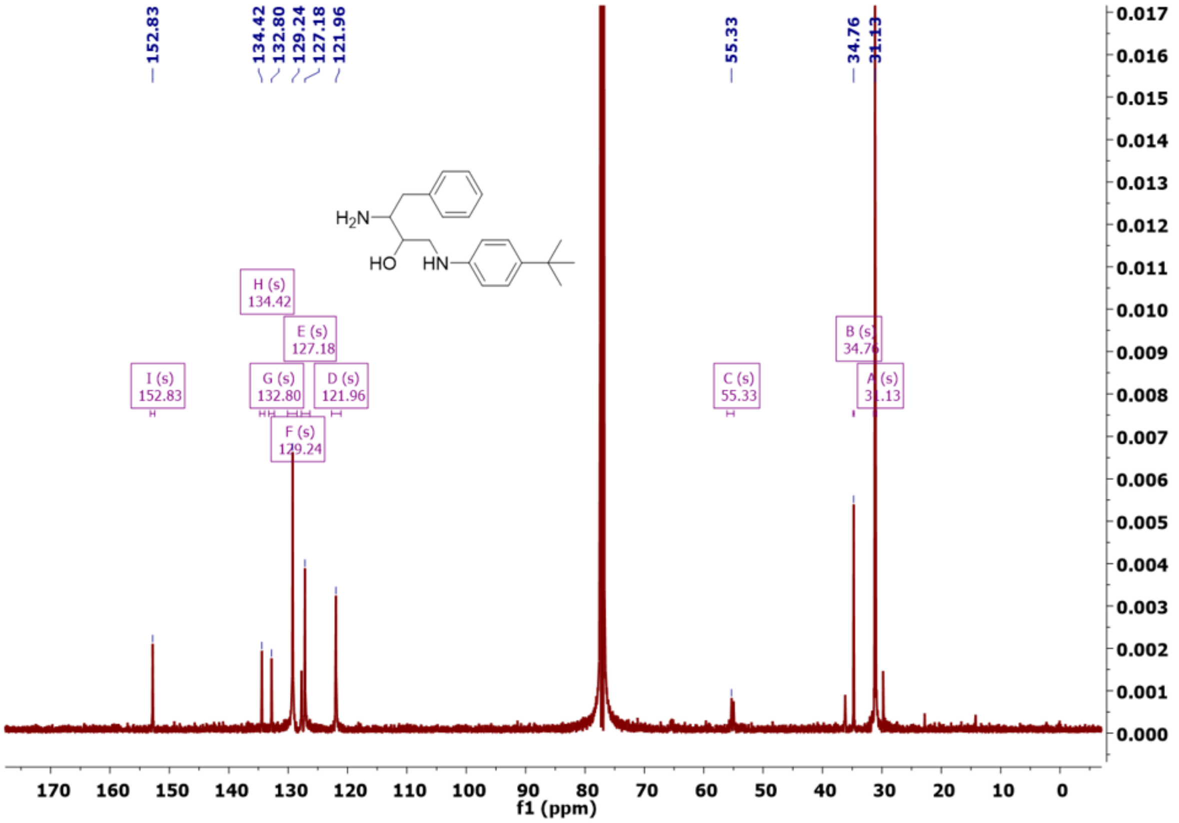

S1N Fig. ^{13}C NMR spectrum of **LTC-1034** in $CDCl_3$.

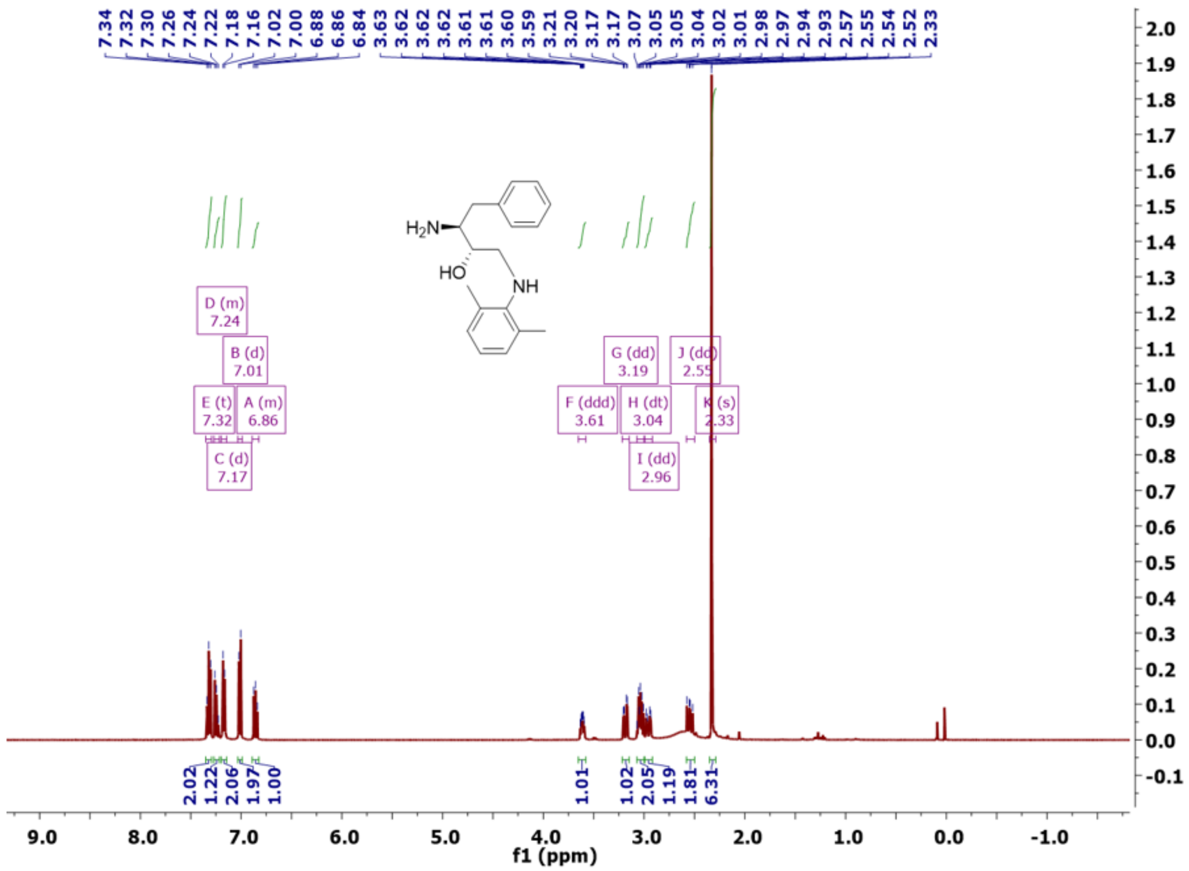

S1O Fig. ^{1}H NMR spectrum of **LTC-1041** in $CDCl_3$.

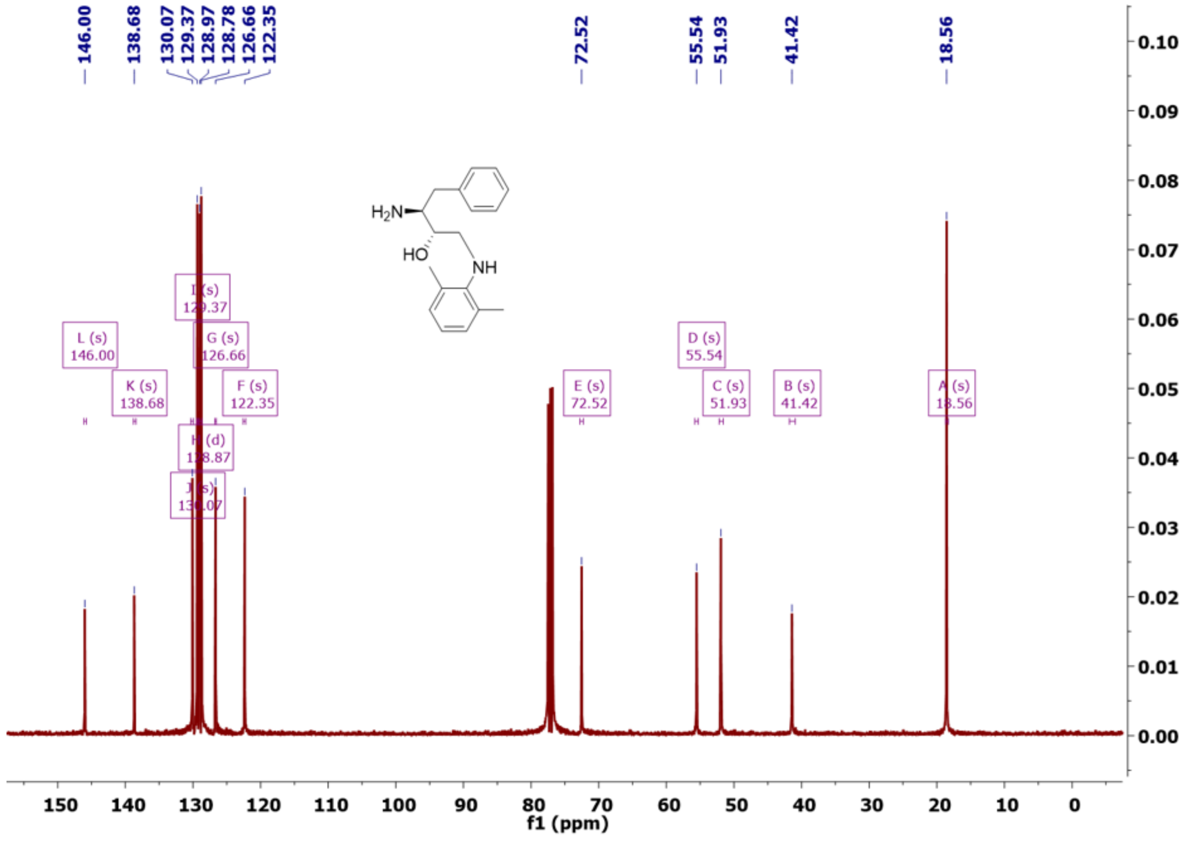

S1P Fig. ^{13}C NMR spectrum of **LTC-1041** in $CDCl_3$.

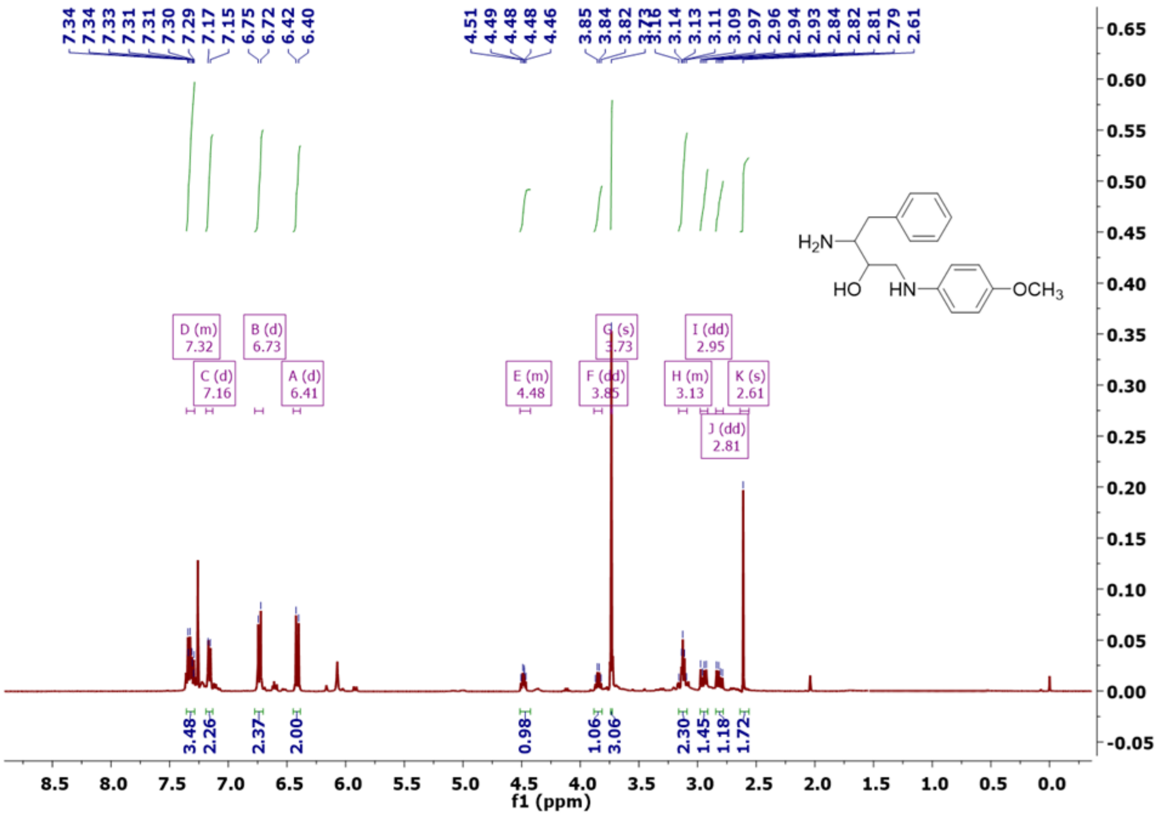

S1Q Fig. ^{1}H NMR spectrum of **LTC-1042** in $CDCl_3$.

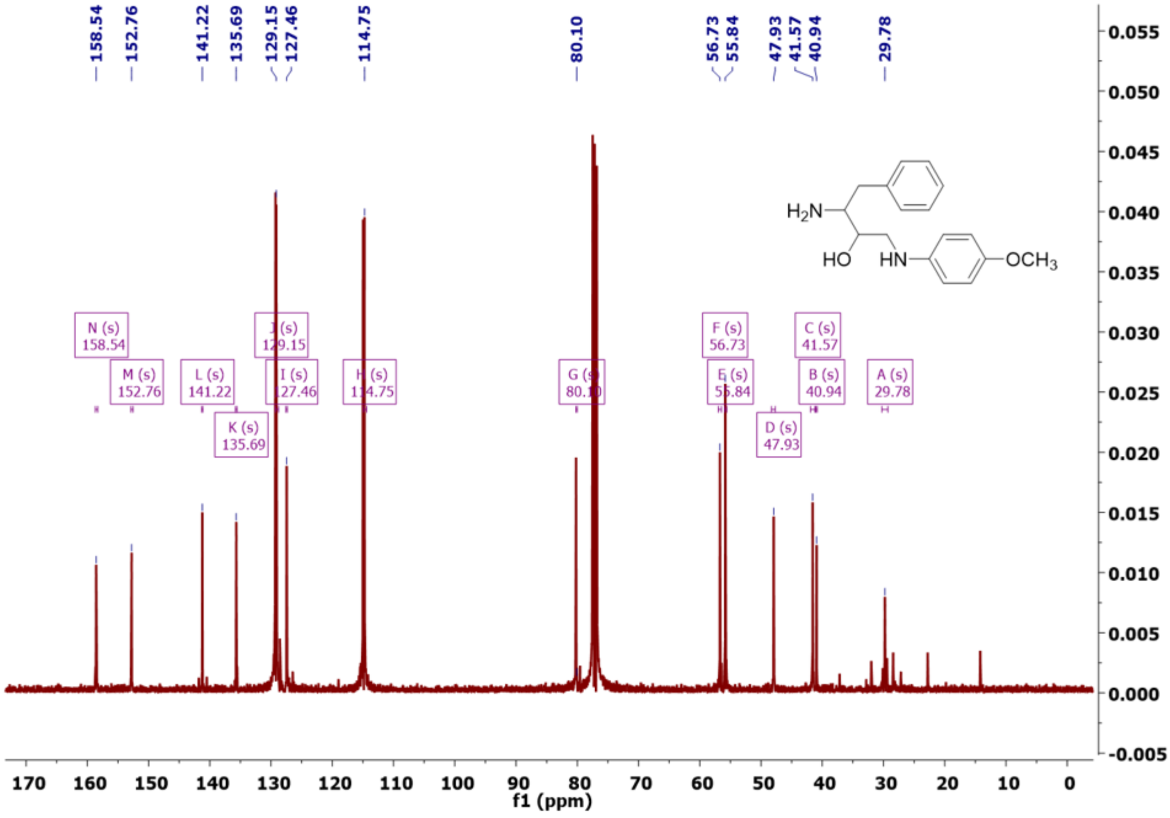

S1R Fig. ^{13}C NMR spectrum of **LTC-1042** in $CDCl_3$.

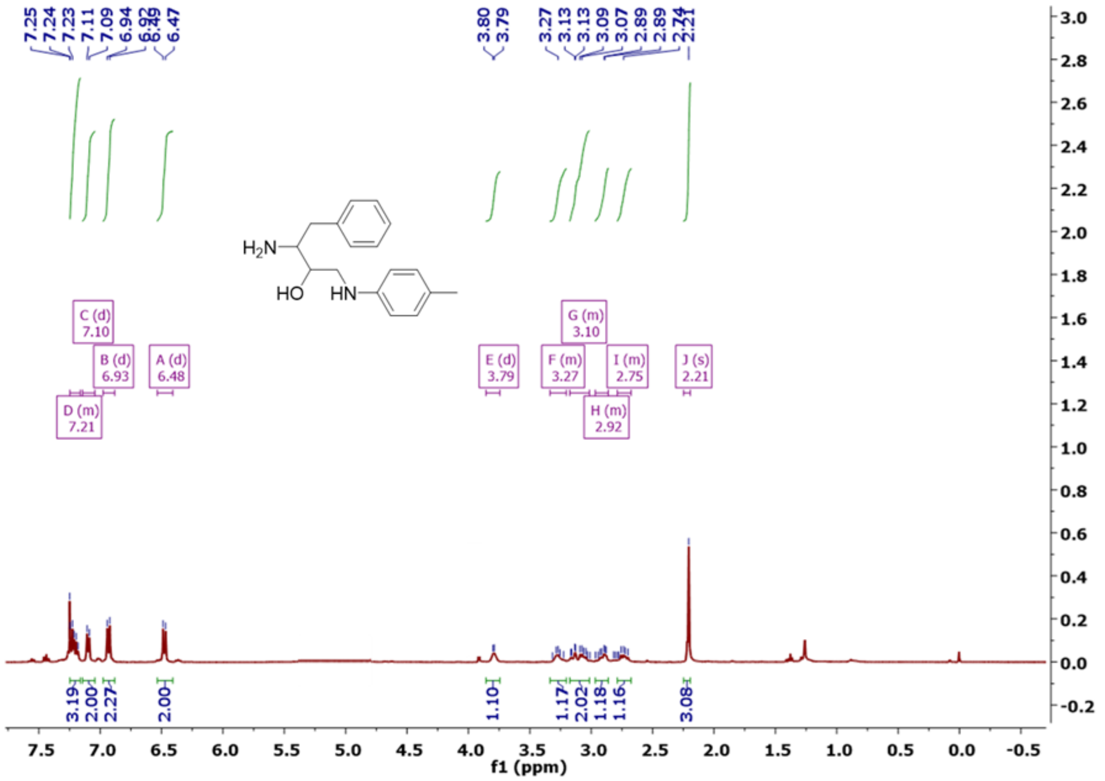

S1S Fig. ^{1}H NMR spectrum of **LTC-1043** in $CDCl_3$.

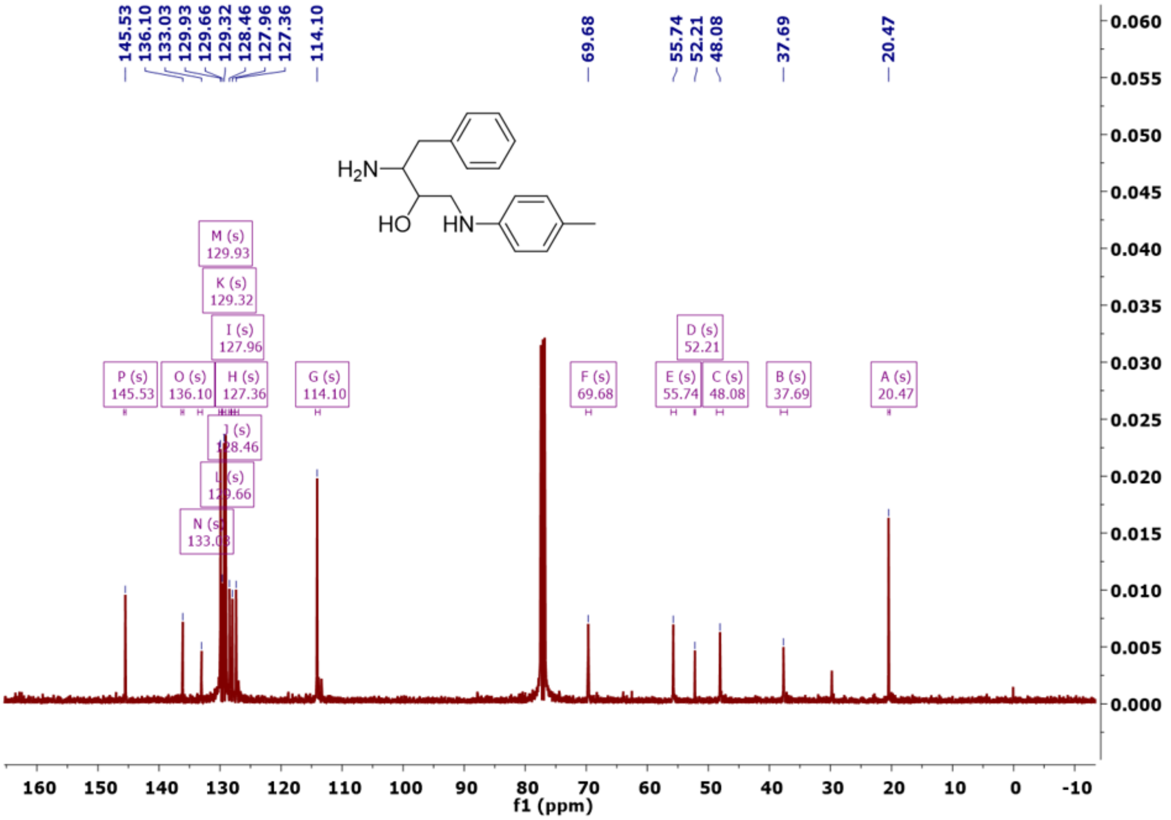

S1T Fig. ^{13}C NMR spectrum of **LTC-1043** in $CDCl_3$.
