# Supplementary material for: Phenotypic screening reveals a highly selective phthalimide-based compound with antileishmanial activity
Source: PLoS Negl Trop Dis. 2024 Mar 25;18(3):e0012050. doi: 10.1371/journal.pntd.0012050 (PMC10994559; doi:10.1371/journal.pntd.0012050)
Supplement: S2 Fig — (PDF) [file pntd.0012050.s002.pdf]

S2 Fig. HPLC data

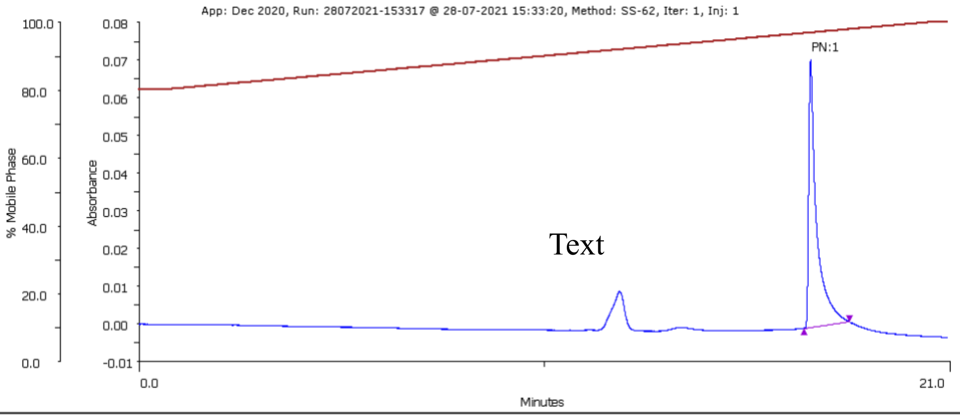

Sample Table

Injection Number	Peak Name	Retention Time (min)	Area (mAUmin x100)	Height (Absorbance)	Sample Name	Sample Location	Peak Purity	Amount (mg)	
1	1	17.418	1700.0289	0.071	ph 39	Sample Zone 1->1	987.564	0	

S2A Figure. HPLC purity analysis for PHT-39 in 95:5 ratios of acetonitrile and water (98.7% pure).

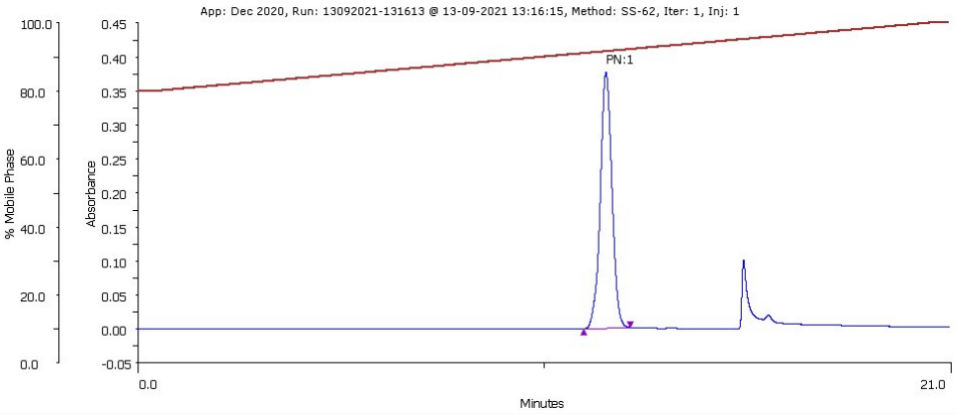

Sample Table

Injection Number	Peak Name	Retention Time (min)	Area (mAUmin x100)	Height (Absorbance)	Sample Name	Sample Location	Peak Purity	Amount (mg)	
1	1	12.11	13140.7306	0.376	PH-40	Sample Zone 1->1	986.983	0	

S2B Figure. HPLC purity analysis for PHT-40 in 95:5 ratios of acetonitrile and water (98.6% pure).

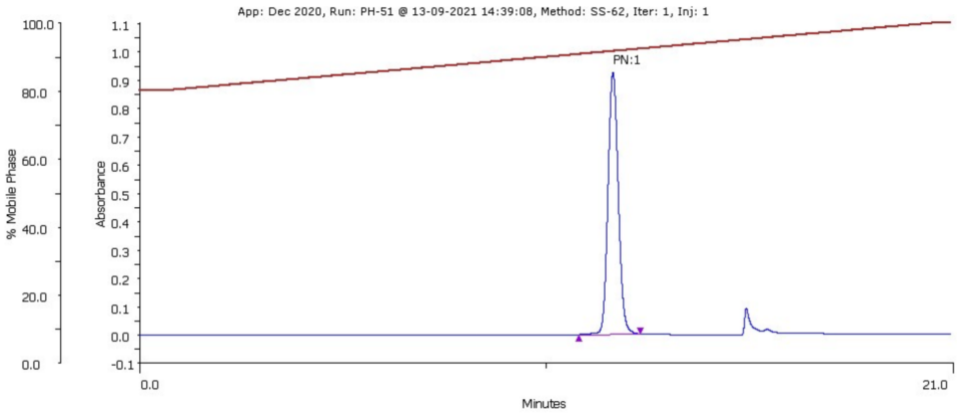

Sample Table

Injection Number	Peak Name	Retention Time (min)	Area (mAUmin x100)	Height (Absorbance)	Sample Name	Sample Location	Peak Purity	Amount (mg)
1	1	12.238	28565.3735	0.924	PH-51	Sample Zone 1->1	975.577	0

S2C Figure. HPLC purity analysis for PHT-51 in 95:5 ratios of acetonitrile and water (97.5% pure).

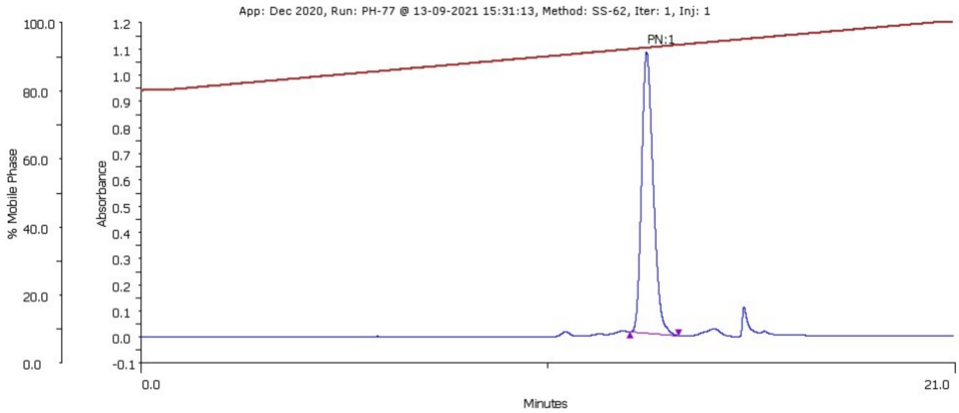

Sample Table

Injection Number	Peak Name	Retention Time (min)	Area (mAUmin x100)	Height (Absorbance)	Sample Name	Sample Location	Peak Purity	Amount (mg)
1	1	13.07	37565.2812	1.075	PH-77	Sample Zone 1->1	991.369	0

S2D Figure. HPLC purity analysis for PHT-77 in 95:5 ratios of acetonitrile and water (99.1% pure).

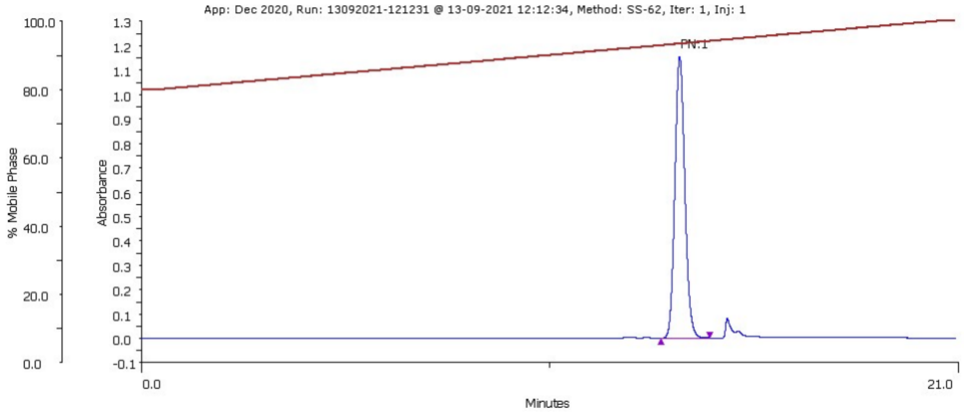

Sample Table

Injection Number	Peak Name	Retention Time (min)	Area (mAUmin x100)	Height (Absorbance)	Sample Name	Sample Location	Peak Purity	Amount (mg)
1	1	13.863	34388.0024	1.155	PH-79	Sample Zone 1->1	995.321	0

S2E Figure. HPLC purity analysis for PHT-79 in 95:5 ratios of acetonitrile and water (99.5% pure).

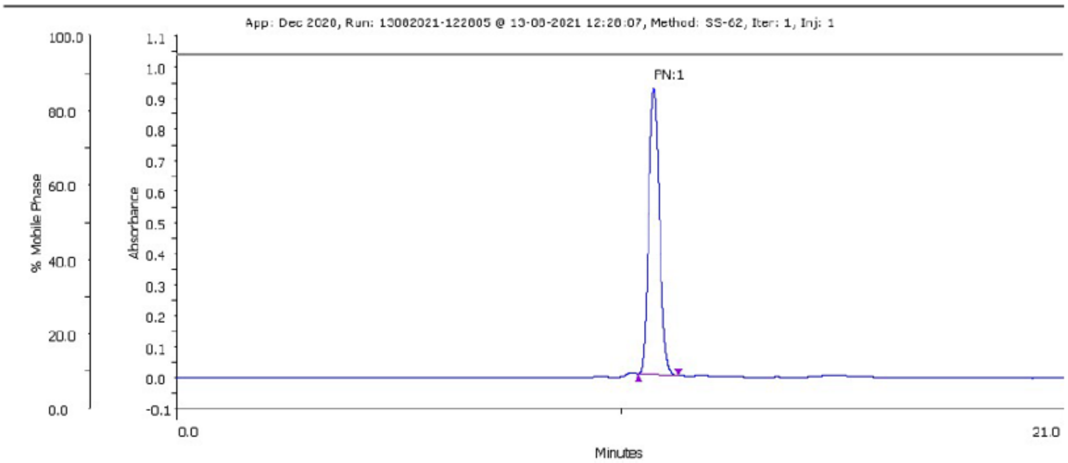

Sample Table

Injection Number	Peak Name	Retention Time (min)	Area (mAUmin x100)	Height (Absorbance)	Sample Name	Sample Location	Peak Purity
1	1	11.287	27047.9643	0.921	LTC-1025	Sample Zone 1->1	979.353

S2F Figure. HPLC purity analysis for LTC-1025 in 95:5 ratios of acetonitrile and water (97.9% pure).

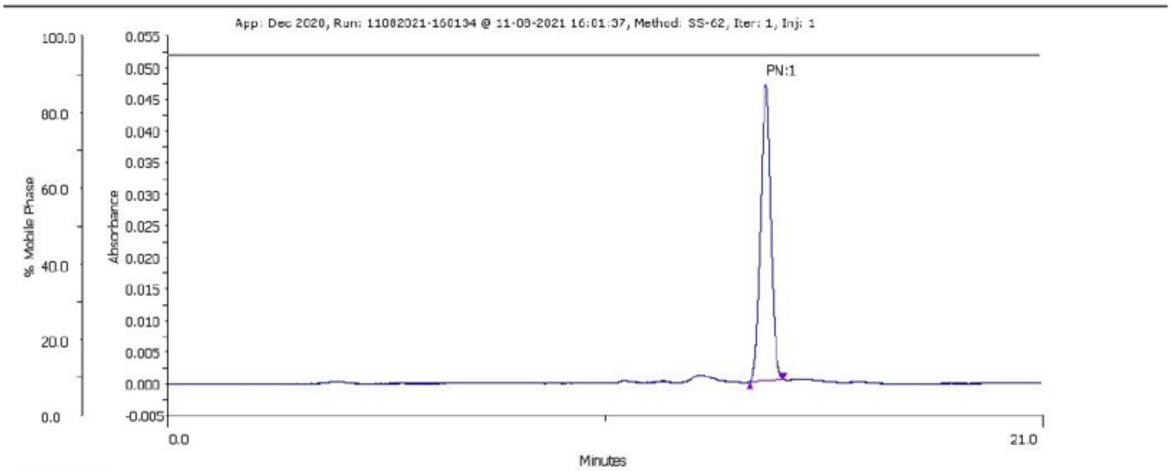

Sample Table

Injection Number	Peak Name	Retention Time (min)	Area (mAUmin x100)	Height (Absorbance)	Sample Name	Sample Location	Peak Purity	Amount (mg)
1	1	14.375	1338.2897	0.047	LTC-1035	Sample Zone 1->1	998.418	0

S2G Figure. HPLC purity analysis for LTC-1035 in 95:5 ratios of acetonitrile and water (99.8% pure).
