# Supplementary material for: Phenotypic screening reveals a highly selective phthalimide-based compound with antileishmanial activity
Source: PLoS Negl Trop Dis. 2024 Mar 25;18(3):e0012050. doi: 10.1371/journal.pntd.0012050 (PMC10994559; doi:10.1371/journal.pntd.0012050)
Supplement: S3 Fig — Primary HEA and PHT compound library screens for all target organisms, A) L. major promastigote response to PHT compounds (25 μM), B) L. major promastigote response to HEA compounds, C) L. infantum promastigote response to PHT compounds. D) L. infantum promastigote response to HEA compounds, E) L. major amastigote response to PHT compounds, F) L. major amastigote response to HEA compounds (25 μM), G) L. major amastigote response to HEA compounds (2.5 μM), H) L. infantum rescue assay for PHT compounds (25 μM), I) L. infantum rescue assay for HEA compounds (25 μM), J) Toxicity assay in J774 cells for PHT compounds, K) Toxicity assay in J774 for HEA compounds L) T. brucei BSF response in PHT compounds, M) T. brucei BSF response to HEA compounds, N) T. cruzi response to PHT compounds, O) T. cruzi response to HEA compounds, P) E. histolytica response to PHT compounds. Q) E. histolytica response to HEA compounds. (PDF) [file pntd.0012050.s003.pdf]

S3 Fig.

(A)

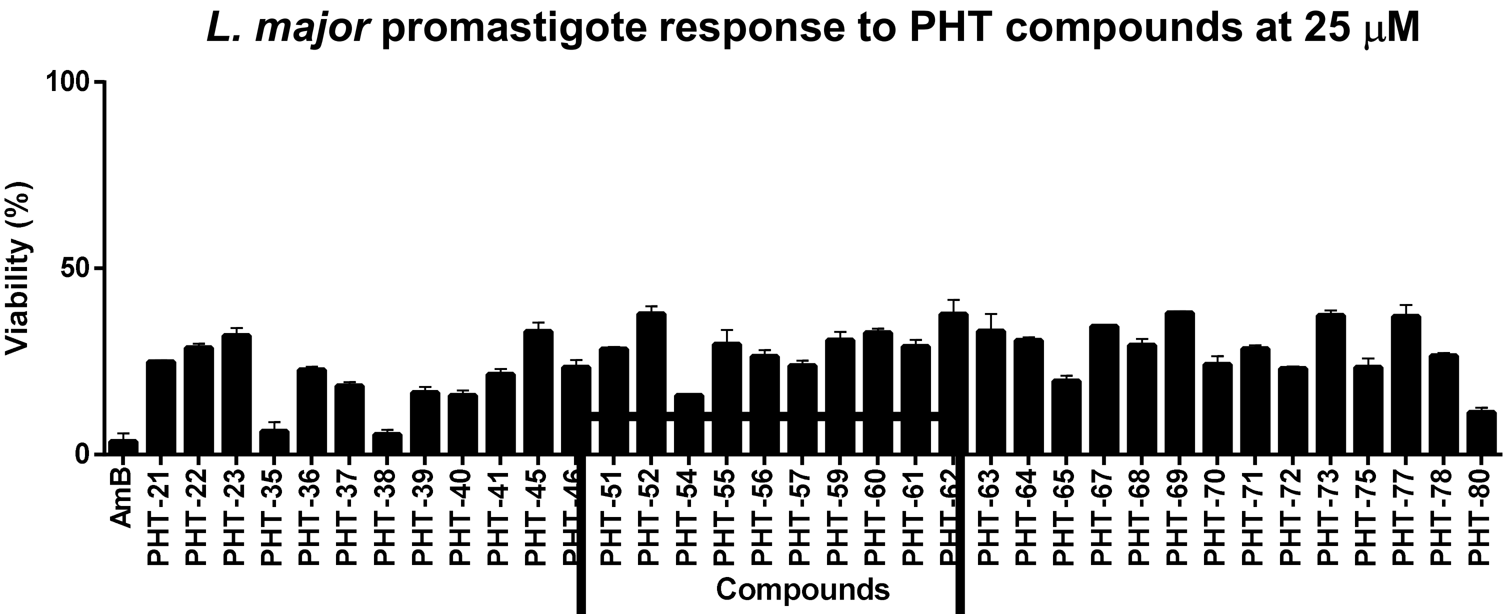

S3 Fig. Primary HEA and PHT compound library screens for all target organisms. A) *L. major* promastigote response to PHT compounds (25 µM), B) *L. major* promastigote response to HEA compounds, C) *L. infantum* promastigote response to PHT compounds. D) *L. infantum* promastigote response to HEA compounds, E) *L. major* amastigote response to PHT compounds, F) *L. major* amastigote response to HEA compounds (25 µM), G) *L. major* amastigote response to HEA compounds (2.5 µM), H) *L. infantum* rescue assay for PHT compounds (25 µM), I) *L. infantum* rescue assay for HEA compounds (25 µM), J) Toxicity assay in J774 cells for PHT compounds, K) Toxicity assay in J774 for HEA compounds L) *T. brucei* BSF response in PHT compounds, M) *T. brucei* BSF response to HEA compounds, N) *T. cruzi* response to PHT compounds, O) *T. cruzi* response to HEA compounds, P) *E. histolytica* response to PHT compounds. Q) *E. histolytica* response to HEA compounds.

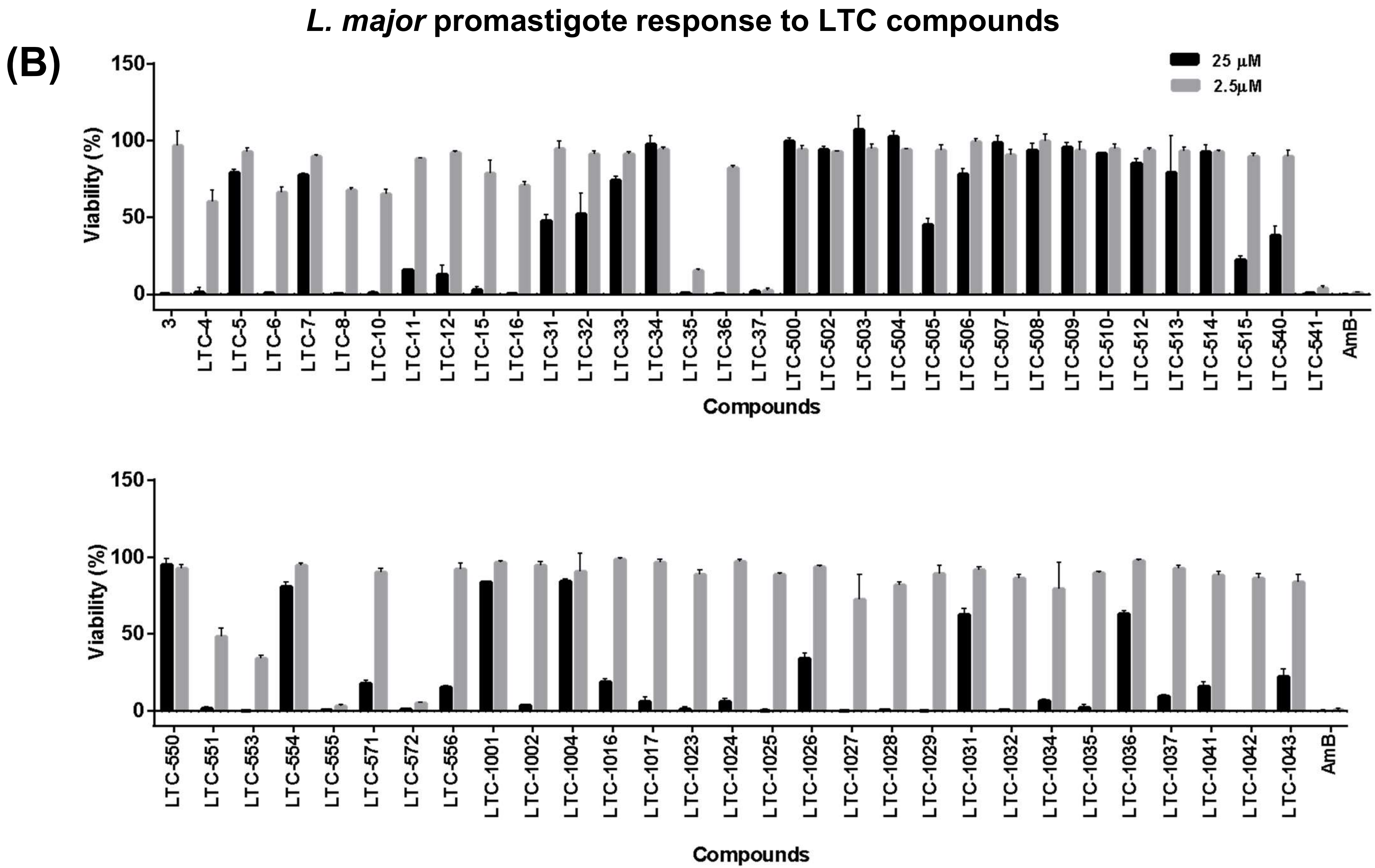
L. major promastigote response to LTC compounds
(B)
25 µM
2.5µM
Viability (%)
150
100
50
0
3
LTC-4
LTC-5
LTC-6
LTC-7
LTC-8
LTC-10
LTC-11
LTC-12
LTC-15
LTC-16
LTC-31
LTC-32
LTC-33
LTC-34
LTC-35
LTC-36
LTC-37
LTC-500
LTC-502
LTC-503
LTC-504
LTC-505
LTC-506
LTC-507
LTC-508
LTC-509
LTC-510
LTC-512
LTC-513
LTC-514
LTC-515
LTC-540
LTC-541
AmB
Compounds
Viability (%)
150
100
50
0
LTC-550
LTC-551
LTC-553
LTC-554
LTC-555
LTC-571
LTC-572
LTC-556
LTC-1001
LTC-1002
LTC-1004
LTC-1016
LTC-1017
LTC-1023
LTC-1024
LTC-1025
LTC-1026
LTC-1027
LTC-1028
LTC-1029
LTC-1031
LTC-1032
LTC-1034
LTC-1035
LTC-1036
LTC-1037
LTC-1041
LTC-1042
LTC-1043
AmB
Compounds

(C)

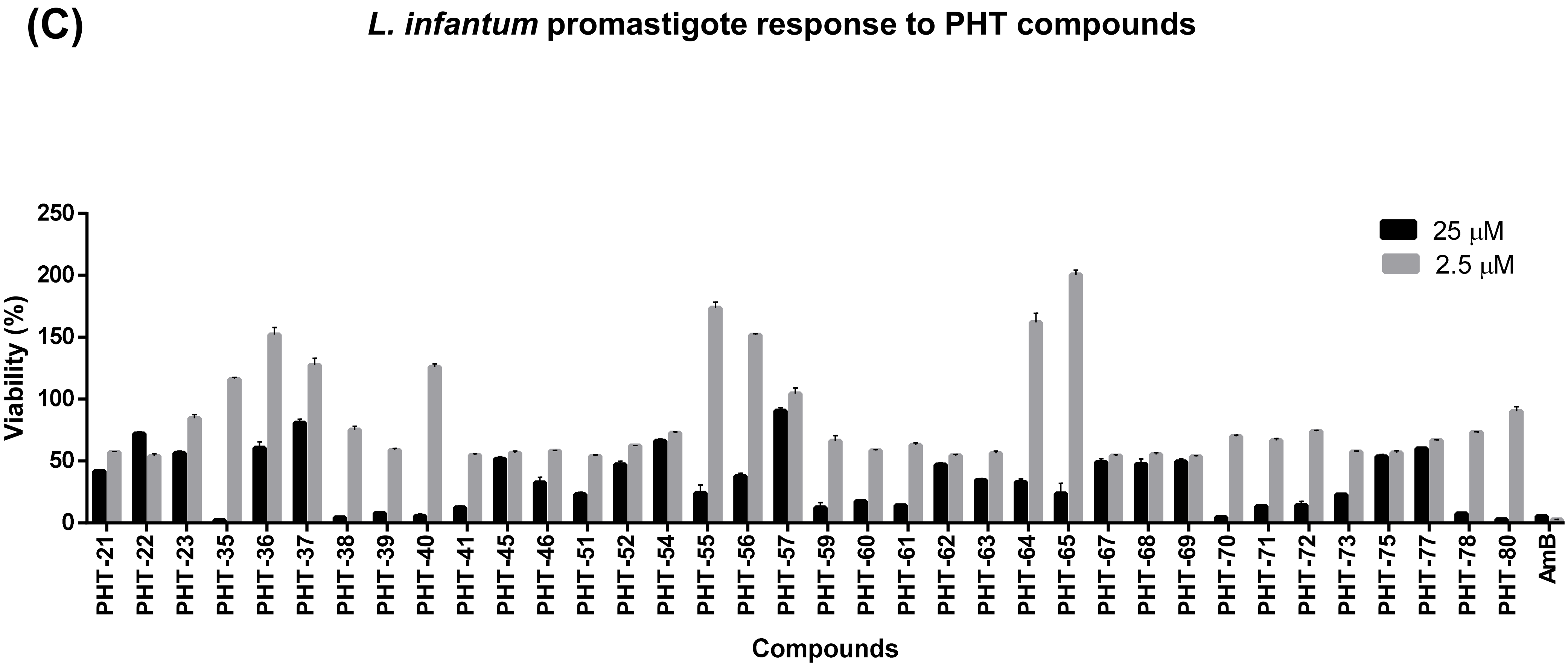

L. infantum promastigote response to PHT compounds
25 μM
2.5 μM
Viability (%)
250
200
150
100
50
0
PHT-21
PHT-22
PHT-23
PHT-35
PHT-36
PHT-37
PHT-38
PHT-39
PHT-40
PHT-41
PHT-45
PHT-46
PHT-51
PHT-52
PHT-54
PHT-55
PHT-56
PHT-57
PHT-59
PHT-60
PHT-61
PHT-62
PHT-63
PHT-64
PHT-65
PHT-67
PHT-68
PHT-69
PHT-70
PHT-71
PHT-72
PHT-73
PHT-75
PHT-77
PHT-78
PHT-80
AmB
Compounds

(D)

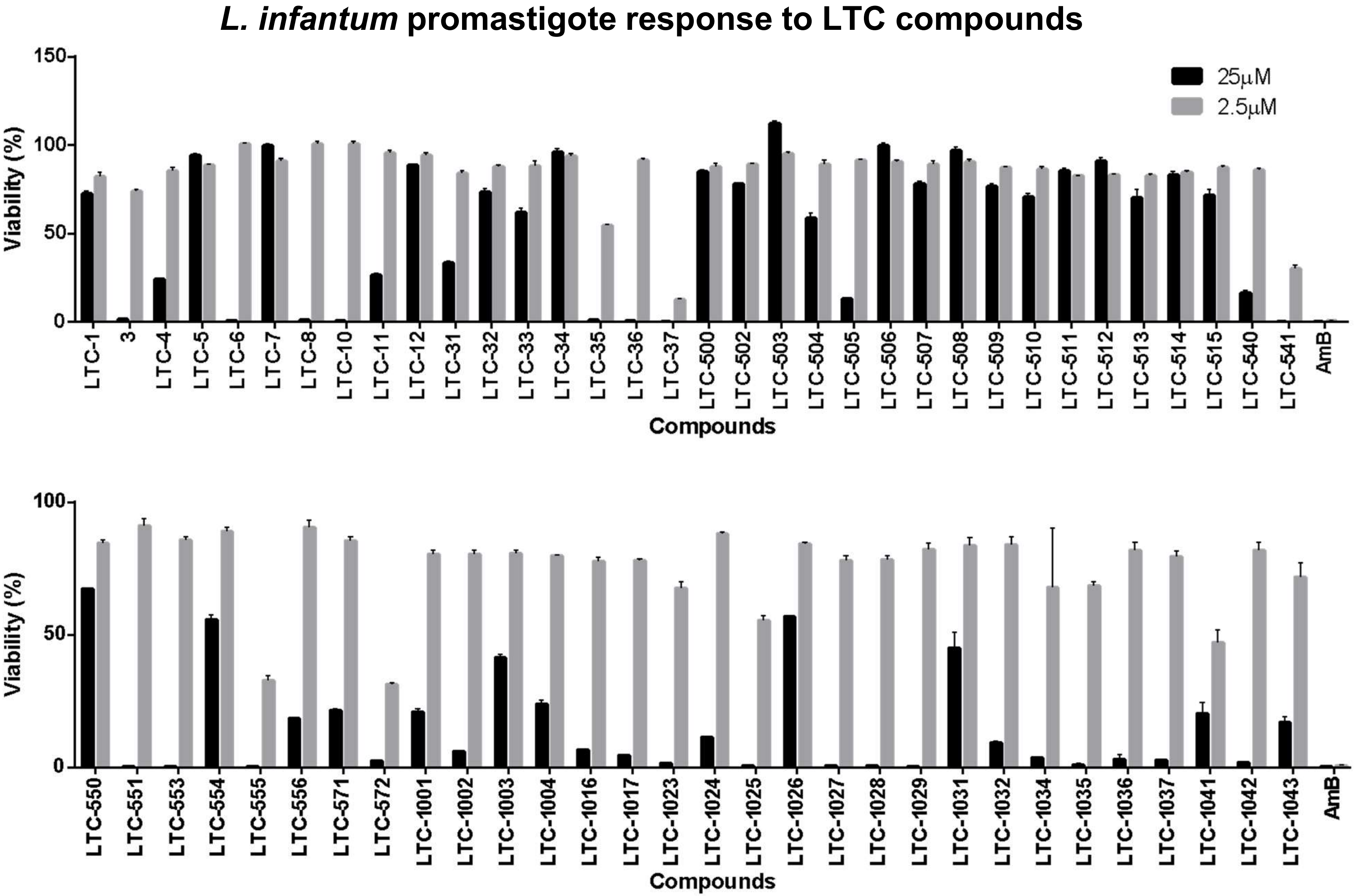
L. infantum promastigote response to LTC compounds
Viability (%)
25µM
2.5µM
Compounds
LTC-1
3
LTC-4
LTC-5
LTC-6
LTC-7
LTC-8
LTC-10
LTC-11
LTC-12
LTC-31
LTC-32
LTC-33
LTC-34
LTC-35
LTC-36
LTC-37
LTC-500
LTC-502
LTC-503
LTC-504
LTC-505
LTC-506
LTC-507
LTC-508
LTC-509
LTC-510
LTC-511
LTC-512
LTC-513
LTC-514
LTC-515
LTC-540
LTC-541
AmB
LTC-550
LTC-551
LTC-553
LTC-554
LTC-555
LTC-556
LTC-571
LTC-572
LTC-1001
LTC-1002
LTC-1003
LTC-1004
LTC-1016
LTC-1017
LTC-1023
LTC-1024
LTC-1025
LTC-1026
LTC-1027
LTC-1028
LTC-1029
LTC-1031
LTC-1032
LTC-1034
LTC-1035
LTC-1036
LTC-1037
LTC-1041
LTC-1042
LTC-1043
AmB

(E)

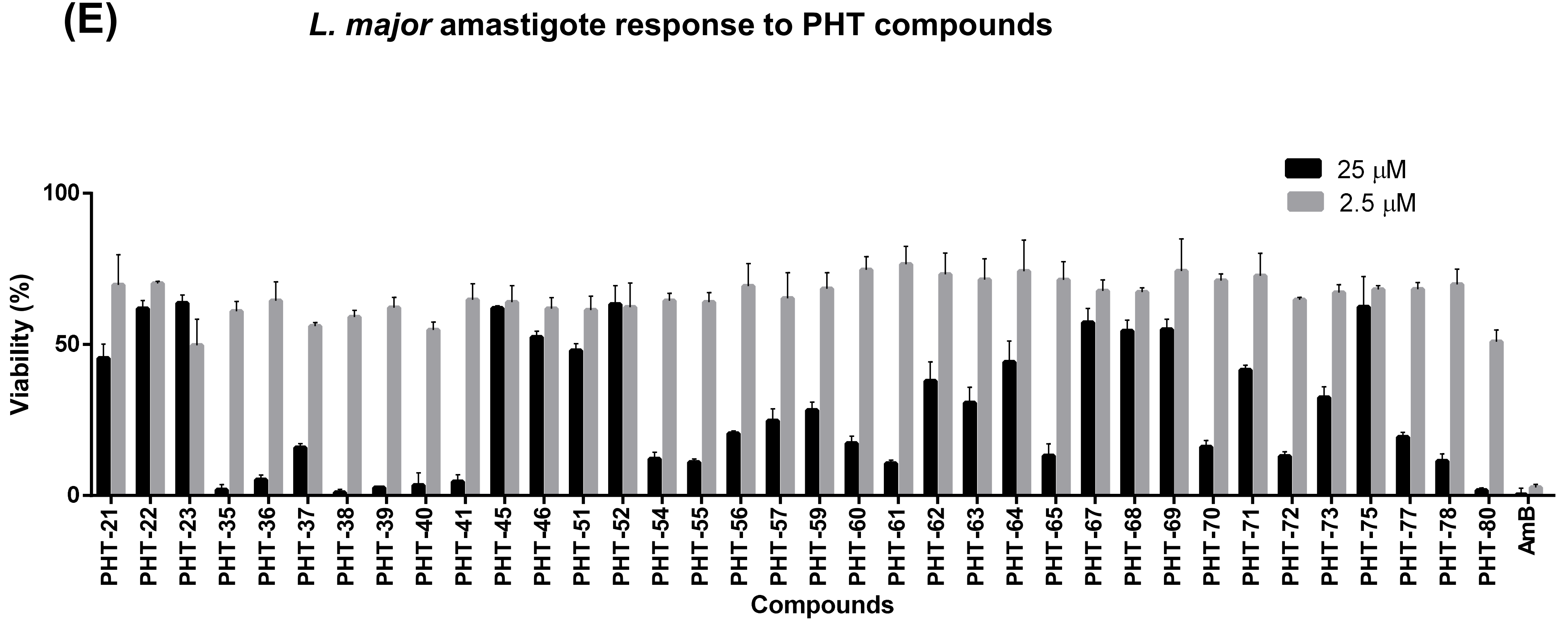

L. major amastigote response to PHT compounds
25 μM
2.5 μM
Viability (%)
100
50
0
PHT-21
PHT-22
PHT-23
PHT-35
PHT-36
PHT-37
PHT-38
PHT-39
PHT-40
PHT-41
PHT-45
PHT-46
PHT-51
PHT-52
PHT-54
PHT-55
PHT-56
PHT-57
PHT-59
PHT-60
PHT-61
PHT-62
PHT-63
PHT-64
PHT-65
PHT-67
PHT-68
PHT-69
PHT-70
PHT-71
PHT-72
PHT-73
PHT-75
PHT-77
PHT-78
PHT-80
AmB
Compounds

(F) ***L. major* amastigote response to LTC compounds 25 μM**

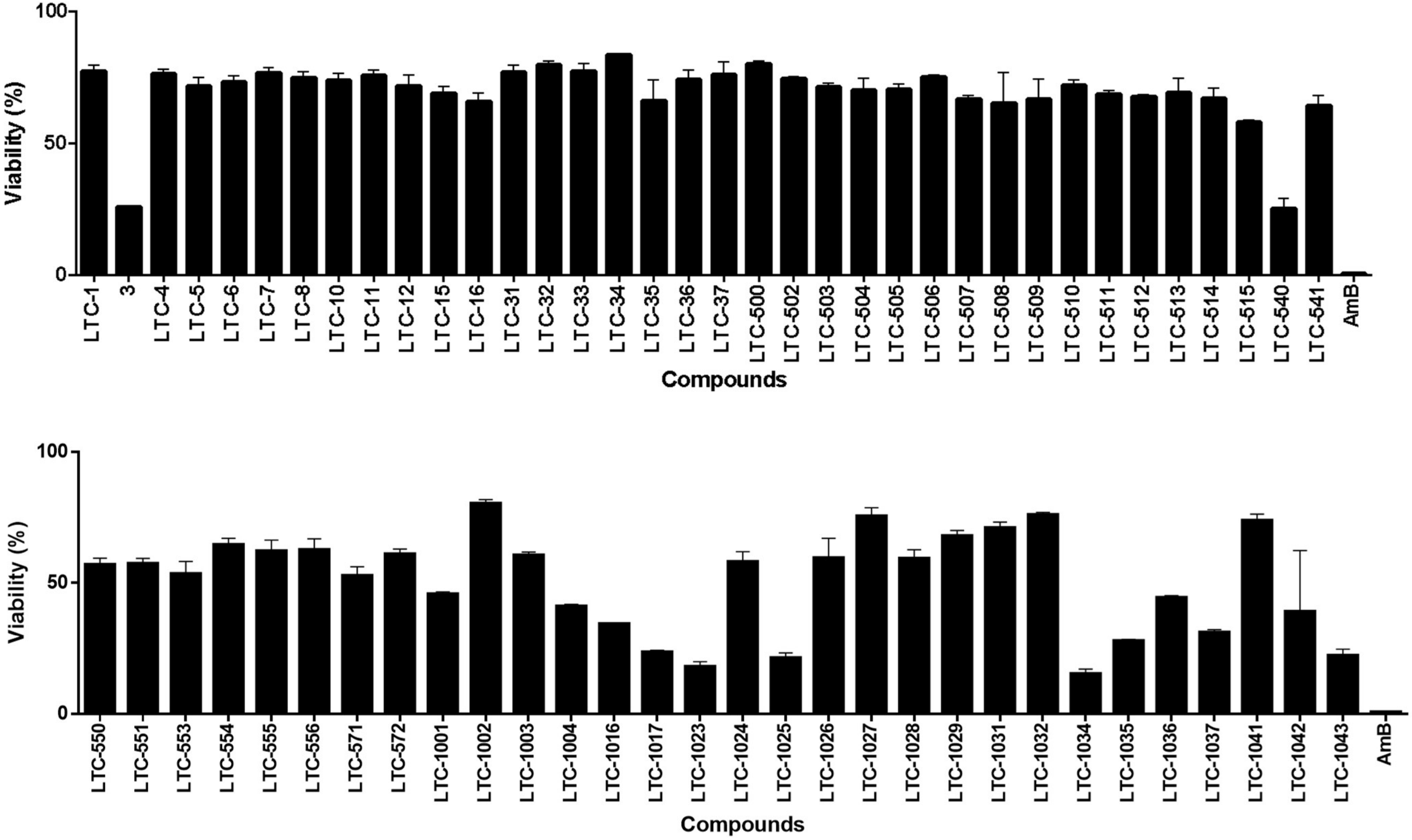

(G) *L. major* amastigote response to LTC compounds at 2.5 μM

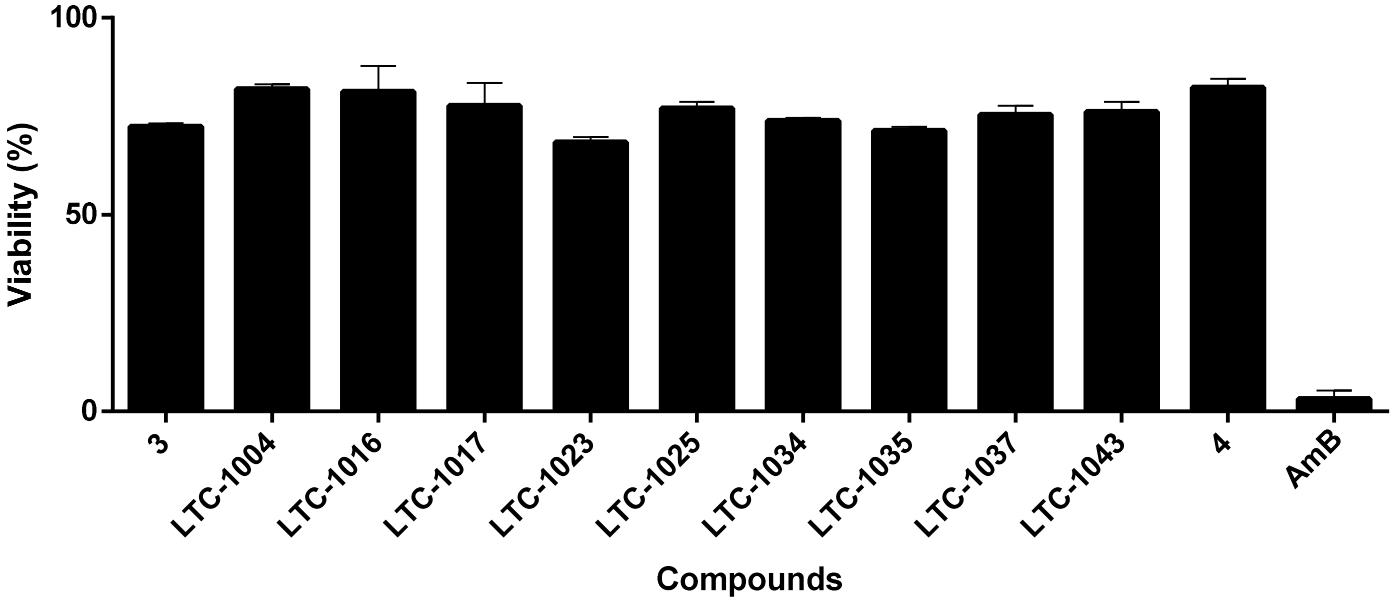

(H)

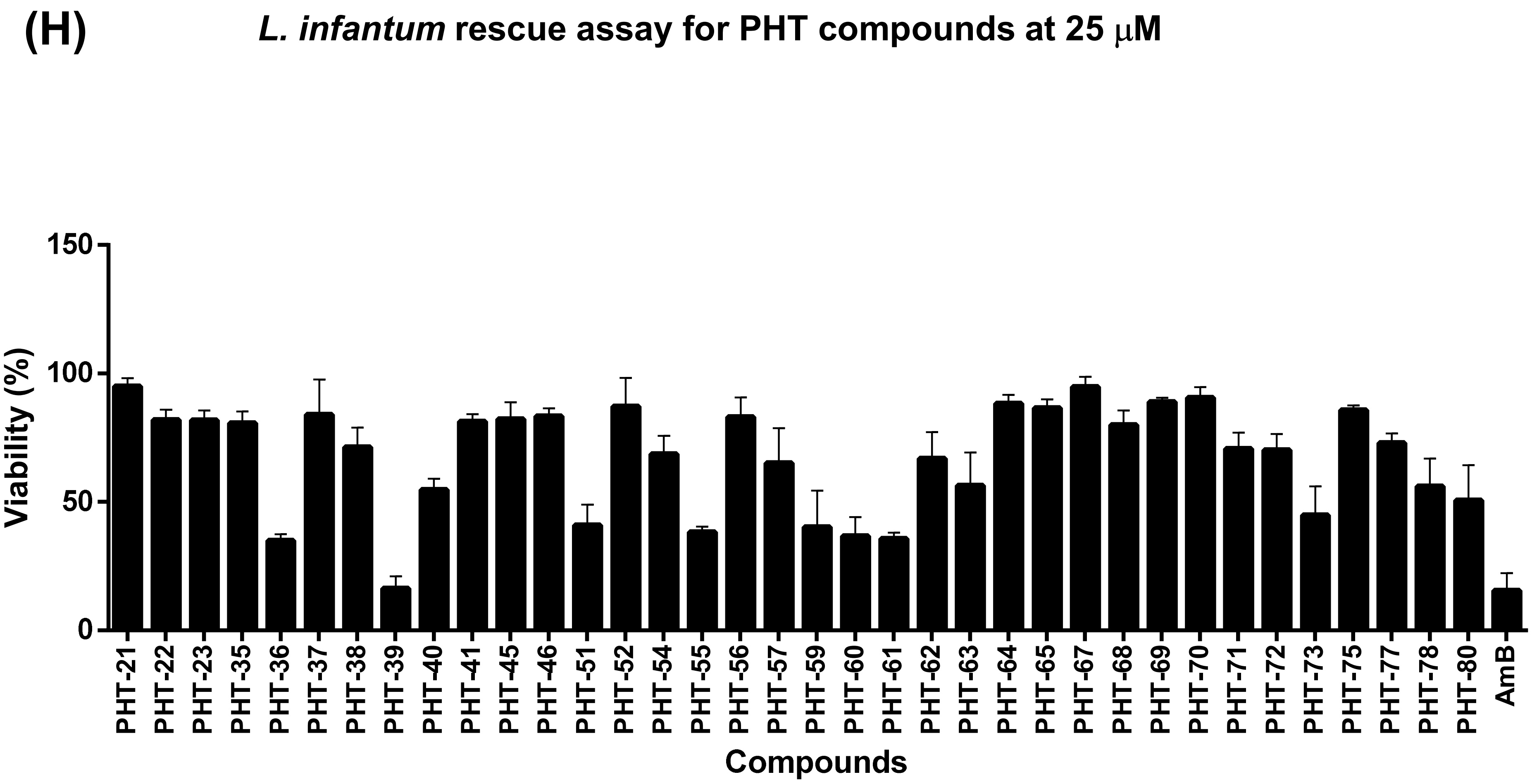
L. infantum rescue assay for PHT compounds at 25 μM
Viability (%)
150
100
50
0
PHT-21
PHT-22
PHT-23
PHT-35
PHT-36
PHT-37
PHT-38
PHT-39
PHT-40
PHT-41
PHT-45
PHT-46
PHT-51
PHT-52
PHT-54
PHT-55
PHT-56
PHT-57
PHT-59
PHT-60
PHT-61
PHT-62
PHT-63
PHT-64
PHT-65
PHT-67
PHT-68
PHT-69
PHT-70
PHT-71
PHT-72
PHT-73
PHT-75
PHT-77
PHT-78
PHT-80
AmB
Compounds

(I)

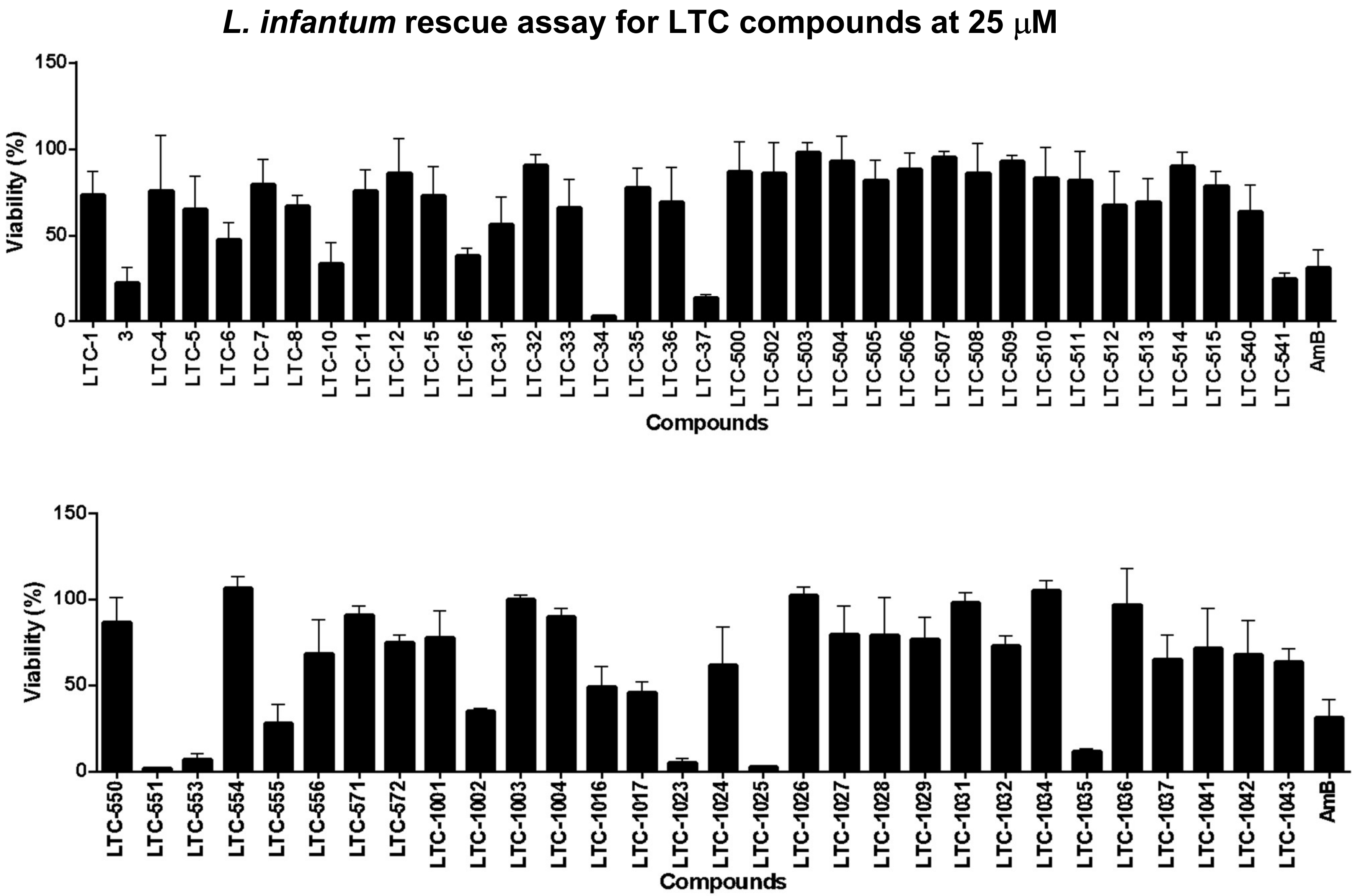
L. infantum rescue assay for LTC compounds at 25 μM
Viability (%)
150
100
50
0
LTC-1
3
LTC-4
LTC-5
LTC-6
LTC-7
LTC-8
LTC-10
LTC-11
LTC-12
LTC-15
LTC-16
LTC-31
LTC-32
LTC-33
LTC-34
LTC-35
LTC-36
LTC-37
LTC-500
LTC-502
LTC-503
LTC-504
LTC-505
LTC-506
LTC-507
LTC-508
LTC-509
LTC-510
LTC-511
LTC-512
LTC-513
LTC-514
LTC-515
LTC-540
LTC-541
AmB
Compounds
Viability (%)
150
100
50
0
LTC-550
LTC-551
LTC-553
LTC-554
LTC-555
LTC-556
LTC-571
LTC-572
LTC-1001
LTC-1002
LTC-1003
LTC-1004
LTC-1016
LTC-1017
LTC-1023
LTC-1024
LTC-1025
LTC-1026
LTC-1027
LTC-1028
LTC-1029
LTC-1031
LTC-1032
LTC-1034
LTC-1035
LTC-1036
LTC-1037
LTC-1041
LTC-1042
LTC-1043
AmB
Compounds

(J)

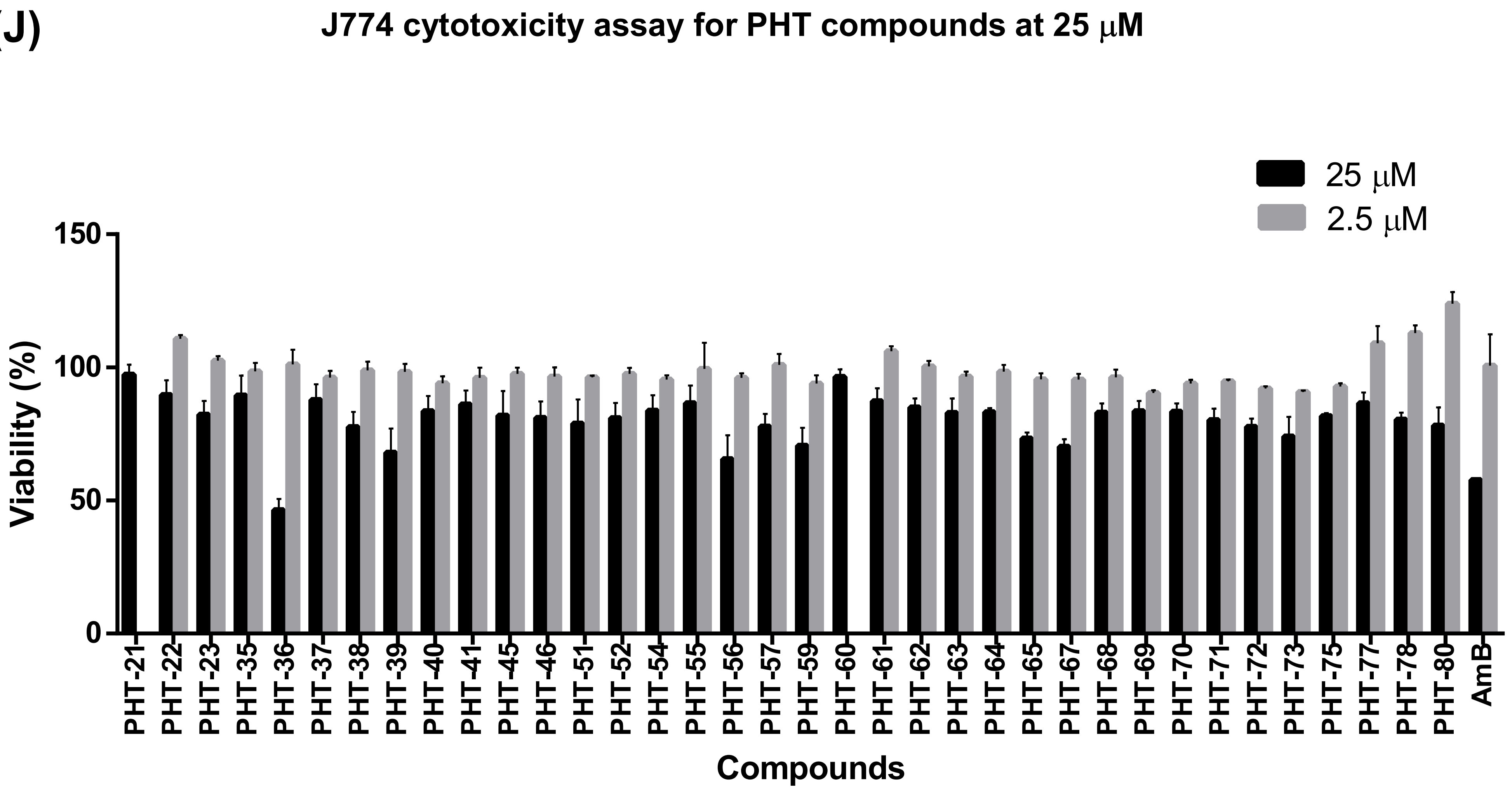

J774 cytotoxicity assay for PHT compounds at 25 μM
25 μM
2.5 μM
150
100
50
0
Viability (%)
PHT-21
PHT-22
PHT-23
PHT-35
PHT-36
PHT-37
PHT-38
PHT-39
PHT-40
PHT-41
PHT-45
PHT-46
PHT-51
PHT-52
PHT-54
PHT-55
PHT-56
PHT-57
PHT-59
PHT-60
PHT-61
PHT-62
PHT-63
PHT-64
PHT-65
PHT-67
PHT-68
PHT-69
PHT-70
PHT-71
PHT-72
PHT-73
PHT-75
PHT-77
PHT-78
PHT-80
AmB
Compounds

(K)

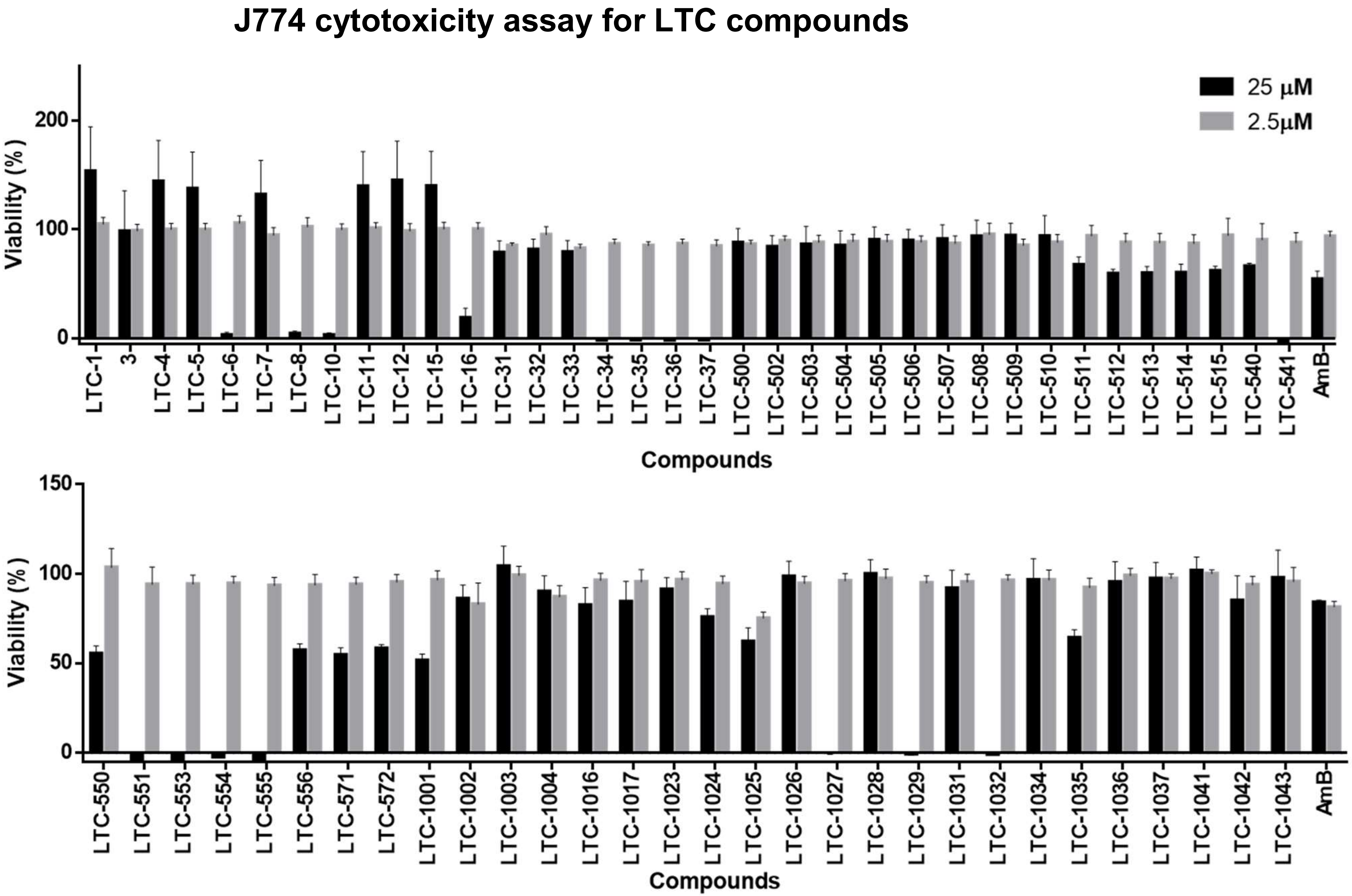
J774 cytotoxicity assay for LTC compounds
25 µM
2.5µM
Viability (%)
200
100
0
LTC-1
3
LTC-4
LTC-5
LTC-6
LTC-7
LTC-8
LTC-10
LTC-11
LTC-12
LTC-15
LTC-16
LTC-31
LTC-32
LTC-33
LTC-34
LTC-35
LTC-36
LTC-37
LTC-500
LTC-502
LTC-503
LTC-504
LTC-505
LTC-506
LTC-507
LTC-508
LTC-509
LTC-510
LTC-511
LTC-512
LTC-513
LTC-514
LTC-515
LTC-540
LTC-541
AmB
Compounds
150
100
50
0
Viability (%)
LTC-550
LTC-551
LTC-553
LTC-554
LTC-555
LTC-556
LTC-571
LTC-572
LTC-1001
LTC-1002
LTC-1003
LTC-1004
LTC-1016
LTC-1017
LTC-1023
LTC-1024
LTC-1025
LTC-1026
LTC-1027
LTC-1028
LTC-1029
LTC-1031
LTC-1032
LTC-1034
LTC-1035
LTC-1036
LTC-1037
LTC-1041
LTC-1042
LTC-1043
AmB
Compounds

(L)

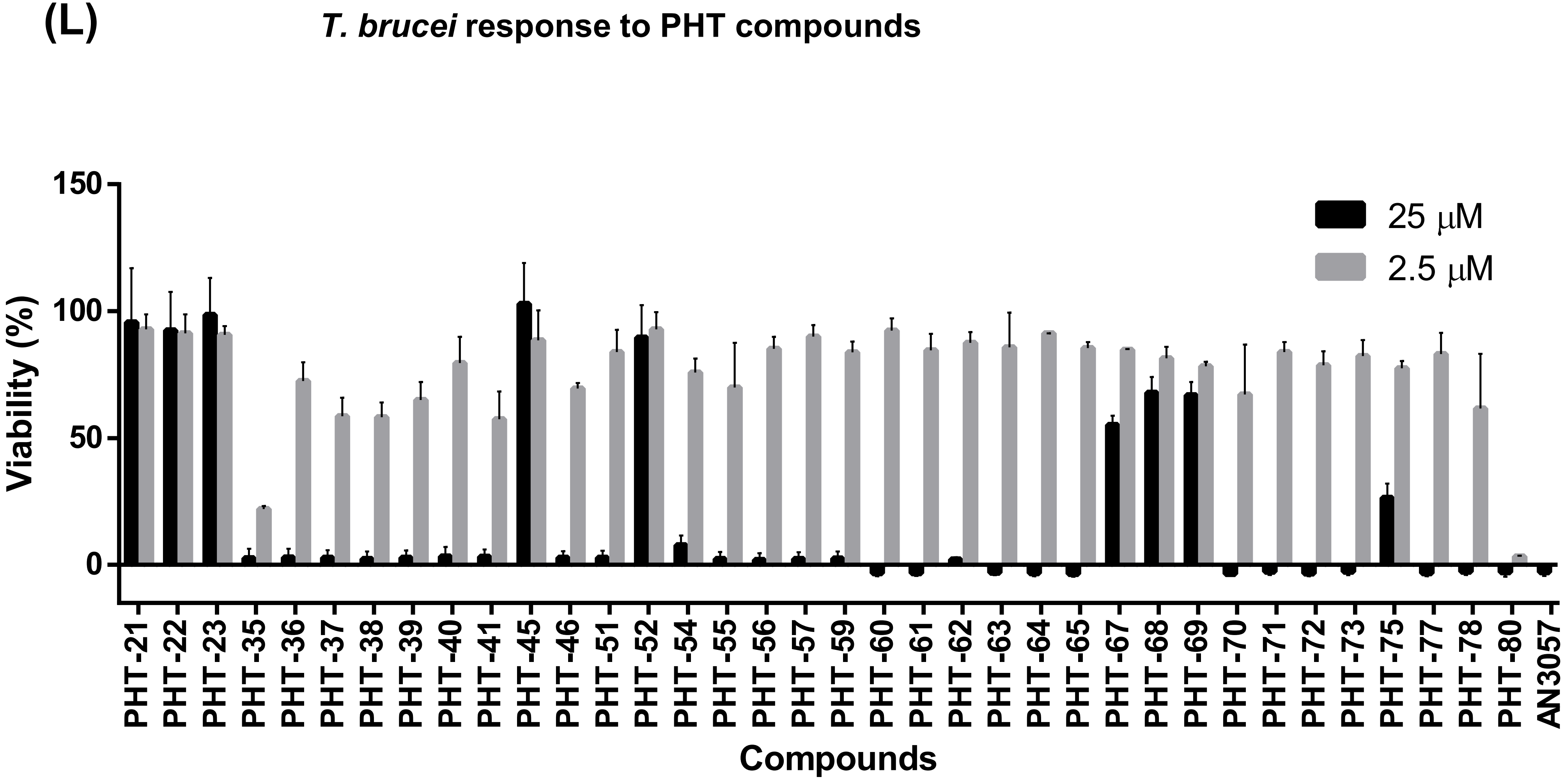

T. brucei response to PHT compounds
150
100
50
0
Viability (%)
25 μM
2.5 μM
PHT-21
PHT-22
PHT-23
PHT-35
PHT-36
PHT-37
PHT-38
PHT-39
PHT-40
PHT-41
PHT-45
PHT-46
PHT-51
PHT-52
PHT-54
PHT-55
PHT-56
PHT-57
PHT-59
PHT-60
PHT-61
PHT-62
PHT-63
PHT-64
PHT-65
PHT-67
PHT-68
PHT-69
PHT-70
PHT-71
PHT-72
PHT-73
PHT-75
PHT-77
PHT-78
PHT-80
AN3057
Compounds

(M)

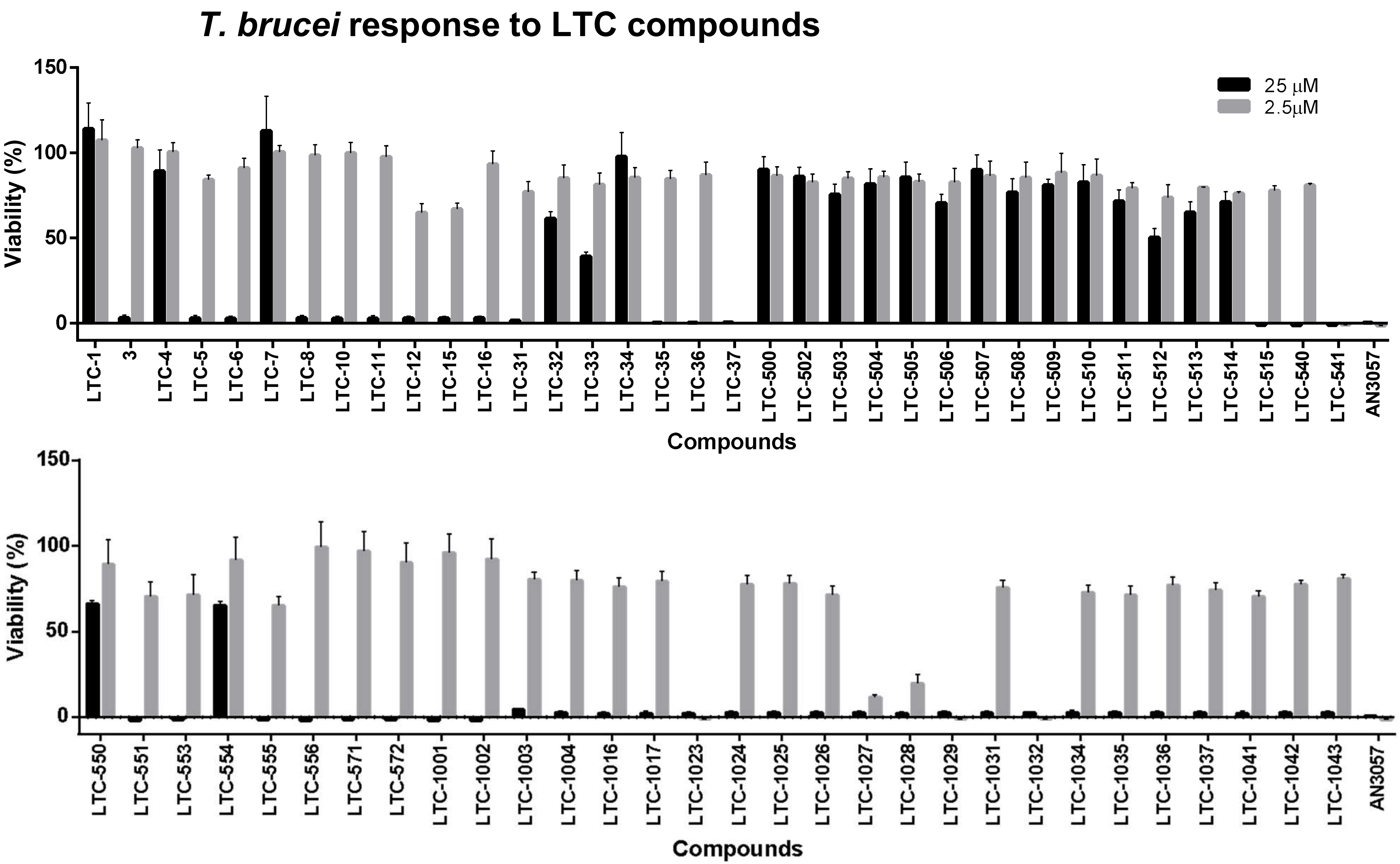
T. brucei response to LTC compounds
25 μM
2.5μM
Viability (%)
150
100
50
0
LTC-1
3
LTC-4
LTC-5
LTC-6
LTC-7
LTC-8
LTC-10
LTC-11
LTC-12
LTC-15
LTC-16
LTC-31
LTC-32
LTC-33
LTC-34
LTC-35
LTC-36
LTC-37
LTC-500
LTC-502
LTC-503
LTC-504
LTC-505
LTC-506
LTC-507
LTC-508
LTC-509
LTC-510
LTC-511
LTC-512
LTC-513
LTC-514
LTC-515
LTC-540
LTC-541
AN3057
Compounds
Viability (%)
150
100
50
0
LTC-550
LTC-551
LTC-553
LTC-554
LTC-555
LTC-556
LTC-571
LTC-572
LTC-1001
LTC-1002
LTC-1003
LTC-1004
LTC-1016
LTC-1017
LTC-1023
LTC-1024
LTC-1025
LTC-1026
LTC-1027
LTC-1028
LTC-1029
LTC-1031
LTC-1032
LTC-1034
LTC-1035
LTC-1036
LTC-1037
LTC-1041
LTC-1042
LTC-1043
AN3057
Compounds

(N)

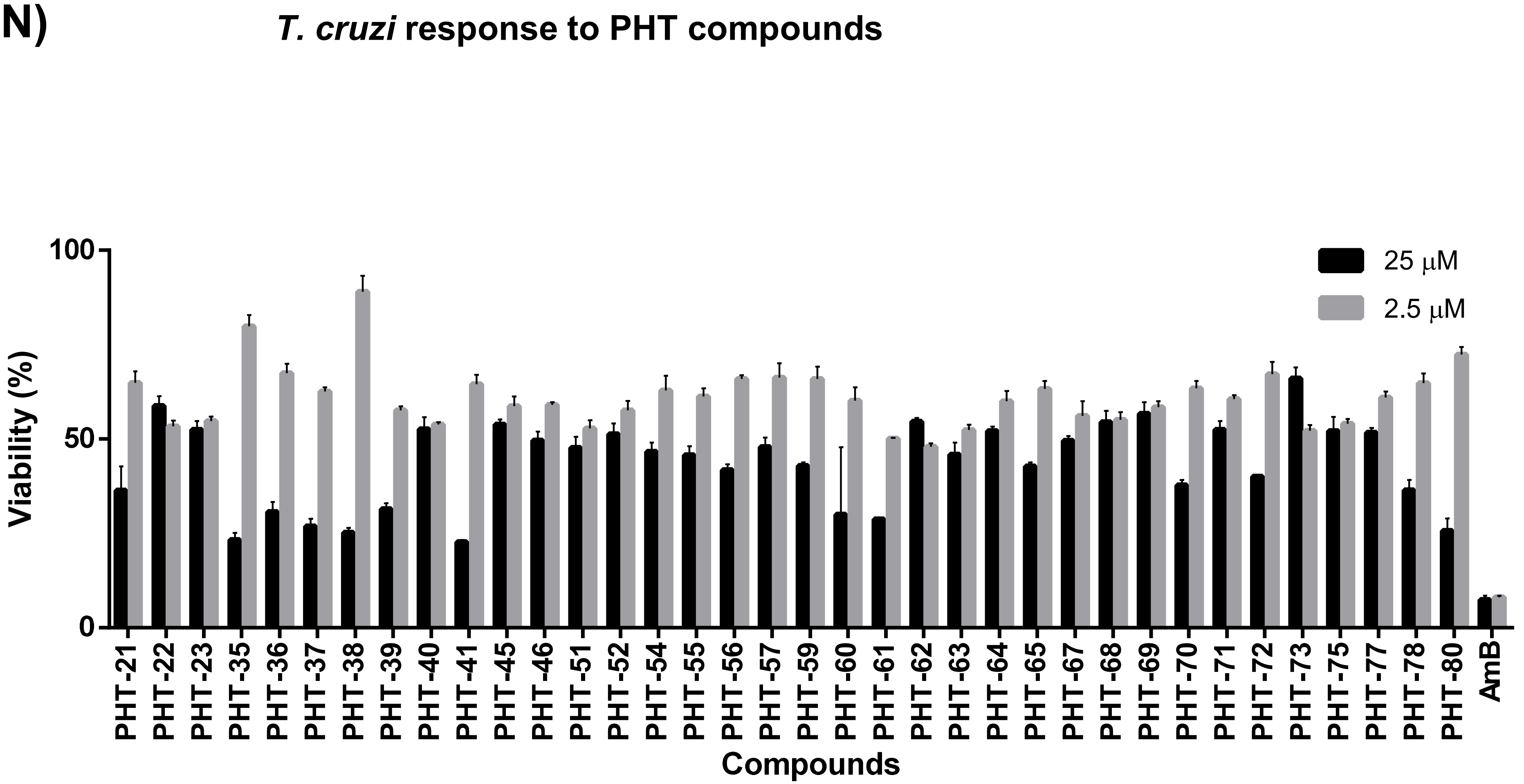
T. cruzi response to PHT compounds
100
50
0
Viability (%)
25 μM
2.5 μM
PHT-21
PHT-22
PHT-23
PHT-35
PHT-36
PHT-37
PHT-38
PHT-39
PHT-40
PHT-41
PHT-45
PHT-46
PHT-51
PHT-52
PHT-54
PHT-55
PHT-56
PHT-57
PHT-59
PHT-60
PHT-61
PHT-62
PHT-63
PHT-64
PHT-65
PHT-67
PHT-68
PHT-69
PHT-70
PHT-71
PHT-72
PHT-73
PHT-75
PHT-77
PHT-78
PHT-80
AmB
Compounds

(O)

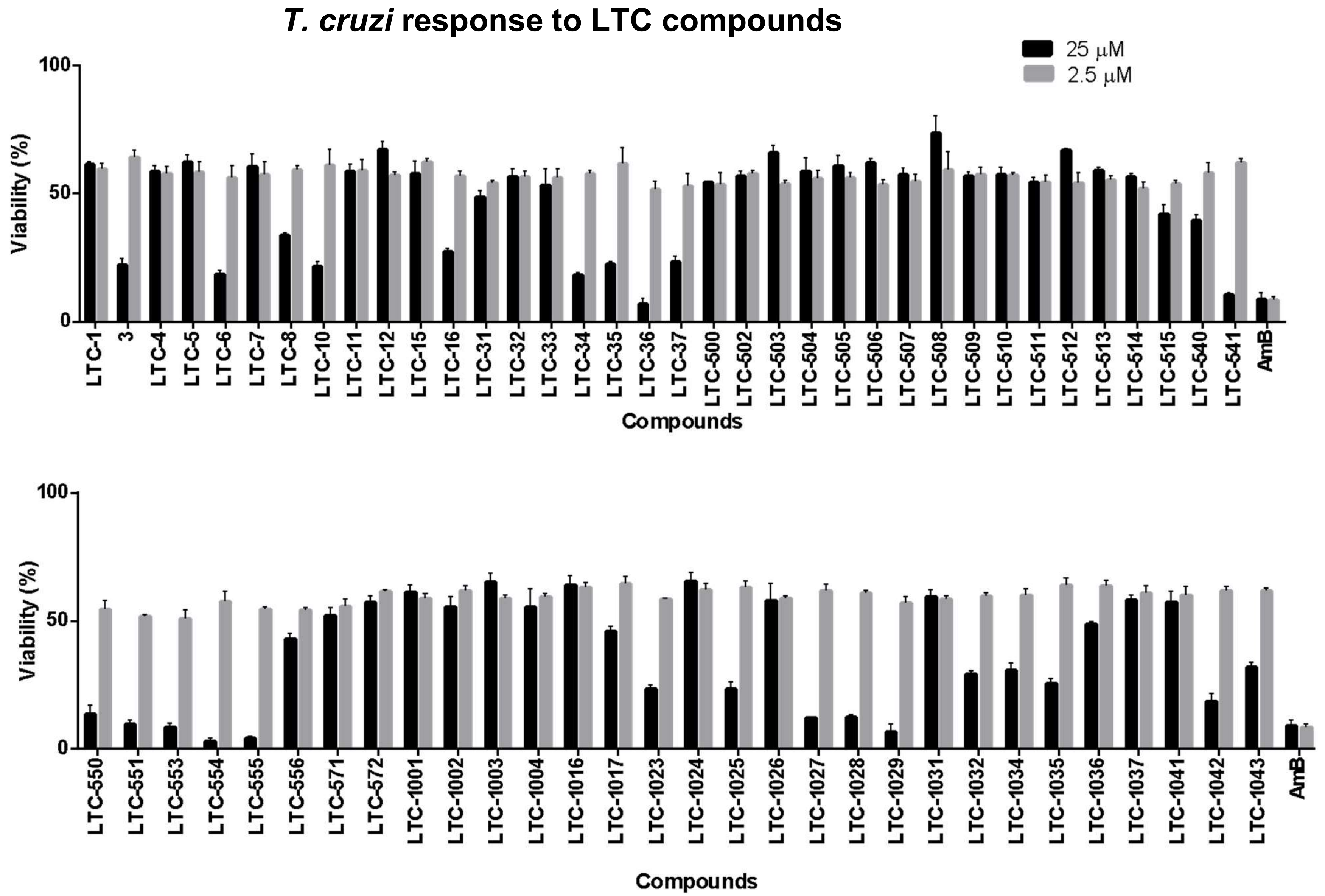
T. cruzi response to LTC compounds
25 μM
2.5 μM
Viability (%)
100
50
0
LTC-1
3
LTC-4
LTC-5
LTC-6
LTC-7
LTC-8
LTC-10
LTC-11
LTC-12
LTC-15
LTC-16
LTC-31
LTC-32
LTC-33
LTC-34
LTC-35
LTC-36
LTC-37
LTC-500
LTC-502
LTC-503
LTC-504
LTC-505
LTC-506
LTC-507
LTC-508
LTC-509
LTC-510
LTC-511
LTC-512
LTC-513
LTC-514
LTC-515
LTC-540
LTC-541
AmB
Compounds
Viability (%)
100
50
0
LTC-550
LTC-551
LTC-553
LTC-554
LTC-555
LTC-556
LTC-571
LTC-572
LTC-1001
LTC-1002
LTC-1003
LTC-1004
LTC-1016
LTC-1017
LTC-1023
LTC-1024
LTC-1025
LTC-1026
LTC-1027
LTC-1028
LTC-1029
LTC-1031
LTC-1032
LTC-1034
LTC-1035
LTC-1036
LTC-1037
LTC-1041
LTC-1042
LTC-1043
AmB
Compounds

(P) ***E. histolytica* response to PHT compounds**

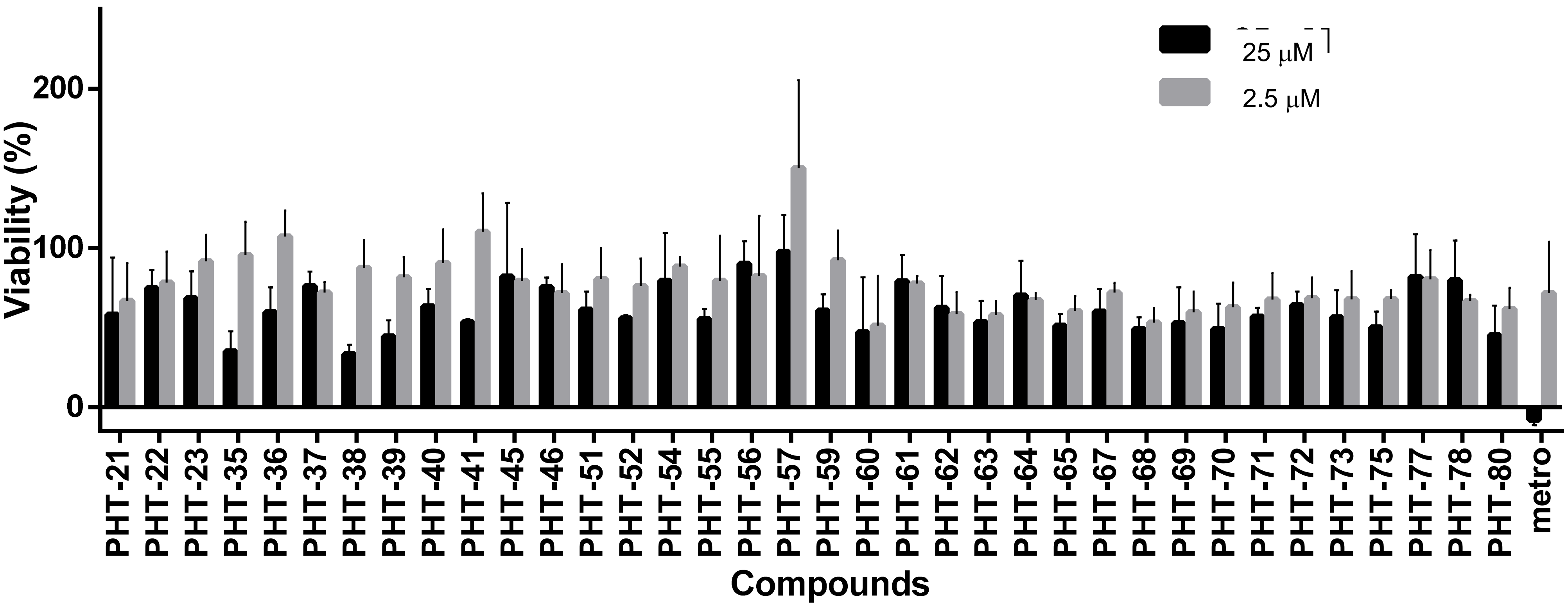

(Q)

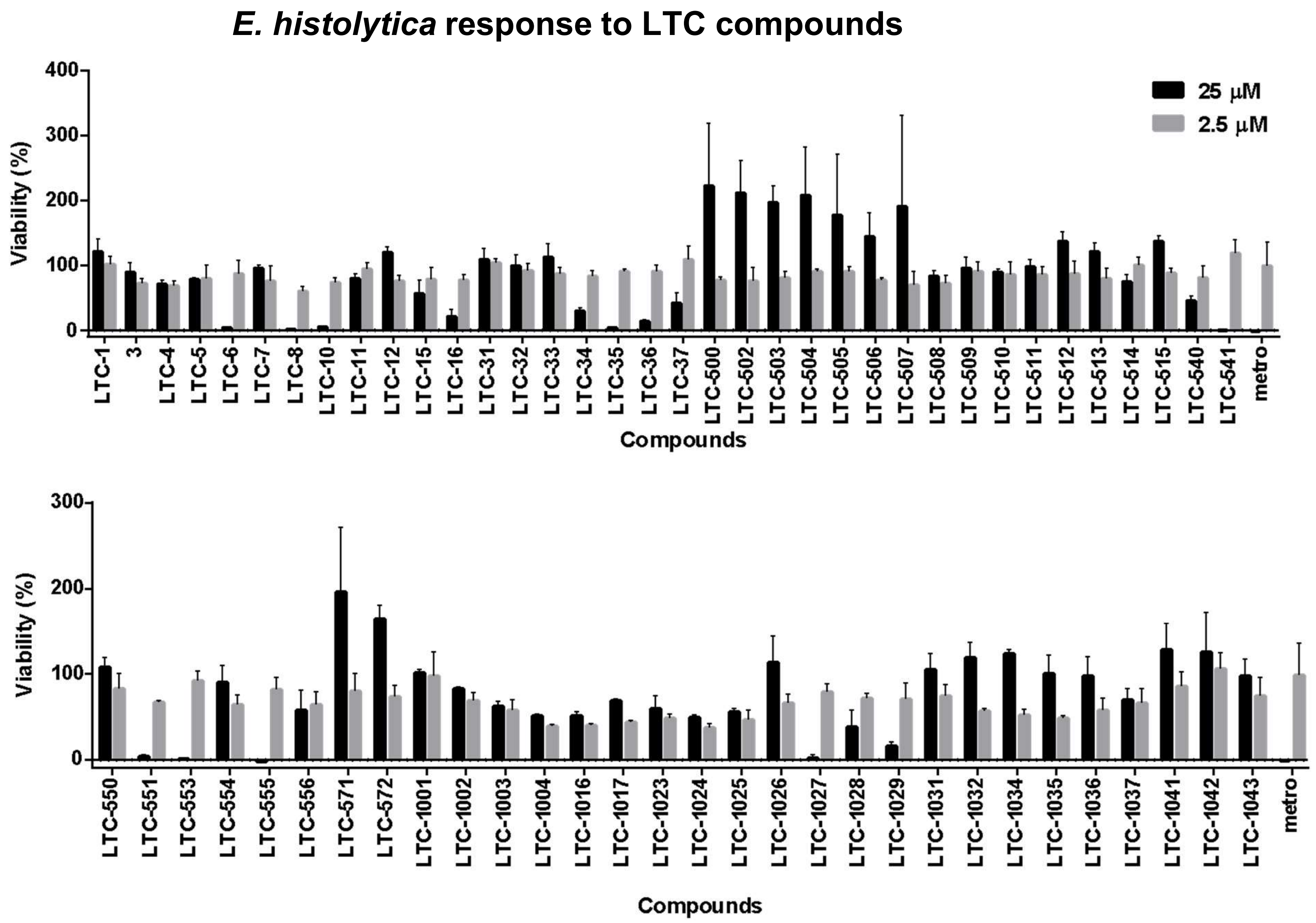
E. histolytica response to LTC compounds
Viability (%)
Compounds
25 μM
2.5 μM
LTC-1
3
LTC-4
LTC-5
LTC-6
LTC-7
LTC-8
LTC-10
LTC-11
LTC-12
LTC-15
LTC-16
LTC-31
LTC-32
LTC-33
LTC-34
LTC-35
LTC-36
LTC-37
LTC-500
LTC-502
LTC-503
LTC-504
LTC-505
LTC-506
LTC-507
LTC-508
LTC-509
LTC-510
LTC-511
LTC-512
LTC-513
LTC-514
LTC-515
LTC-540
LTC-541
metro
LTC-550
LTC-551
LTC-553
LTC-554
LTC-555
LTC-556
LTC-571
LTC-572
LTC-1001
LTC-1002
LTC-1003
LTC-1004
LTC-1016
LTC-1017
LTC-1023
LTC-1024
LTC-1025
LTC-1026
LTC-1027
LTC-1028
LTC-1029
LTC-1031
LTC-1032
LTC-1034
LTC-1035
LTC-1036
LTC-1037
LTC-1041
LTC-1042
LTC-1043
metro
