# Supplementary material for: Phenotypic screening reveals a highly selective phthalimide-based compound with antileishmanial activity
Source: PLoS Negl Trop Dis. 2024 Mar 25;18(3):e0012050. doi: 10.1371/journal.pntd.0012050 (PMC10994559; doi:10.1371/journal.pntd.0012050)
Supplement: S4 Fig — Dose response analyses of hits from PHT and HEA compound screens for L. major promastigotes (A), T. brucei BSF (B), L. major axenic amastigotes (C), cytotoxicity dose response analyses for HepG2 and J774 cells of selected HEA and PHT compounds (D) and the L. infantum rescue assay for selected HEA compounds (E). (PDF) [file pntd.0012050.s004.pdf]

S4 Fig.

A

***L. major* promastigote response**

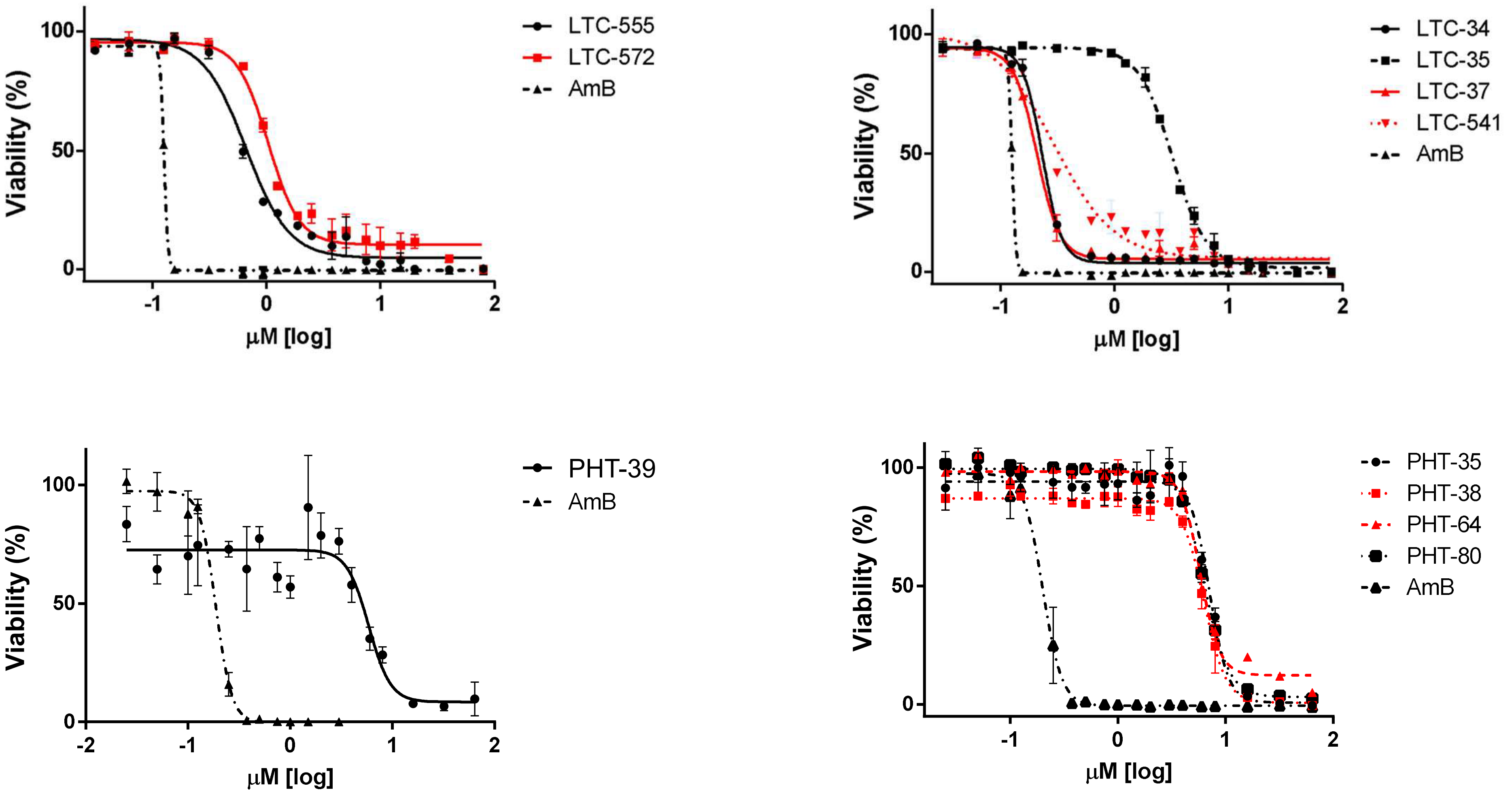

S4 Fig. Dose response analyses of hits from PHT and HEA compound screens for *L. major* promastigotes (A), *T. brucei* BSF (B), *L. major* axenic (C), cytotoxicity dose response analyses for HepG2 and J774 cells of selected HEA and PHT compounds (D) and the *L. infantum* rescue assay for selected HEA compounds (E).

B

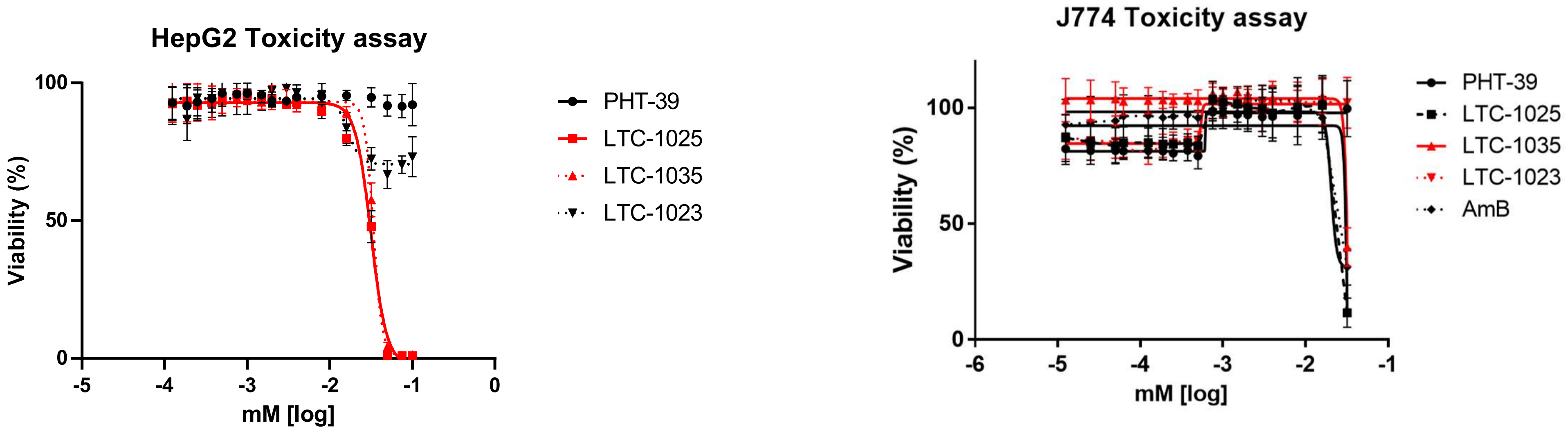
HepG2 Toxicity assay
Viability (%)
100
50
0
-5
-4
-3
-2
-1
0
mM [log]
PHT-39
LTC-1025
LTC-1035
LTC-1023
J774 Toxicity assay
Viability (%)
100
50
0
-6
-5
-4
-3
-2
-1
mM [log]
PHT-39
LTC-1025
LTC-1035
LTC-1023
AmB

C

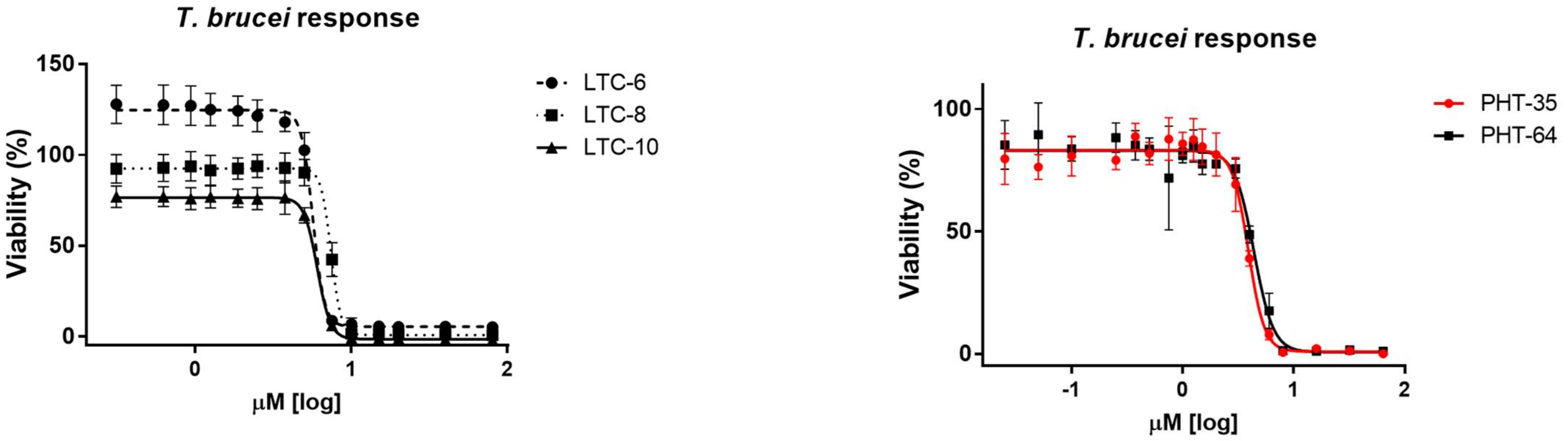
T. brucei response
Viability (%)
150
100
50
0
0
1
2
μM [log]
LTC-6
LTC-8
LTC-10
T. brucei response
Viability (%)
100
50
0
-1
0
1
2
μM [log]
PHT-35
PHT-64

D

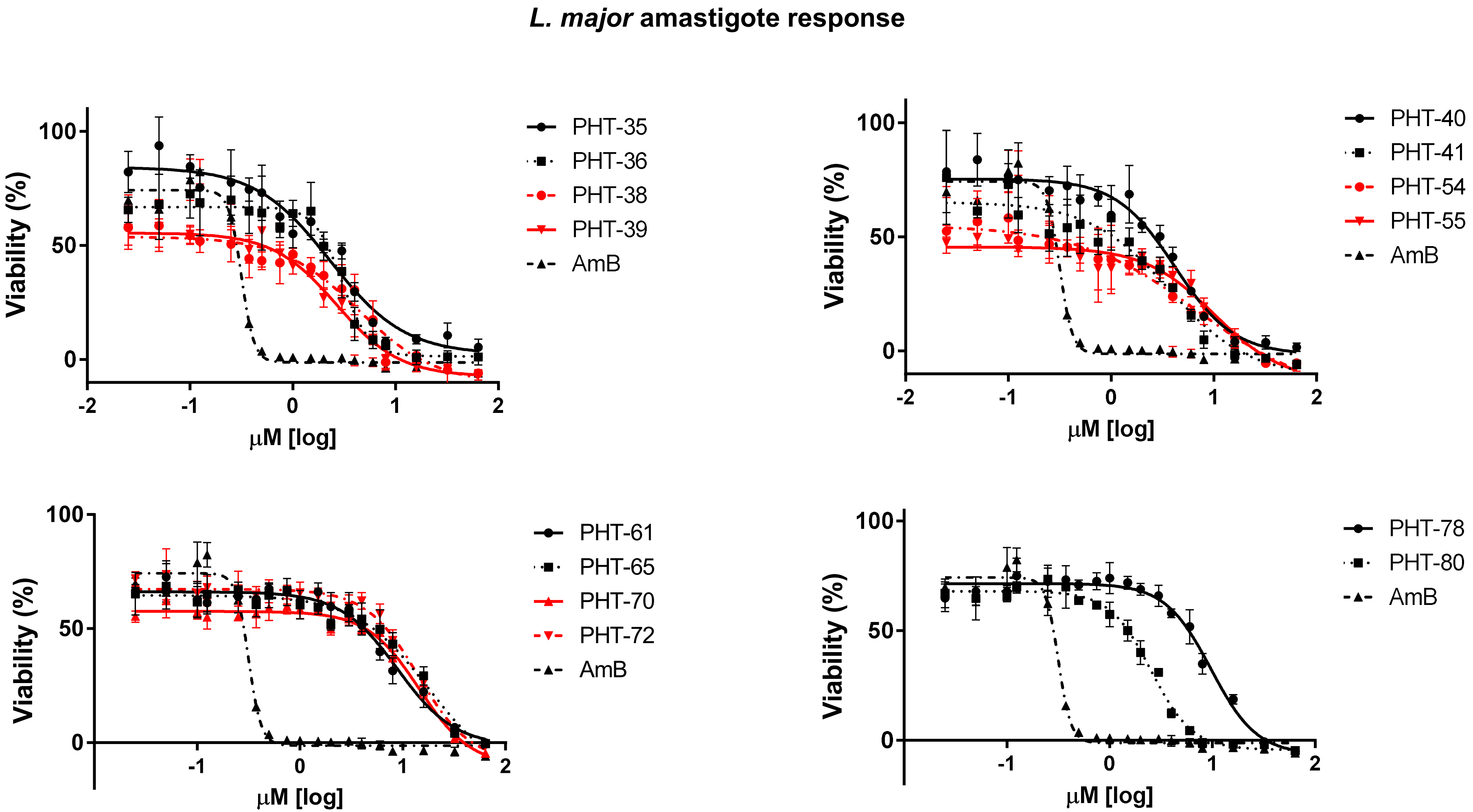
L. major amastigote response
Viability (%)
100
50
0
-2
-1
0
1
2
μM [log]
PHT-35
PHT-36
PHT-38
PHT-39
AmB
PHT-40
PHT-41
PHT-54
PHT-55
AmB
PHT-61
PHT-65
PHT-70
PHT-72
AmB
PHT-78
PHT-80
AmB

E

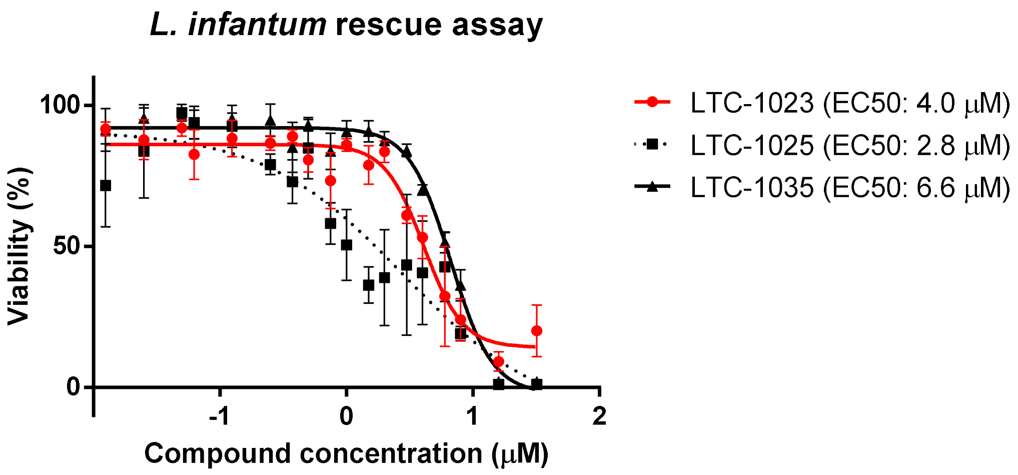

L. infantum rescue assay
Viability (%)
100
50
0
-1
0
1
2
Compound concentration (µM)
LTC-1023 (EC50: 4.0 µM)
LTC-1025 (EC50: 2.8 µM)
LTC-1035 (EC50: 6.6 µM)
