# Supplementary material for: Phenotypic screening reveals a highly selective phthalimide-based compound with antileishmanial activity
Source: PLoS Negl Trop Dis. 2024 Mar 25;18(3):e0012050. doi: 10.1371/journal.pntd.0012050 (PMC10994559; doi:10.1371/journal.pntd.0012050)
Supplement: S5 Fig — Dose response analyses for PHT-39 after targeted knockdown of the two nicotinamidase paralogs Tb927.9.3970 and Tb927.9.4040 (A) and the ClpB1 chaperon Tb927.2.5980 (B), carried out in 3 biological replicates, respectively, did not determine a significant sensitivity shift. (PDF) [file pntd.0012050.s005.pdf]

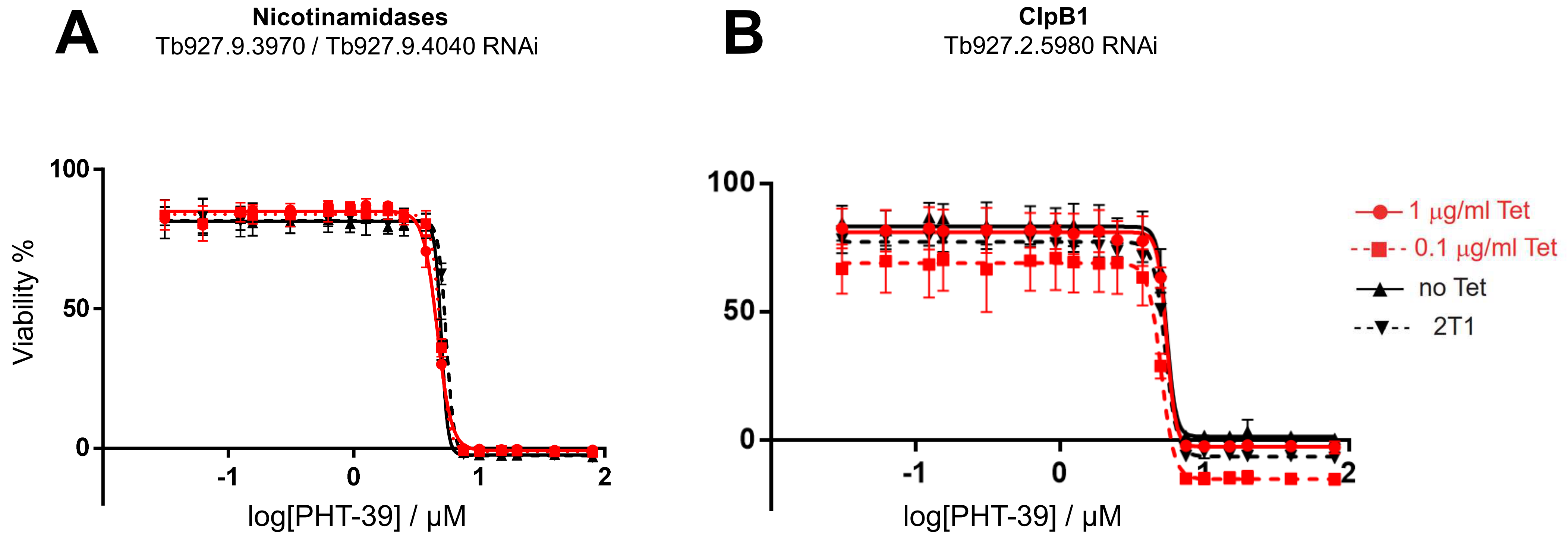

S5 Fig. Dose response analyses for PHT-39 after targeted knockdown of the two nicotinamidase paralogs Tb927.9.3970 and Tb927.9.4040 (A) and the ClpB1 chaperon Tb927.2.5980 (B), carried out in 3 biological replicates, respectively, did not determine a significant sensitivity shift.
