# Supplementary material for: Phenotypic screening reveals a highly selective phthalimide-based compound with antileishmanial activity
Source: PLoS Negl Trop Dis. 2024 Mar 25;18(3):e0012050. doi: 10.1371/journal.pntd.0012050 (PMC10994559; doi:10.1371/journal.pntd.0012050)
Supplement: S6 Fig — One kinetoplast and two nuclei (2N1K) is an aberrant karyotype and not part of the canonical cell cycle. (PDF) [file pntd.0012050.s006.pdf]

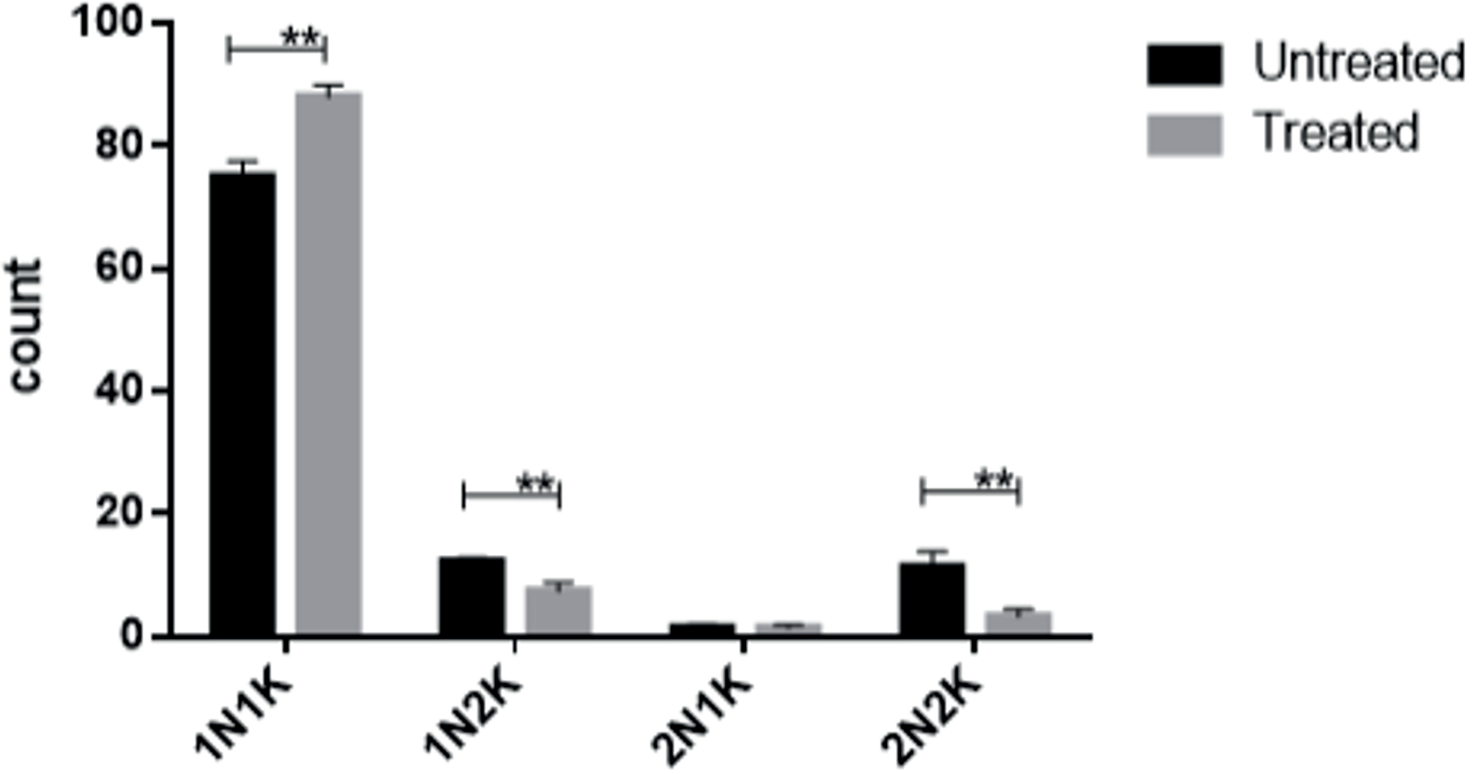

S6 Fig. Karyotype progression after 24h PHT-39 exposure compared to untreated cells (number of cells counted >100; in biological triplicate) employing DAPI-stained nuclear and mitochondrial DNA as cytological markers for cell cycle stages [60] : one nucleus and one kinetoplast (1N1K) indicate G1/S phase, one nucleus and two kinetoplasts (1N2K) indicate G2/M phase, and two nuclei and two kinetoplasts (2N2K) indicate postmitotic cells. One kinetoplast and two nuclei (2N1K) is an aberrant karyotype and not part of the canonical cell cycle.
