# Supplementary material for: Phenotypic screening reveals a highly selective phthalimide-based compound with antileishmanial activity
Source: PLoS Negl Trop Dis. 2024 Mar 25;18(3):e0012050. doi: 10.1371/journal.pntd.0012050 (PMC10994559; doi:10.1371/journal.pntd.0012050)
Supplement: S7 Fig — Cytokine production in supernatant of spleen cells (as in Fig 6) with additional no-antigen (NoAg) control and ConA positive control for (A) IFN-γ, (B) IL-4. Graph shows Mean ± SD. Unpaired student t-test was used for IFN- γ and IL-4. Asterisk indicates the significant difference between values in each group. The *, **, *** and **** = P-value < 0.05, 0.01, 0.001 and 0.0001 respectively. All tests were performed in duplicate and 6 animals per group. F/T: Freeze-thawed whole antigen of L. major cells. (PDF) [file pntd.0012050.s007.pdf]

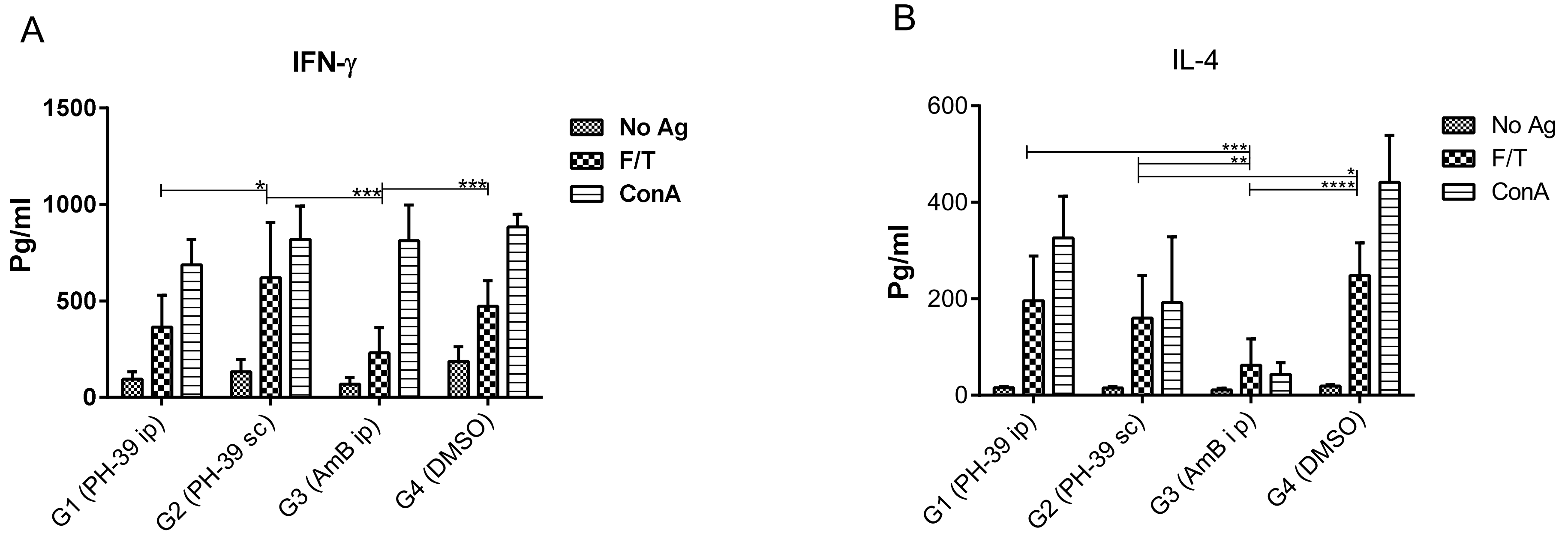

S7 Fig. Cytokine production in supernatant of spleen cells (as in Fig 6) with additional no-antigen (NoAg) control and ConA positive control for (A) IFN-γ, (B) IL-4. Graph shows Mean ± SD. Unpaired student *t*-test was used for IFN- γ and IL-4. Asterisk indicates the significant difference between values in each group. The *, **, *** and **** = *P*-value < 0.05, 0.01, 0.001 and 0.0001 respectively. All tests were performed in duplicate and 6 animals per group. F/T: Freeze-thawed whole antigen of *L. major* cells.
