# Supplementary material for: Phenotypic screening reveals a highly selective phthalimide-based compound with antileishmanial activity
Source: PLoS Negl Trop Dis. 2024 Mar 25;18(3):e0012050. doi: 10.1371/journal.pntd.0012050 (PMC10994559; doi:10.1371/journal.pntd.0012050)
Supplement: S1 Table — (DOCX) [file pntd.0012050.s008.docx]

**S1** **Table: Primers, plasmids and cell lines**

| **Primer** | **Sequence** | **Purpose** |
| --- | --- | --- |
| LIB2f | 5′-TAGCCCCTCGAGGGCCAGT-3′ | RITseq primer |
| LIB2r | 5′-GGAATTCGATATCAAGCTTGGC-3′ | RITseq primer |
| Seq 1 | 5′-AATAGTGGACTCTTGTTCCA-3′ | Confirmation of sense target sequence in stem loop RNAi vector (pRPA^ISL^) |
| Seq 2 | 5′-AAAGGGGGATGTGCTGCAAG-3′ | Confirmation of antisense target sequence in stem loop RNAi vector (pRPA^ISL^) |
| ATPase F | 5’GATCGGGCCCGGTACCTGAACCCTGTGACAGCCATC3’ | Tb927.11.3350 RNAi cloning |
| ATPase R | 5’GATCTCTAGAGGATCCCAACCTACGGTCGAGATCGG3’ | Tb927.11.3350 RNAi cloning |
| ESAG5-F | 5’ GATCGGGCCCGGTACCTTCCCTTGTGTTGGGCCTTT3’ | Tb427.04.810 RNAi cloning |
| ESAG5-R | 5’ GATCTCTAGAGGATCCAGGTAAGCCTCGGGGTACAT3’ | Tb427.04.810 RNAi cloning |
| Nicotinamidase-F | 5’ GATCGGGCCCGGTACC AATCGCGTTGTCGGAGATCA3’ | Tb427tmp.160.2540 RNAi cloning |
| Nicotinamidase-R | 5’ GATCTCTAGAGGATCC AGGTGTGCTCGCTTCTTCTC3’ | Tb427tmp.160.2540 RNAi cloning |
| ClpB-F | 5’ GATCGGGCCCGGTACCGCGCTTCATGCCTGTGTATG3’ | Tb927.2.5980  RNAi cloning |
| ClpB-R | 5’ GATCTCTAGAGGATCCTGCAGCCAAGTCCATTTTGC3’ | Tb927.2.5980  RNAi cloning |

**pZJM-RNAi** plasmid library was a generous gift from Susan Wyllie and David Horn. This collection of plasmids harbouring whole genome of *T. brucei brucei* 427 Lister with a good coverage. It contains ampicillin bacterial selectable marker and an incomplete hygromicine resistance gene [^1^](#_heading=h.30j0zll).

**pRpaSce*** plasmid (Horn lab) carrying a meganuclease gene (SceI) which cuts a rare 18 nucleotide sequence restriction site on chromosome (Chr) 2 of *T. brucei*. The ssDNA provided by meganuclease increase the probability of recombination and site-specific insertion of segments of libraries into the *T. brucei* genome [^1^](#_heading=h.30j0zll).

**pRpa^iSL^** plasmid is for targeted RNAi, applied for hit validation. The plasmid contains two anti-parallel sites flanking lacz locus for cloning of a selected gene to transcribe a stem-loop RNAi molecule. The plasmid harbors ampicillin bacterial selectable marker and a partial hygromycin gene which is completed and functional after insertion of the construct on Chr 2 of 2T1/T7 cells [^1^](#_heading=h.30j0zll).

**2T1/T7** cells are derived from *T. brucei brucei* 427 Lister strain, carrying T7 RNAP gene under the control of double Tet promoter on Chr 1 at tubulin locus. 2T1 cells were generated previously by Alsford et al. (2005) to prevent rDNA position effects in *T. brucei* lines [^2^](#_heading=h.1fob9te). In this cell line, a double Tet repressor from pHD1313 plasmid inserted to tubulin locus on Chr 1 plus a BLE and PAC resistance locus which gives resistance to phleomycin and puromycin, respectively. A T7 RNAP plus a BLA (blasticidin selectable marker) locus was then added to the Chr 1 which partially disrupted BLE locus. 1. Also, in this cell lines, a RRNA (ribosomal RNA spacer) locus was inserted into Chr 2, to increase homologous recombination by approximately 10 times as compared to the wild type strain [^1^](#_heading=h.30j0zll).

**References:**

(1) Glover, L.; Alsford, S.; Baker, N.; Turner, D. J.; Sanchez-Flores, A.; Hutchinson, S.; Hertz-Fowler, C.; Berriman, M.; Horn, D. Genome-scale RNAi screens for high-throughput phenotyping in bloodstream-form African trypanosomes. *Nature protocols* **2015**, *10* (1), 106-133.

(2) Alsford, S.; Kawahara, T.; Glover, L.; Horn, D. Tagging a T. brucei RRNA locus improves stable transfection efficiency and circumvents inducible expression position effects. *Molecular and biochemical parasitology* **2005**, *144* (2), 142-148.
