# Supplementary material for: Phenotypic screening reveals a highly selective phthalimide-based compound with antileishmanial activity
Source: PLoS Negl Trop Dis. 2024 Mar 25;18(3):e0012050. doi: 10.1371/journal.pntd.0012050 (PMC10994559; doi:10.1371/journal.pntd.0012050)
Supplement: S6 Table — Abundance changes for each protein are compared with those observed upon 48h suramin treatment [66] and stage specific changes comparing BSF long slender with stumpy and PCF cells, respectively [54]. (DOCX) [file pntd.0012050.s013.docx]

| **Protein** | **Protein ID** | **Localisation (TrypTag)**  (PMID: 36925446) | **comment** | **PHT-39**  **ratio** | **PHT-39**  **-log_10_*P*-value** | **ratio**  **stumpy/BSF**  (PMID: 26910529) | **ratio**  **PCF/BSF**  (PMID: 26910529) | **suramin**  **ratio**  (PMID: 32354742) | **suramin**  **-log_10_*P*-value**  (PMID: 32354742) |
| --- | --- | --- | --- | --- | --- | --- | --- | --- | --- |
| **Differentiation signalling** | | | | | | | | | |
| PIP39 | Tb927.9.6090 | glycosome, cytoplasm |  | 1.62 | 1.5 | 19.7 | 111.4 | 2.83 | 3.1 |
| **Cytokinesis Initiation Factors** | | | | | | |  |  |  |
| CIF1 (TOEFAZ1) | Tb927.11.15800 | cleavage furrow; flagellum attachment zone | cytokinesis regulator | 0.46 | 0.5 | ND | ND | 0.78 | 0.7 |
| CIF2 | Tb927.9.14290 | cell tip; cilium; cleavage furrow; flagellum attachment zone |  | - ∞ | 0.8 | ND | ND | ND | ND |
| CIF3 | Tb927.10.13100 | cleavage furrow; flagellum attachment zone |  | - ∞ | 3.7 | ND | ND | 1.00 | 0.4 |
| CIF4 | Tb927.10.8240 | cell tip; cleavage furrow; cytoplasm; flagellum attachment zone |  | 0.88 | 0.9 | ND | 2.3 | 0.84 | 0.4 |
| **Flagellar proteins with potential role in cytokinesis** | | | | | | |  |  |  |
| ACS1 | Tb927.7.6180 | flagellar membrane (distal, end) | Part of cap structure present at tips of both new and old flagella (PMID: 28724725) | - ∞ | 1.17 | 1.1 | 1.6 | 0.80 | 0.4 |
| FPRC | Tb927.10.6360 | flagellum attachment zone (distal); cytoplasm (weak) | FAZ-tip-localizing protein required for cytokinesis  (PMID: 30171070) | 0.72 | 0.97 | ND | ND | 1.14 | 0.7 |
|  | Tb927.1.4280 | cortical cytoskeleton; flagellum attachment zone |  | 0.63 | 4.8 | 1.3 | 0.6 | 0.91 | 0.7 |
| SAXO | Tb927.8.6240 | axoneme |  | 0.65 | 2.8 | 0.7 | 1.2 | 0.98 | 3.6 |
| **Mitochondrial F1/Fo ATP synthase** | | | | | | |  |  |  |
| Fo 8 | Tb927.4.3450 | Mitochondrion; kinetoplast |  | ∞ | 2.1 | 9.2 | 24.3 | 1.62 | 0.9 |
| Fo ATBTB12 | Tb927.5.3090 | ND |  | 3.78 | 0.9 | 2.1 | 10.6 | 1.92 | 0.4 |
| Fo k | Tb927.7.840 | ND |  | 3.17 | 0.7 | 3.7 | 8.6 | 1.07 | 0.4 |
| Fo OSCP | Tb927.10.8030 | Mitochondrion; kinetoplast |  | 2.93 | 1.5 | 4.9 | 18.4 | 1.11 | 1.7 |
| Fo i/j | Tb927.3.2880 | Mitochondrion |  | 1.66 | 0.0 | 2.0 | 7.5 | 1.67 | 0.9 |
| Fo ATBTB3 | Tb927.11.6250 | ND |  | 1.53 | 2.6 | 0.1 | 0.3 | 1.77 | 0.7 |
| Fo ATBTB1 | Tb927.10.520 | ND |  | 1.48 | 1.8 | 3.5 | 16.0 | 1.20 | 0.4 |
| Fo ATBTB6 | Tb927.11.1270 | Mitochondrion |  | 1.40 | 1.8 | 2.1 | 8.0 | ND | ND |
| Fo f | Tb927.3.1690 | Mitochondrion; kinetoplast |  | 1.36 | 1.7 | 3.2 | 12.1 | 1.06 | 3.7 |
| Fo ATBTB4 | Tb927.10.9830 | Mitochondrion; kinetoplast |  | 1.36 | 1.7 | 1.9 | 4.9 | 0.88 | 1.1 |
| Fo d | Tb927.5.2930 | ND |  | 1.35 | 1.5 | 4.3 | 13.0 | 1.32 | 0.7 |
| Fo e | Tb927.11.600 | Mitochondrion; kinetoplast |  | 1.09 | 0.3 | ND | 55.7 | 1.21 | 1.4 |
| F1 beta | Tb927.3.1380 | Mitochondrion; kinetoplast |  | 1.33 | 2.1 | 5.7 | 16.0 | 1.57 | 2.1 |
| F1 alpha | Tb927.7.7430;  Tb927.7.7420 | Mitochondrion; kinetoplast |  | 1.31 | 1.9 | 3.0 | 9.8 | 1.62 | 1.9 |
| F1 gamma | Tb927.10.180 | ND |  | 1.23 | 1.8 | 3.7 | 4.0 | 1.62 | 2.2 |
| F1 p18 | Tb927.5.1710 | Mitochondrion; kinetoplast |  | 1.90 | 0.6 | 4.6 | 39.4 | 0.87 | 1.1 |
| Mitochondrial metabolism | | | | | | | | | |
| Proline dehydrogenase | Tb927.7.210 | Mitochondrion; kinetoplast | amino acid metabolism | 1.66 | 1.7 | 55.7 | 84.4 | 4.55 | 1.1 |
| Glutamate  dehydrogenase | Tb927.9.5900 | Mitochondrion; kinetoplast | amino acid metabolism | 1.53 | 1.6 | 18.4 | 42.2 | 3.30 | 1.0 |
| Aconitase | Tb927.10.14000 | cytoplasm, kinetoplast,flagellar cytoplasm, nuclear lumen | tricarboxylic acid cycle | 1.39 | 2.0 | #VALUE! | 9.8 | 2.76 | 1.5 |
| 2-oxoglutarate dehydrogenase E1 component | Tb927.11.1450 | antipodal sites, mitochondrion | tricarboxylic acid cycle | 1.73 | 2.0 | 55.7 | 84.4 | 2.73 | 2.6 |
| 2-oxoglutarate dehydrogenase E1 component | Tb927.11.9980 | Mitochondrion; kinetoplast | tricarboxylic acid cycle | 1.93 | 2.4 | 194.0 | 21.1 | 3.16 | 1.6 |
| succinyl-CoA ligase beta-chain | Tb927.10.7410 | ND | tricarboxylic acid cycle | 1.79 | 2.5 | 19.7 | 10.6 | 1.97 | 1.7 |
| succinyl-CoA synthetase alpha subunit | Tb927.3.2230 | ND | tricarboxylic acid cycle | 1.72 | 2.0 | 39.4 | 32.0 | 1.94 | 2.1 |
| citrate synthase | Tb927.10.13430 | Mitochondrion; kinetoplast | tricarboxylic acid cycle | 1.66 | 1.8 | 4.9 | 7.5 | ND | ND |
| isocitrate dehydrogenase | Tb927.8.3690 | Mitochondrion; kinetoplast | tricarboxylic acid cycle | 1.46 | 2.4 | 4.3 | 3.7 | 1.96 | 1.9 |
| mitochondrial malate dehydrogenase | Tb927.10.2560 | Mitochondrion; kinetoplast | tricarboxylic acid cycle | 1.32 | 0.9 | 13.9 | 119.4 | 1.99 | 1.5 |
| Glycolysis | | | | | | | | | |
| PPDK | Tb927.11.6280 | glycosome |  | ∞ | 0.2 | 32.0 | 776.0 | 2.94 | 1.6 |
|  |  |  |  |  |  |  |  |  |  |
|  |  |  |  |  |  |  |  |  |  |

**S6 Table. Impact of PHT-39 exposure on the abundance of F1Fo-ATPase and cytokinesis initiation factors.**
